# Hedonic eating is controlled by dopamine neurons that oppose GLP-1R satiety

**DOI:** 10.1126/science.adt0773

**Published:** 2025-03-28

**Authors:** Zhenggang Zhu, Rong Gong, Vicente Rodriguez, Kathleen T. Quach, Xinyu Chen, Scott M. Sternson

**Affiliations:** 1 Department of Neurosciences, University of California, San Diego; La Jolla, CA 92093, USA.; 2 Janelia Research Campus, Howard Hughes Medical Institute; Ashburn, VA 20147, USA.; 3 Howard Hughes Medical Institute; University of California, San Diego; La Jolla, CA 92093, USA.

## Abstract

**Introduction:**

Hedonic eating is food ingestion that is driven by palatability rather than bodily need. This can lead to overeating, which in turn may contribute to obesity. Despite the important link between food palatability and hedonic eating, the neural mechanisms controlling this process are poorly understood.

**Rationale:**

Eating behaviors unfold in three phases. The seeking, consumption, and satiety phases initiate, sustain, and terminate feeding, respectively. These phases are coordinated by separate but interacting neural circuits that operate with distinct timing. Although progress has been made to understand the neural basis of food-seeking and satiety, attempts to define neural circuits for palatability-control over the consumption phase show contradictory results.

VTA dopamine (VTA^DA^) neurons are a prominent candidate cell type for promoting palatable food consumption because they are activated by rewards. However, most manipulations of dopamine neurons have been reported to reduce food consumption. To reconcile this discrepancy, we avoided the temporally nonspecific manipulations of previous studies by manipulating VTA^DA^ neuron activity only during consumption, corresponding one phase of their their natural activity pattern.

Another cell type involved in hedonic eating is glutamatergic neurons in a brain region called the periLC (periLC^VGLUT2^ neurons). Some of these neurons are inhibited during hedonic eating in a manner scaled by food palatability or hunger state. Although these neurons do not trigger food seeking, their inhibition during consumption prolongs ongoing food intake, particularly with palatable foods. However, the pathway through which these neurons influence food consumption was unknown.

**Results:**

Here, we found that VTA^DA^ neuron activity was time-locked to the duration of food consumption, and this response was scaled by food palatability. These effects were also observed when measuring dopamine release in the nucleus accumbens (NAc), a downstream target of VTA^DA^ neurons. Hunger increased VTA^DA^ neuron activity during consumption, while a sickness state reduced it. When we optogenetically boosted VTA^DA^ neuron activity specifically during consumption, food intake was prolonged, similar to the effect of increasing food palatability. Conversely, inhibition of VTA^DA^ neurons reduced consumption. Highlighting the consumption-specific effect of VTA^DA^ neurons, activation of these neurons when mice were not eating did not cause mice to approach food or increase food intake.

Moreover, we showed that periLC^VGLUT2^ neurons signal to VTA^DA^ neurons to influence consumption via an indirect pathway: periLC^VGLUT2^ neuron axons primarily targeted VTA^VGAT^ neurons, which inhibit VTA^DA^ neurons.

We also found that the anti-obesity drug, semaglutide, initially reduced the duration of palatable food consumption, accompanied by a decrease in VTA^DA^ neuron activity. Optogenetic activation of VTA^DA^ neurons partially counteracted the appetite-lowering effect of semaglutide for palatable food. Additionally, as mice lost weight on semaglutide, VTA^DA^ neuron activity increased, as did palatable food intake, which could be reversed by inhibiting VTA^DA^ neurons.

**Conclusion:**

Collectively, our experiments show that VTA^DA^ neurons are engaged throughout the consumption phase of feeding and play a pivotal role in hedonic eating. Their influence is specifically tied to VTA^DA^ neuron activity during food consumption, mirroring the consumption-specific role of periLC^VGLUT2^ neurons, which are selectively suppressed during food intake and thus participate in a circuit configured to disinhibit VTA^DA^ neurons. These circuit dynamics during palatable food intake upregulate dopamine in the NAc, which is a brain region that modulates the duration of consumption.

VTA^DA^ neurons are engaged during each of the three phases of motivated behavior. This study clarifies the involvement of VTA^DA^ neurons in the consummatory phase of hedonic food intake. We highlight the critical role of VTA^DA^ neuron timing to affect eating behavior, where consumption-locked activation promotes food intake but nonspecific activation either has no effect on feeding (our results) or reduces it (prior reports). VTA^DA^ neurons control the duration of ongoing food consumption, and this mechanism fills a gap in our understanding of appetite control by palatable food, which may provide insights into the development and treatment of obesity.

## Abstract (summary)

Hedonic eating is food consumption driven by palatability without physiological need. However, neural control of palatable food intake is poorly understood. We discovered that hedonic eating is controlled by a neural pathway from the peri-locus coeruleus to the ventral tegmental area (VTA). Using photometry-calibrated optogenetics, we found that VTA dopamine (DA) neurons encode palatability to bidirectionally regulate hedonic food consumption. VTA^DA^ neuron responsiveness was suppressed during food consumption by semaglutide, a glucagon-like peptide receptor 1 (GLP-1R) agonist anti-obesity drug. Mice recovered palatable food appetite and VTA^DA^ neuron activity during repeated semaglutide treatment, which was reversed by consumption-triggered VTA^DA^ neuron inhibition. Thus, hedonic food intake activates VTA^DA^ neurons, which sustain further consumption, a mechanism that opposes appetite reduction by semaglutide.

## Graphical Abstract

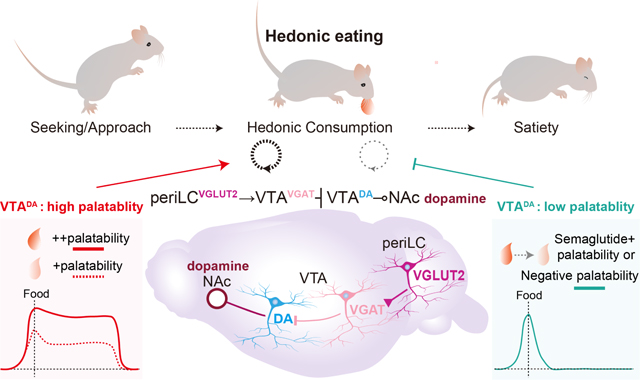

**Control of hedonic eating by dopamine neurons.** The consumption phase of appetite involves sensory feedback to sustain hedonic eating, which is independent of physiological need. VTA^DA^ neurons participate in a circuit (periLC^VGLUT2^→VTA^VGAT^⊣VTA^DA^⊸NAc dopamine) in which activity is increased by positive palatability (left) or decreased by negative palatability or an anti-obesity drug (right). VTA^DA^ neurons are directly involved in regulating the duration of food consumption and have an important role in hedonic eating.

Palatability is associated with food pleasantness and prolongs consumption bouts (1), which can lead to overeating (2). Consumption is sustained by a palatable taste regardless of the physiological energetic state, which is sometimes called the “salted-nut phenomenon” (3) or hedonic eating. The neural circuit mechanisms underlying hedonic eating are poorly understood.

Eating behaviors unfold in three phases: seeking, consummatory, and satiety which are coordinated by separate but interacting neural circuits (4, 5) that initiate, sustain, and terminate feeding, respectively (fig. S1). The food-seeking phase is controlled by homeostatic signals from the body to the brain (6–8) as well as learning about environmental cues that predict food rewards (9). The satiety phase eventually terminates a meal as well as the tendency to initiate feeding (10, 11) due to peripheral signals that can be recapitulated by anti-obesity drugs, such as GLP-1R agonists (12, 13). A notable gap in this framework is how the intervening consummatory phase sustains palatable food consumption in the absence of physiological energetic needs.

VTA dopamine (VTA^DA^) neurons are a candidate cell type for promoting palatable food consumption. Dopamine levels correlate with pleasantness ratings in humans (14), and VTA^DA^ neuron activity increases in response to rewards (15, 16). Accordingly, VTA^DA^ neuron activation is rewarding and can promote place (17), flavor, and nutrient preferences (18). While these studies suggest that VTA^DA^ neuron activity correlates with palatable food consumption, evidence from causal perturbation studies of VTA^DA^ neurons indicates the opposite. VTA^DA^ neuron activation has been consistently found to decrease intake of sucrose (19, 20) and food (21), which directly contradicts the assumption that these neurons promote palatable food consumption. Similarly, food intake decreases when dopamine is elevated by selective dopamine reuptake inhibition or addictive drugs (21–24). These discrepancies have not been reconciled. Instead, VTA^DA^ neurons are primarily associated with reward-prediction error firing in response to cues that predict rewards (25–27) or long-timescale nutrient feedback reinforcement (28). Although VTA^DA^ neurons are clearly involved in reinforcing cues associated with food (29, 30), there has been little evidence to support an active role in VTA^DA^ neurons in the consummatory phase of palatable food.

Recently, we discovered that the peri-locus coeruleus (periLC), a brain region surrounding the locus coeruleus, controls the consumption of palatable food, primarily through *Slc17a6* (*Vglut2*)-expressing glutamatergic neurons (31). This area, distinct from locus coeruleus noradrenergic neurons, is downstream of key homeostatic cell types, such as Agouti-related protein (AGRP), paraventricular hypothalamus (PVH), and lateral hypothalamic area gamma-aminobutyric acid (LHA^GABA^) neurons (31–33). A subset of periLC^VGLUT2^ neurons is inhibited during the consummatory phase of eating, and the inhibition magnitude is scaled by palatability. Inhibition of periLC^VGLUT2^ neurons is rewarding and prolongs the duration of food consumption without affecting food seeking (31). PeriLC neurons project axons to the hindbrain and forebrain (34).

## Results

### periLC^VGLUT2^ →VTA circuit controls palatable food consumption

**Fig. 1.**
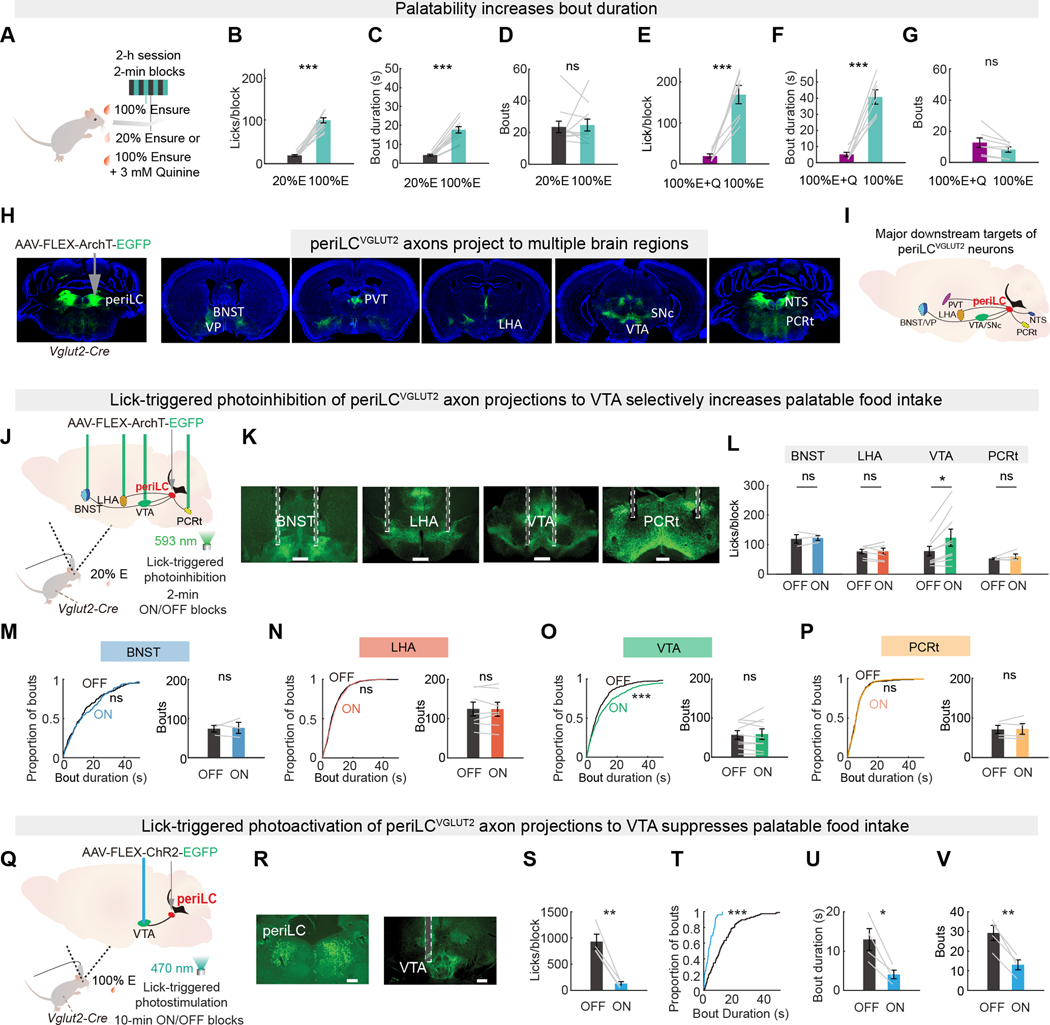
Closed-loop photoinhibition of periLC^VGLUT2^ axon projections to the VTA prolongs consumption. (A) Feeding bout analysis to compare consumption of higher and lower palatability food available in alternating 2-minute blocks (teal and grey blocks, respectively). E: Ensure. (B-G) Higher palatability 100% Ensure leads to more food intake (B, E), longer bout duration (C, F), and a similar number of bouts initiated (D, G) compared to lower palatability diluted Ensure (B-D) (n = 11 mice) or 100% Ensure adulterated with 3 mM quinine (E-G) (n = 7 mice). (H) Cre-dependent expression of ArchT-EGFP in periLC neurons in *Vglut2-IRES-Cre* mice and EGFP fluorescence in downstream brain regions. (I) Summary of periLC^VGLUT2^ neuron axon projection targets. (J) Schematic of periLC^VGLUT2^ axon projection photoinhibition at BNST, LHA, VTA, PCRt during alternating ON and OFF blocks of lick-triggered axon photoinhibition while consuming lower palatability food. (K) ArchT-EGFP fluorescence and highlighted bilateral optical fiber tracts in the BNST, LHA, VTA, and PCRt. Scale bar, 500 μm. (L) Mean licks per block for food in alternating ON and OFF blocks during lick-triggered photoinhibition of periLC^VGLUT2^ axons projections to BNST, LHA, VTA, PCRt (paired t-test, n = 3,6,9,4 mice). (M-P) Feeding bout analysis of laser-ON and laser-OFF blocks for lick-triggered photoinhibition of periLC^VGLUT2^ axons in the BNST, LHA, VTA, and PCRt (KS-test and paired t-test, n = 3,6,9,4 mice). (Q) Schematic of lick-triggered photoactivation of periLC^VGLUT2^→VTA axon projections during feeding. (R) EGFP expression in the periLC and fiber tracts in the VTA. Scale bar, 500 μm. (S-V) Lick-triggered photoactivation of periLC^VGLUT2^ axon projections to the VTA suppressed food intake (S), reduced bout duration (T-U), and reduced bout initiation (V) (KS-test and paired t-test, n = 4). Data are represented as mean ± SEM. ns p>0.05, *p<0.05, **p<0.01, ***p<0.001. Statistical details are in Table S1.

The food-seeking and consummatory phases of eating (5, 29, 35, 36) (fig. S1A) are distinguished by analyzing the microstructure of feeding behavior. The number of eating bouts is associated with seeking food (29, 36), and the duration of a feeding bout is a well-validated behavioral metric of palatability in the consummatory phase (37–40). We examined feeding microstructure in a self-paced hedonic feeding task (see Methods and fig. S2, A to E). Mice were tested with access to a sweet, highly palatable liquid food source and the same food diluted to have lower palatability (100% vs 20% Ensure) in alternating 2-min blocks during 2-h sessions (Fig. 1A). Food consumption rate and bout duration were greater for the higher palatability food (Fig. 1, B and C, and fig. S2, F to H), but the number of bouts initiated (Fig. 1D) was not significantly different. This relationship was maintained when palatability was reduced by adulterating 100% Ensure with quinine (3 mM) and nutrient content was constant (Fig. 1, A and E to G, and fig. S2, I to K). Because we previously found that periLC^VGLUT2^ neuron inhibition also prolongs the duration of a consumption bout without increasing bout number (31), we used this feeding microstructure analysis to identify brain regions that are part of a neural circuit mediating palatability-driven hedonic food intake.

We identified brain regions downstream of periLC^VGLUT2^ neurons by expressing a Cre-dependent ArchT-EGFP transgene (41, 42) (AAV- FLEX-ArchT-EGFP) targeted to the periLC in *Vglut2-IRES-Cre* male and female mice (Fig. 1H). Several subcortical areas contained EGFP fluorescence in axons from periLC^VGLUT2^ neurons (Fig. 1, H, and I), and some of these areas are associated with eating and drinking behavior (9, 43). Next, we investigated the function of axon projections to areas associated with eating: LHA, bed nucleus of the stria terminalis (BNST), parvocellular reticular area (PCRt), and VTA. We used optogenetic axonal inhibition during eating (Fig. 1J) by implanting optical fibers bilaterally over ArchT-expressing periLC^VGLUT2^ axon projections (Fig. 1K). Because inhibition of periLC^VGLUT2^ neurons promotes palatable food intake, we tested the hypothesis that inhibition of the axon projections of periLC^VGLUT2^ neurons solely during food consumption enhances palatability-associated feeding. We compared the consumption of lower palatability food (20% Ensure) paired with lick-triggered (closed-loop, such that mouse behavior elicits neural perturbation) photoinhibition blocks (laser-ON) alternating with photoinhibition-free blocks (laser-OFF) (see Methods “lick-contingent optogenetics” and fig. S3, A and B). This protocol enables temporally precise, within-session analysis of the effects of neuronal perturbations on feeding microstructure (31). Closed-loop lick-contingent photoinhibition of periLC^VGLUT2^→VTA axon projections increased food intake by selectively prolonging bout duration without significantly altering the number of bouts initiated (Fig. 1L-P). Closed-loop photoinhibition of periLC^VGLUT2^→VTA axon projections in the absence of food was rewarding and resulted in place preference (fig. S3, C, and D). To test whether the timing of activity modulation in periLC^VGLUT2^→VTA axon projections was important for influencing food intake, we performed open-loop photoinhibition of the VTA projections yoked to the same photoinhibition statistics as closed-loop experiments but noncontingent to licking behavior (see Methods “noncontingent optogenetics” and fig. S3B). Noncontingent photoinhibition did not affect consumption bout duration or bout initiation (fig. S3, E to H). Opposite to photoinhibition, closed-loop lick-triggered photostimulation (*Vglut2-IRES-Cre*, AAV-FLEX-ChR2-EGFP) of periLC^VGLUT2^→VTA axon projections suppressed food intake by shortening bout duration and reducing bout number (Fig. 1, Q to V).

### periLC^VGLUT2^ neurons negatively regulate VTA^DA^ signaling

We investigated the circuit interactions of periLC^VGLUT2^ neurons with the VTA using anterograde neurotracing. We used intersectional anatomical tracing in *Vglut2-IRES-Flpo* mice by injecting a Flp-dependent anterograde transsynaptic tracer, WGA-Cre (44, 45) (AAV-fDIO-mCherry-IRES-WGA-Cre), in the periLC and Cre-dependent AAV-FLEX-GFP in the VTA (fig. S4A). Labeled neurons downstream of periLC^VGLUT2^ projections to VTA were primarily VGAT-expressing, with a lower proportion expressing tyrosine hydroxylase (TH), a molecular marker for VTA^DA^ neurons (fig. S4, B to E), indicating that periLC^VGLUT2^ neurons may exert effects on consumption through VTA^VGAT^ neurons and VTA^DA^ neurons.

**Fig. 2.**
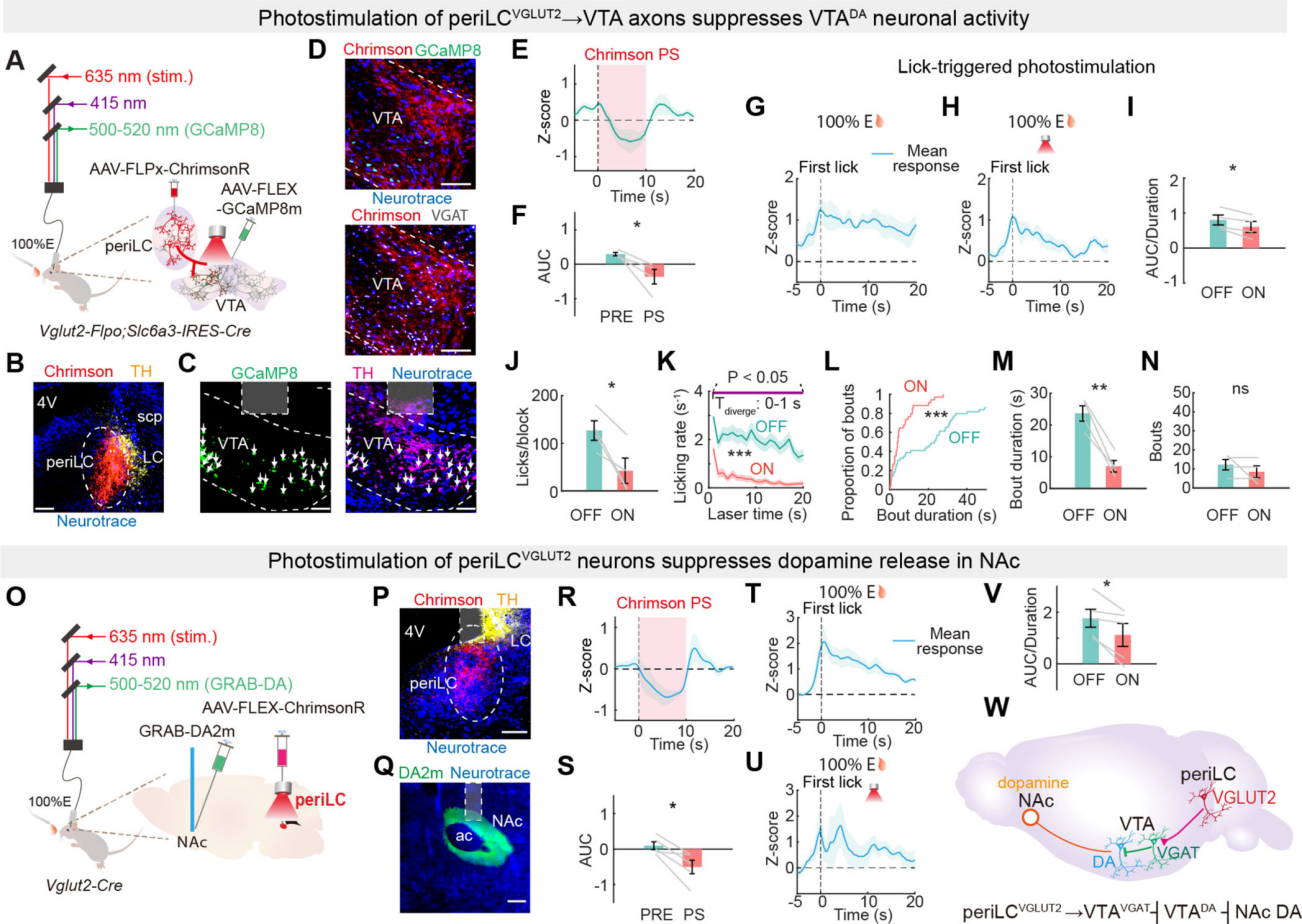
periLC^VGLUT2^ neurons suppress VTA^DA^ neuron activity and NAc dopamine. (A) Photometry and strategy for expression of Chrimson in periLC^VGLUT2^ neurons and GCaMP8m in VTA^DA^ neurons in *Vglut2-Flpo*; *Slc6a3-IRES-Cre* mice. (B, C) Expression of Chrimson-tdTomato (red) and anti-tyrosine hydroxylase immunoreactivity (TH, yellow) in periLC and LC, respectively (B). GCaMP8m (green, left), and its overlay (right) with TH (magenta) in VTA^DA^ neurons, and optical fiber track (grey) (C). Arrows indicate GCaMP8m+/TH+ neurons. Scale bars, 200 and 100 μm. (D) Expression of Chrimson (red) with GCaMP8m (green, top) or VGAT (white, bottom) in VTA. Scale bar, 100 μm. (E) VTA^DA^ neuron calcium dynamics during photostimulation (PS) of periLC^VGLUT2^→VTA axons. (F) AUC from VTA^DA^ GCaMP8m decreases during PS (paired t-test, n = 4 mice). (G-I) GCaMP8m mean responses during consumption of 100% Ensure (G) and 100% Ensure with lick-contingent PS of periLC^VGLUT2^→VTA axons (H) show decreased bout duration-normalized GCAMP8m response (I, paired t-test, n = 4 mice). (J-N) Lick-contingent PS of periLC^VGLUT2^→VTA axons decreases consumption (J, K), and bout duration (L, M) but not bout number (N) during laser-ON blocks (negative binomial generalized linear mixed models, KS-test, and paired t-test, n = 4). (O) Photometry setup and viral transduction strategy for Cre-dependent expression of Chrimson in periLC^VGLUT2^ neurons and expression of GRAB-DA2m in NAc (*Vglut2-IRES-Cre* mice). (P, Q) Expression of Chrimson (red) adjacent to TH (yellow) in the periLC and LC, respectively (P) and GRAB-DA2m (green) in NAc (Q), Scale bars, 200 μm. (R) Z-scored GRAB-DA2m fluorescence during PS of periLC^VGLUT2^ neurons. (S) AUC of GRAB-DA2m fluorescence decreases during PS. (T-V) GRAB-DA mean responses during consumption of 100% Ensure (T) and 100% Ensure with lick-contingent PS of periLC^VGLUT2^ neurons (U) show decreased bout duration-normalized NAc dopamine (V, paired t-test, n = 5 mice). (W) Circuit diagram. Data are represented as mean ± SEM. ns p>0.05, *p<0.05, **p<0.01, ***p<0.001. Statistical details are provided in Table S1.

Because VTA^VGAT^ neurons inhibit VTA^DA^ neurons (46, 47), we tested the functional effects of periLC^VGLUT2^ neuron activation on VTA^DA^ neuron activity. We used optogenetics and fiber photometry in dual transgenic mice (*Vglut2-IRES-Flpo; Slc6a3-IRES-Cre)* transduced with the Flp-dependent optogenetic activator Chrimson (48) (AAV-fDIO-Chrimson-tdTomato) in the periLC, as well as the calcium sensor GCaMP8m (49) as a proxy for neuronal activity (AAV-FLEX-GCaMP8m) in VTA^DA^ neurons, with an optical fiber over the VTA (Fig. 2, A to C). We found the periLC^VGLUT2^ axon projections overlapped regions of the VTA containing TH-expressing dopamine neurons as well as VGAT-expressing inhibitory neurons (Fig. 2D). Photostimulation of periLC^VGLUT2^→VTA axons inhibited VTA^DA^ neuron calcium dynamics (Fig. 2, E and F), which was not observed in control mice with VTA^DA^ neurons expressing GFP receiving light pulses in VTA (fig. S5). PeriLC^VGLUT2^→VTA axon photostimulation also reduced VTA^DA^ neuron calcium dynamics and food intake during 100% Ensure consumption (Fig. 2, G to J), which was evident within 1-s (Fig. 2K). Bout duration was decreased by periLC^VGLUT2^→VTA photostimulation, but the bout number was not significantly different (Fig. 2, L to N).

We also measured the effect of periLC^VGLUT2^ neuron photostimulation on dopamine in the nucleus accumbens (NAc), a major output region for VTA^DA^ neurons. We used optogenetics and fiber photometry in *Vglut2-IRES-Cre* mice transduced with Cre-dependent Chrimson (AAV-FLEX-Chrimson-tdTomato) in the periLC and the dopamine sensor, GRAB-DA2m (50), as a proxy for dopamine (AAV-GRAB-DA2m) in NAc, as well as optical fibers over the periLC and NAc (Fig. 2, O to Q, and fig. S6). Photostimulation of periLC^VGLUT2^ neurons led to decreased NAc dopamine (Fig. 2, R and S), which was not observed in control mice receiving light pulses but lacking Chrimson (fig. S6, A to E). Mice expressing a mutated negative control sensor, GRAB-rDA-mut (50), also lacked statistically significant photometry dynamics during consumption (fig. S6, F to J). PeriLC^VGLUT2^ neuron photostimulation reduced NAc dopamine dynamics during consumption (Fig. 2, T to V). These experiments indicated a circuit, periLC^VGLUT2^→VTA^VGAT^⊣VTA^DA^⊸NAc dopamine, by which periLC^VGLUT2^ neurons negatively regulate VTA^DA^ signaling to the NAc by dopamine release (Fig. 2W).

### VTA^DA^ neuron dynamics correspond to consumption duration and palatability

**Fig. 3.**
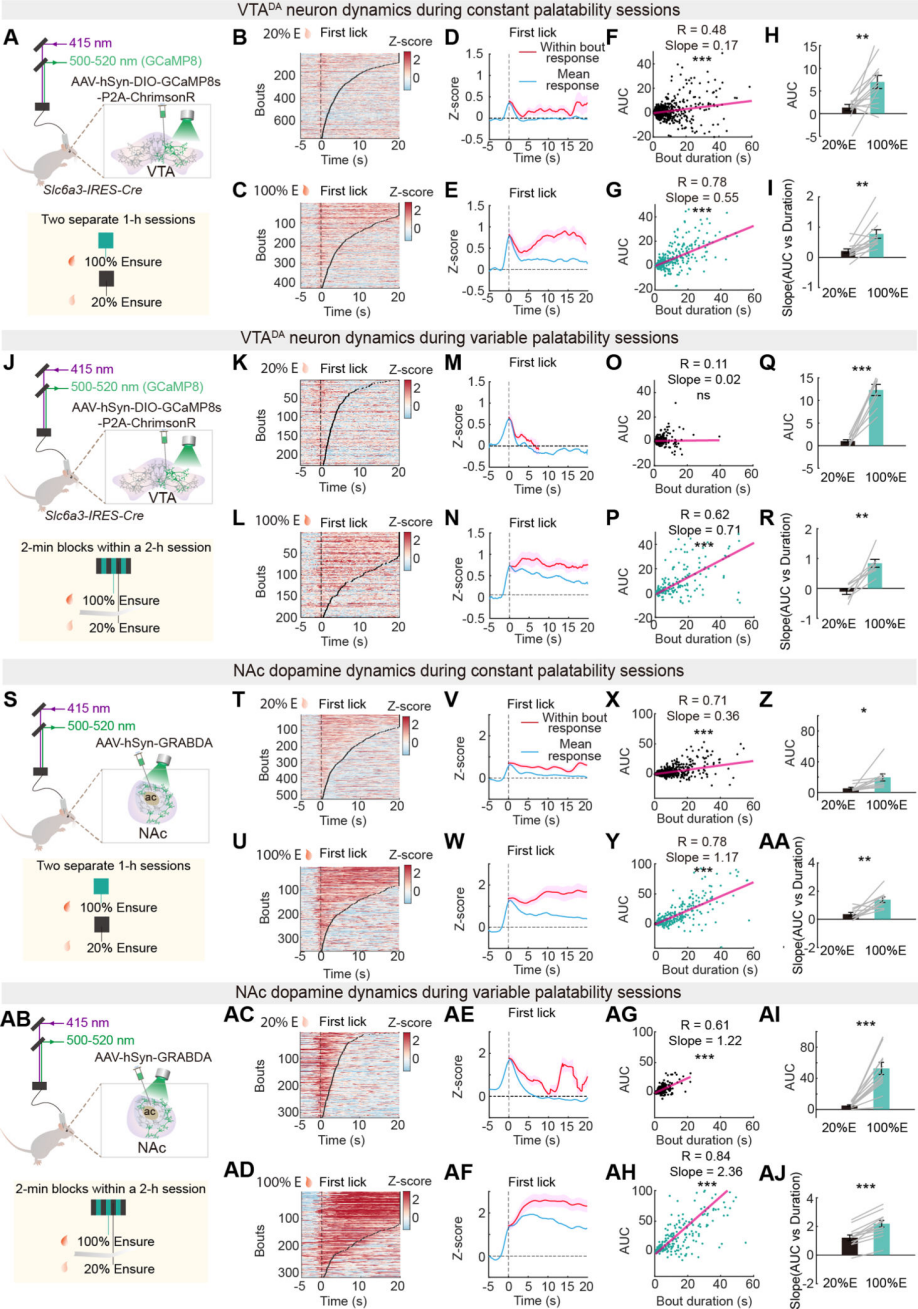
VTA^DA^ neuron activity and NAc dopamine are dependent on consumption duration and palatability. (A) Photometry of VTA^DA^ neuron calcium dynamics during consumption of 20% or 100% Ensure in separate sessions (*Slc6a3-IRES-Cre* mice). (B-E) GCaMP8s activity during consumption of 20% (B, D) and 100% Ensure (C, E). (D, E) GCaMP8s mean responses (blue) during consumption of 20% Ensure and 100% Ensure. The magenta line is the variable-length time mean response, which takes variable bout duration into account (see Methods). (F-G) Regression of GCaMP8s AUC with bout duration for consumption of 20% Ensure (F) and 100% Ensure (G) (n = 13 mice). (H-I) GCaMP8s AUC (H) slope (I) (paired t-test, n = 13 mice). (J) Setup for consumption of 20% or 100% Ensure during the same session (*Slc6a3-IRES-Cre* mice). (K-N) GCaMP8s during consumption of 20% (K, M) and 100% Ensure (L, N). (M, N) GCaMP8s mean responses (blue) and variable-length time mean response (magenta) during consumption of 20% Ensure and 100% Ensure. (O-P) Regression of GCaMP8s AUC with bout duration for 20% Ensure (O) and 100% of Ensure (P) (n = 8 mice). (Q-R) GCaMP8s AUC (Q) and slope (R) (paired t-test, n = 8 mice). (S) Photometry setup to measure NAc dopamine during consumption of 20% or 100% Ensure in separate sessions. (T-W) GRAB-DA responses during consumption of 20% (T, V) and 100% Ensure (U, W). (V, W) GRAB-DA mean responses (blue) and variable-length time mean response (magenta) during consumption of 20% Ensure and 100% Ensure. (X-Y) Regression of GRAB-DA AUC with bout duration during consumption of 20% Ensure (X) and 100% Ensure (Y) (n = 9 mice). (Z-AA) GRAB-DA AUC (Z) and slope (AA) (paired t-test, n = 9 mice). (AB) Photometry for GRAB-DA during consumption of 20% or 100% Ensure in the same session. (AC-AF) GRAB-DA responses during consumption of 20% (AC, AE) and 100% Ensure (AD, AF). (AE, AF) GRAB-DA mean responses (blue) and variable-length time mean response (magenta) during consumption of 20% Ensure and 100% Ensure. (AG-AH) Regression of GRAB-DA AUC with bout duration during consumption of 20% Ensure (AG) and 100% of Ensure (AH) (n = 13 mice). (AI-AJ) GRAB-DA AUC (AI) and slope (AJ) (paired t-test, n = 13 mice). Data are represented as mean ± SEM. ns p>0.05, **p<0.01, ***p<0.001. Statistical details are in Table S1.

Next, we tested whether palatability and consumption bout duration were associated with different levels of VTA^DA^ neuron activity. We used fiber photometry to measure VTA^DA^ neuron calcium dynamics during separate experimental sessions delivering either higher or lower palatability Ensure (100% or 20% Ensure, respectively) in *Slc6a3-IRES-Cre* mice that were transduced with the genetically encoded calcium indicator GCaMP8s and the red-shifted opsin, Chrimson (AAV-DIO-jGCaMP8s-P2A-Chrimson) (51) (Fig. 3A). VTA^DA^ neurons responded prior to licking and showed a peak mean response at initial reward delivery (Fig. 3, B to E). However, in this self-paced feeding task, mice could continue the lick bout to continuously receive more liquid food. The VTA^DA^ neuron responses persisted throughout the consumption bouts (Fig. 3, D, and E). This aspect of VTA^DA^ neuron activity during ongoing food consumption has not been examined, but it was similar to the timescale of palatability-sensitive periLC^VGLUT2^ neuron activity, thus we investigated this further.

The integrated VTA^DA^ neuron response magnitude increased with bout duration (Fig. 3, F, and G), which is consistent with the persistence of VTA^DA^ neuron activity throughout an eating bout. This relationship had a higher slope for more palatable food (Fig. 3, H and I), showing that the persistent VTA^DA^ neuron response during consumption is scaled by palatability. We reproduced this result in *Slc6a3-IRES-Cre* mice that were transduced with only GCaMP8m (fig. S7, A to G). Mice expressing only EGFP lacked statistically significant photometry dynamics during consumption (fig. S7, H to K). We also noted that VTA^DA^ neuron responses during food intake decreased modestly during a 2-h session, especially with higher-palatability food (fig. S8, A to D), likely due to increasing satiety from greater calorie intake (fig. S8E). An aversive visceral state induced by LiCl injection also reduced the VTA^DA^ neuron response during consumption of 100% Ensure (fig. S9). In contrast, hunger elicited by food-restriction to 85% of starting body weight increased the VTA^DA^ neuron response during consumption of 20% Ensure (fig. S10).

Food palatability responses are sensitive to absolute differences in nutrient and flavor content, but acute comparisons of food palatability reveal stronger contrasts that are not apparent when assessing palatability separately, which is called hedonic contrast (52, 53). We used fiber photometry to compare VTA^DA^ neuron calcium dynamics when consumption switched between 100% or 20% of Ensure during the same session (variable palatability session, Fig. 3J). Unlike constant palatability sessions, mice in variable palatability sessions showed significantly longer bout durations during consumption of 100% Ensure than during consumption of 20% Ensure (fig. S11A). VTA^DA^ neuron calcium response area under the curve (AUC) increased during consumption of higher palatability food, showing a predictive relationship to bout duration (Fig. 3, K to R). However, during the consumption of lower palatability food, VTA^DA^ neuron calcium responses and bout duration were diminished and lacked a predictive relationship (Fig. 3, K to R, and fig. S11B). Reducing palatability by adulterating 100% Ensure with quinine also reduced the overall VTA^DA^ neuron response during consumption and the slope of this response (fig. S12).

We also monitored NAc dopamine during higher and lower palatability food consumption in mice expressing GRAB-DA in the NAc. NAc dopamine was elevated during consumption, and this was scaled by food palatability in both constant and variable palatability conditions (Fig. 3, S to AJ), similar to VTA^DA^ neuron calcium responses.

### VTA^DA^ neuron activation increases palatable food consumption

**Fig. 4.**
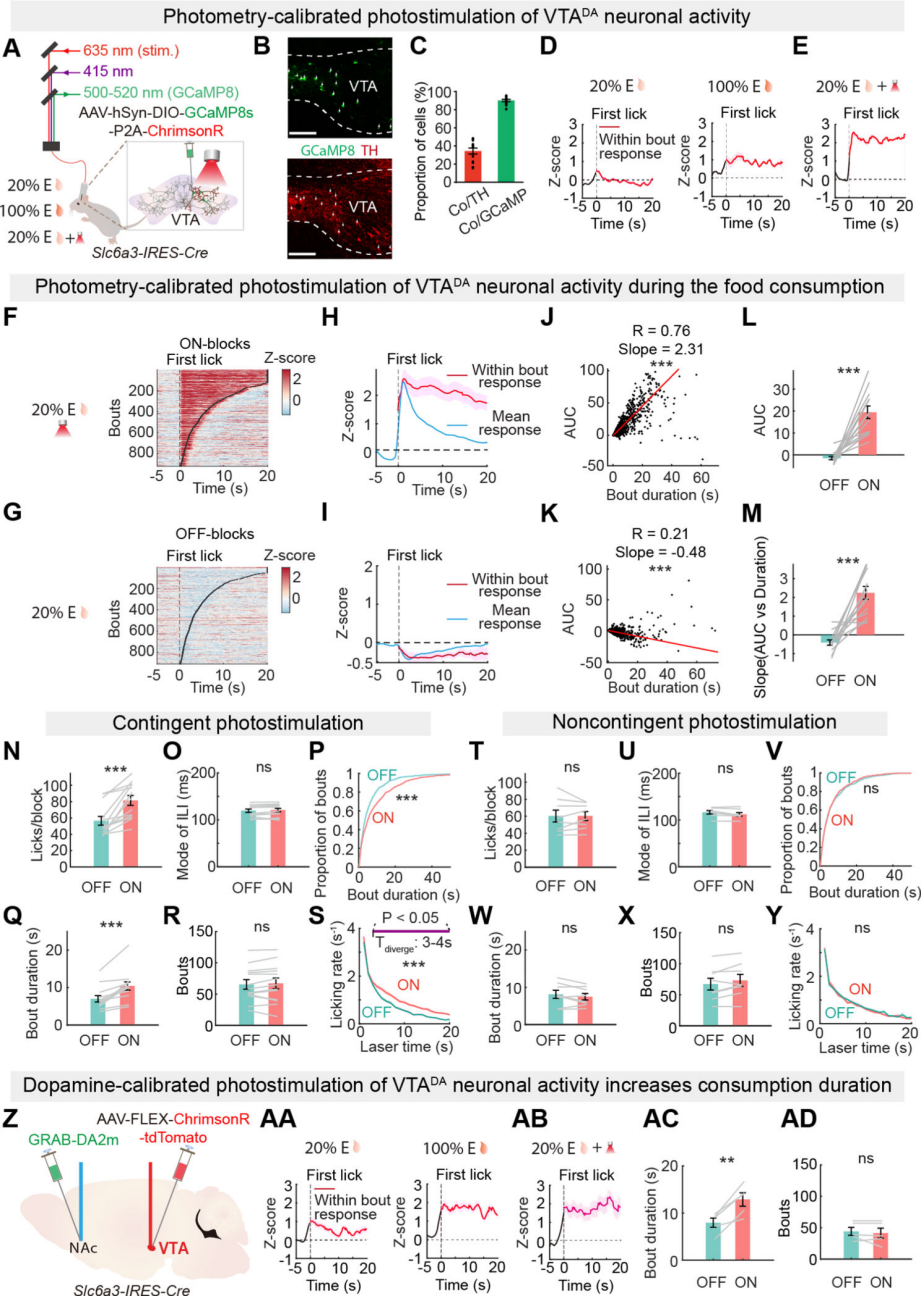
VTA^DA^ neurons promote food consumption duration and palatability. (A) Photometry-calibrated photostimulation (PS) setup. E: Ensure. (B) Expression of GCaMP8s (green, top) and overlay with anti-TH (red, bottom) within VTA. Arrows indicate GCaMP8s+ TH+ neurons. Scale bar, 100 μm. (C) Co-expression among GCaMP8s (left) and anti-TH cells (right) within the VTA. (D, E) Recorded VTA^DA^ neuron activity during 20% Ensure (D, left), 100% Ensure (D, right), and 20% Ensure with photometry-calibrated VTA^DA^ neuron PS (E). Vertical line: First lick of a bout. (F-I) GCaMP8s responses during consumption of 20% Ensure with photometry-calibrated VTA^DA^ neuron PS in ON-blocks (F, H) and 20% Ensure in OFF-blocks (G, I). (H, I) GCaMP8s mean responses (blue) and mean response within a bout (magenta) during consumption of 20% Ensure with photometry-calibrated VTA^DA^ neuron PS (H) and 20% Ensure (I). (J-K) Regression of GCaMP8s AUC with bout duration during consumption of 20% Ensure with photometry-calibrated VTA^DA^ neuron PS (J) and 20% Ensure (K) (n = 13 mice). (L-M) GCaMP8s AUC (L) and slope (M) for photometry-calibrated VTA^DA^ neuron PS (paired t-test, n = 13 mice). (N-S) Lick-contingent photometry-calibrated PS of VTA^DA^ neurons increases consumption (N), and bout duration but not bout number during laser-on blocks (O-R), which is apparent after 3-s of PS onset (S) (negative binomial generalized linear mixed model, KS-test and paired t-test, n = 13 mice). (T-Y) Noncontingent PS does not affect licking behaviors (T), nor bout duration and bout number (U-Y) (negative binomial generalized linear mixed model, KS-test and paired t-test, n = 8 mice). (Z) Dopamine-calibrated photometry and PS setup. (AA-AB) NAc dopamine responses during 20% Ensure (AA, left), 100% Ensure (AA, right), and 20% Ensure with photometry-calibrated VTA^DA^ neuron PS (AB). (AC-AD) Lick-contingent photometry-calibrated PS of VTA^DA^ neurons increases bout duration (AC) but not bout number (AD) during laser-ON blocks (paired t-test, n = 5 mice). Data are represented as mean ± SEM. ns p>0.05, **p<0.01. ***p<0.001. Statistical details are in Table S1.

Next, we investigated a causal relationship between VTA^DA^ neuron activity and consumption bout duration. We tested whether boosting VTA^DA^ neuron activity with lick-contingent photostimulation during consumption of lower palatability food would increase bout duration relative to interleaved blocks in which licking delivered the same lower palatability food but no laser stimulation (Fig. 4A). Co-expressing GCaMP8s and Chrimson (48) in VTA^DA^ neurons (Fig. 4, B and C) allowed us to activate the neurons with red light (635 nm) while simultaneously monitoring calcium dynamics. Dopamine neuromodulation is complex and sensitive to the dynamics and level of VTA^DA^ neuron activity (54, 55), so we calibrated the perturbation during 20% Ensure consumption to mimic an overall level of VTA^DA^ neuron activity associated with consuming 100% Ensure while taking into account the partial transduction of neurons with the transgenes (less than 50% in pilot experiments). We used photometry-calibrated, transduction-normalized photostimulation of VTA^DA^ neurons (55) during consumption of 20% Ensure to twice the level (accounting for partial transduction penetrance of VTA^DA^ neurons) associated with consumption of high palatability 100% Ensure in the absence of photostimulation (Fig. 4, B to E). *Post hoc* analysis of the experimental subjects showed that 34% ± 12% of TH-positive neurons co-expressed the transgene (Fig. 4C), indicating that this was not a supraphysiological perturbation.

Closed-loop, lick-triggered calibrated photostimulation of VTA^DA^ neurons in alternating 2-min ON and OFF blocks produced neural dynamics during consumption that were similar to consumption of 100% Ensure in variable palatability sessions (hedonic contrast), even though only 20% Ensure was delivered throughout the sessions (Fig. 4, D and E). The calibrated laser-ON blocks during 20% Ensure consumption led to elevated VTA^DA^ neuron activity, but the laser-OFF blocks showed a negative response during consumption (Fig. 4, F to M).

Photometry-calibrated VTA^DA^ neuron activation using closed-loop lick-contingent photostimulation prolonged bout duration without affecting bout initiation (Fig. 4, N to R). The effect of VTA^DA^ neuron activation primarily affected longer bouts, showing a statistically significant increase in consumption after 3-s (Fig. 4S). Neither contingent (Fig. 4O) nor noncontingent (Fig. 4U) photostimulation of VTA^DA^ neurons changed the fundamental lick oscillator interval (56). In addition, neither lick-noncontingent photostimulation (Fig. 4, T to Y, fig. S3B) nor lick-contingent light pulses in GCaMP8m or GFP control mice (both lacking Chrimson) (fig. S13, A to D) increased consumption duration or bout number. Moreover, photometry-calibrated lick-contingent photostimulation did not increase bout duration across successive feeding bouts (fig. S13, E to H), although calorie intake was significantly higher for photostimulation-ON blocks (fig. S13I).

Lick-contingent photostimulation of VTA^DA^ neurons with supraphysiological activation increased VTA^DA^ neuron activity during the laser-ON blocks, but the laser-OFF blocks also showed a large negative response (fig. S14, A to H). This perturbation also significantly increased consumption and bout duration but not bout number (fig. S14, I to N). Even with stronger VTA^DA^ neuron photostimulation, the consumption duration did not progressively increase across the lick-contingent photostimulation session (fig. S14, O to S). Notably, for high laser power lick-contingent photostimulation of VTA^DA^ neurons, the mean rate of food intake across a session became diminished in the laser-OFF blocks, which was not observed for the laser-ON blocks or either set of blocks in the photometry-calibrated experiments (fig. S14, T to Y).

As an alternative calibration approach, we monitored NAc dopamine with GRAB-DA during consumption and with VTA^DA^ neuron photostimulation. We used *Slc6a3-IRES-Cre* mice to transduce VTA^DA^ neurons with Chrimson and expressed GRAB-DA2m in NAc neurons (Fig. 4Z and fig. S15, A to C), placing optical fibers over VTA and NAc. We used NAc dopamine release to calibrate VTA^DA^ neuron photostimulation during consumption of 20% Ensure to the level of NAc dopamine associated with 100% Ensure consumption (Fig. 4, AA and AB). NAc dopamine release-calibrated VTA^DA^ neuron activation using closed-loop lick-contingent photostimulation prolonged bout duration without affecting bout initiation (Fig. 4, AC and AD, and fig. S15, D to K). Lick-contingent photostimulation of VTA^DA^ neurons with supraphysiological activation in these mice also increased consumption and bout duration but not bout number (fig. S16).

### VTA^DA^ neuron inhibition reduces palatable food consumption

**Fig. 5.**
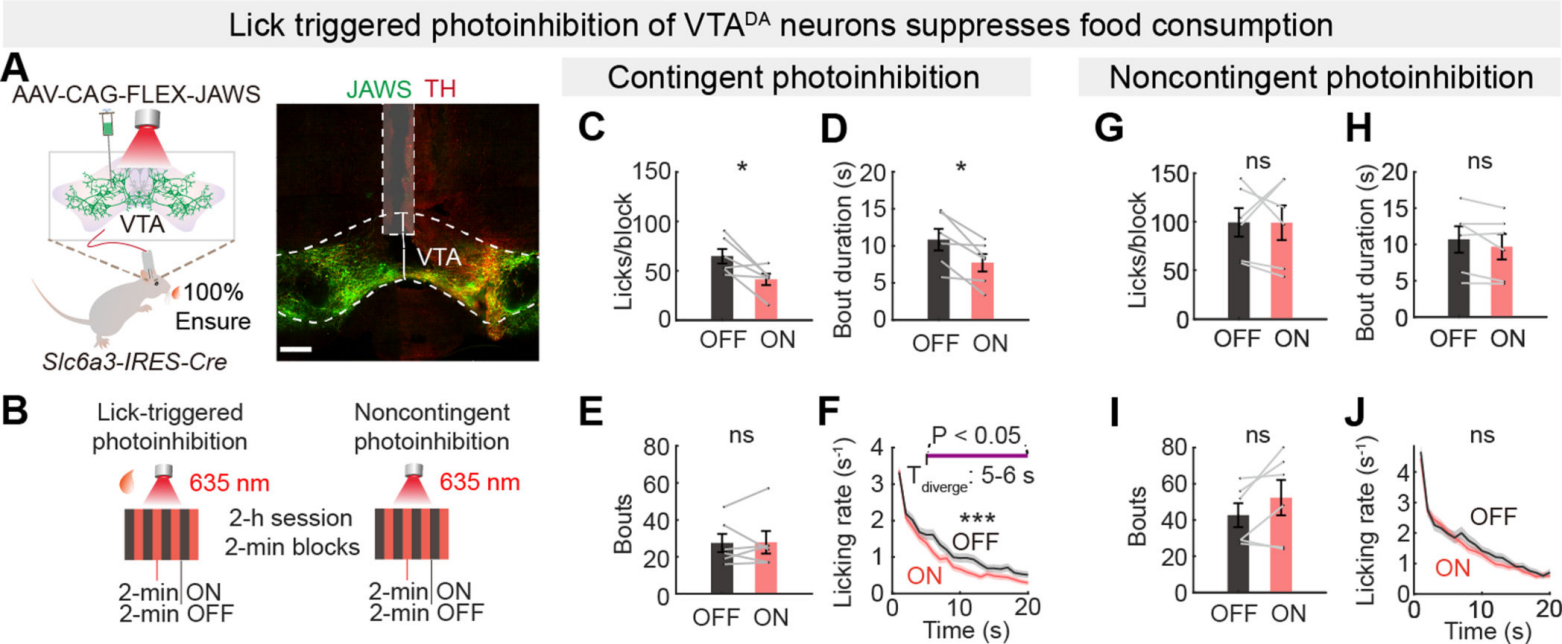
VTA^DA^ neurons are necessary for prolonged food consumption duration. (A) Left panel, setup for photoinhibition of VTA^DA^ neurons using AAV-CAG-FLEX-JAWS-GFP in mice consuming 100% Ensure (*Slc6a3-IRES-Cre* mice). Right panel, the viral expression of FLEX-JAWS within VTA (green), the fiber tract, and the overlayed histological images with Anti-TH (red). Scale bar, 200 μm. (B) Schematic for lick-contingent or noncontingent photoinhibition (2 min ON or OFF blocks). (C-F) Lick-contingent photoinhibition of VTA^DA^ neurons reduced licking behaviors (C) and bout duration but not bout number (D-E), which is apparent after 5-s of PS onset (F) during laser-ON blocks (negative binomial generalized linear mixed model, KS-test and paired t-test, n = 6 mice). (G-J) Noncontingent photoinhibition did not significantly affect licking behaviors (G), bout duration, or bout number (H-J) (negative binomial generalized linear mixed model, KS-test and paired t-test, n = 6 mice). Data are represented as mean ± SEM. ns p>0.05, *p<0.05, ***p<0.001. Statistical details are in Table S1.

We tested whether lick-triggered inhibition of VTA^DA^ neurons during palatable Ensure consumption would have similar effects as reducing palatability to suppress food intake and consumption bout duration (Fig. 5A). We used photoinhibition in VTA^DA^ neurons expressing the red-shifted inhibitory opsin, JAWS (57), during alternating block-wise (laser-ON/laser-OFF) lick-contingent or yoked noncontingent photoinhibition protocols (Fig. 5B). Lick-contingent photoinhibition reduced food consumption and bout duration but did not affect the number of bouts (Fig. 5, C to E). The effect of VTA^DA^ neuron activation primarily affected longer bouts, showing a statistically significant decrease in consumption after 5-s (Fig. 5F). By contrast, noncontingent photoinhibition did not significantly change consumption, bout duration, or bout number (Fig. 5, G to J).

Because VTA^DA^ neurons affect behavioral reinforcement, we examined whether the feeding microstructure was different in the first or second halves of the photoinhibition sessions. Lick-triggered photoinhibition of VTA^DA^ neurons suppressed feeding bout duration similarly in the first and second halves of the session and showed no statistically significant effect on bout initiation (fig. S17, A to C). Noncontingent photoinhibition did not affect bout duration over the course of the behavioral session (fig. S17D), but there was a statistically significant reduction in bout duration during the second half of the session with noncontingent photoinhibition of VTA^DA^ neurons (fig. S17E). Neither contingent (fig. S17F) nor noncontingent (fig. S17G) photoinhibition of VTA^DA^ neurons changed the fundamental lick oscillator interval.

### Semaglutide transiently suppresses feeding and VTA^DA^ neuron responses

Anti-obesity treatment by the GLP-1R agonist semaglutide activates satiety pathways (58). We used semaglutide to explore the influence of satiety pathways on VTA^DA^ neuron regulation of hedonic feeding microstructure.

**Fig. 6.**
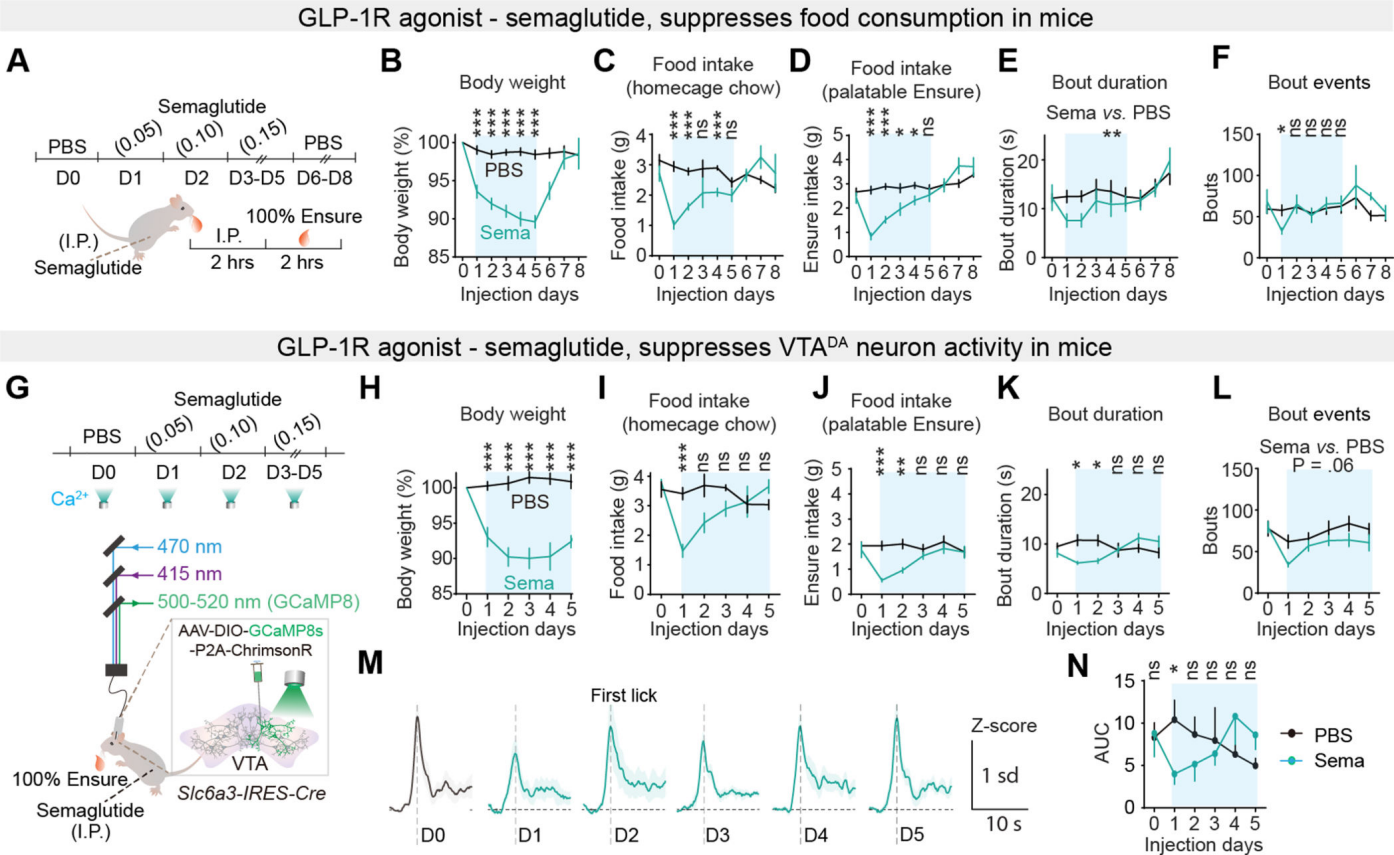
Semaglutide modulates VTA^DA^ neurons to shorten consumption bout duration. (A) Experimental design to test the effects of semaglutide (doses in mg/kg) on food consumption. (B-F) Behavioral summary of body weight (B), homecage chow food intake (C), 2-hour Ensure intake (D), bout duration (E), and bout numbers (F) during semaglutide or PBS injection (n = 8 mice). (G) Photometry setup for recording VTA^DA^ neuron activity during 100% Ensure intake with semaglutide and PBS injection (*Slc6a3-IRES-Cre* mice). (H-L) Behavioral summary of body weight (H), homecage food intake (I), 1-hour Ensure intake (J), bout duration (K), and bout numbers (L) during Semaglutide and PBS injection (n = 8 mice). (M) GCaMP8s response of VTA^DA^ neuron activity to 100% Ensure from day 0 to day 5 during Semaglutide injection (n = 8 mice). (N) Comparison of GCaMP responses to 100% Ensure in VTA^DA^ neurons on day 0 to day 5 with PBS and Semaglutide injections (rmANOVA, n = 8 mice). Data are represented as mean ± SEM. ns p>0.05, *p<0.05, **p<0.01, ***p<0.001. Statistical details are in Table S1.

To minimize the aversive effects of semaglutide, we adopted a ramped once-daily dose protocol (see Methods) (58) while monitoring the body weight of single-caged mice with *ad libitum* homecage access to chow-food (Fig. 6A). Semaglutide suppressed body weight and homecage food intake (Fig. 6, B, and C). On Day 1 of treatment, prior to weight loss, in a 2-h session with access to high palatability 100% Ensure, consumption was suppressed due to reduced bout duration and a lower number of bouts initiated (Fig. 6, D to F, and fig. S18). Interestingly, palatable food intake and bout duration increased during the dose escalation, eventually returning to control levels at the highest doses of semaglutide (Fig. 6, D to F and, fig. S18).

To investigate the influence of semaglutide on VTA^DA^ neuron consummatory responses, we performed the same protocol while monitoring VTA^DA^ neuron calcium dynamics in *Slc6a3-IRES-Cre* mice co-expressing GCaMP8s and Chrimson (Fig. 6G). As before, during semaglutide treatment, for high palatability 100% Ensure during day 1, there was decreased consumption and bout duration (Fig. 6, H to L), and VTA^DA^ neuron calcium responses were also reduced (Fig. 6, M and N). Nevertheless, during dose escalation of semaglutide, Ensure consumption and bout duration increased to pre-semaglutide treatment levels, and VTA^DA^ neural dynamics showed a corresponding increase (Fig. 6, M, and N).

### VTA^DA^ neurons bidirectionally control palatable food consumption during semaglutide treatment

**Fig. 7.**
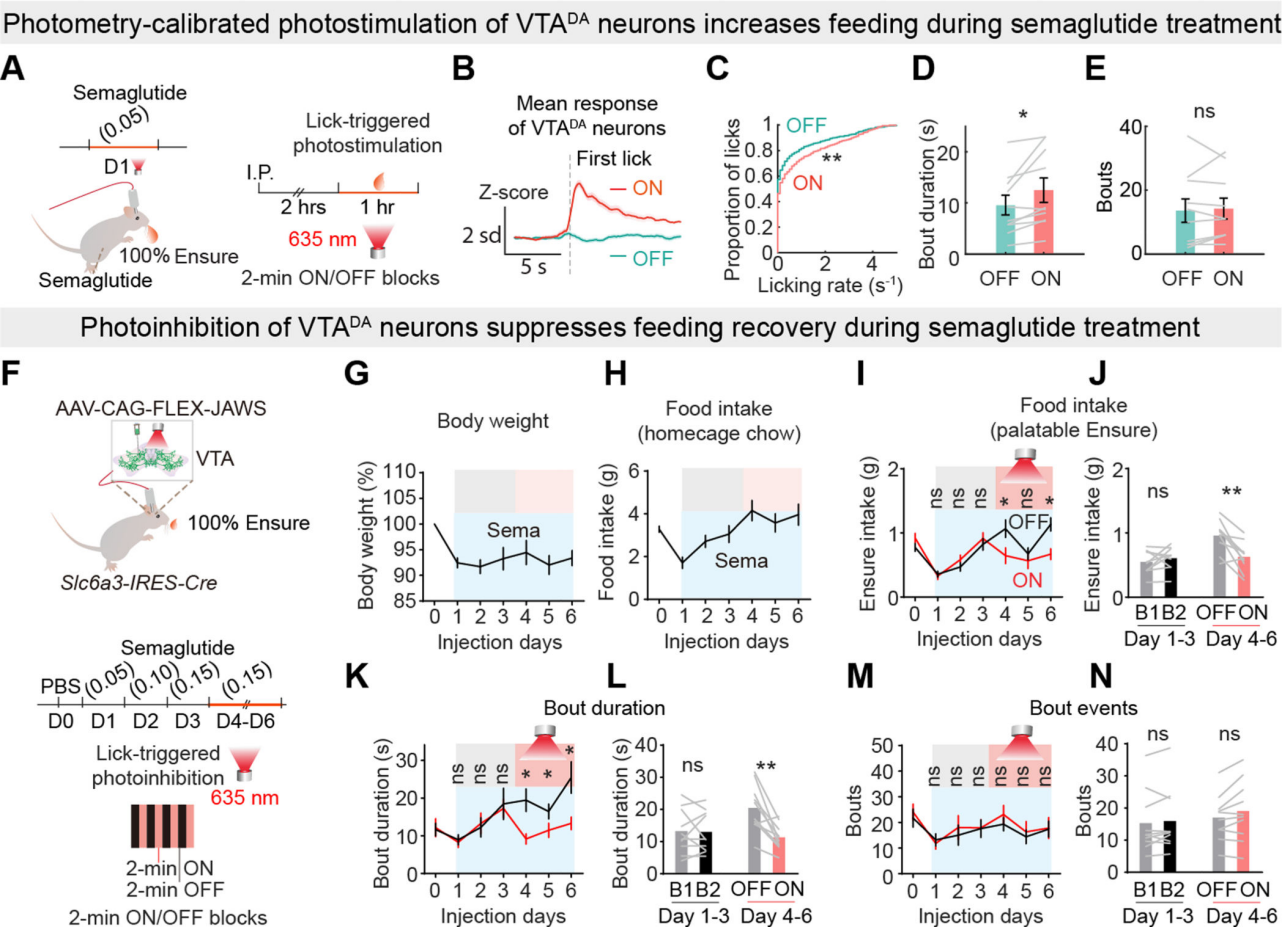
VTA^DA^ neurons bidirectionally control palatable food consumption with semaglutide treatment. (A) Dual photometry and photostimulation for recording and activating VTA^DA^ neuron activity during food consumption with Semaglutide injection. (B) GCaMP response of VTA^DA^ neuron activity to 100% Ensure during photostimulation of VTA^DA^ neurons following semaglutide injection on Day 1. (C-E) Lick-contingent photostimulation of VTA^DA^ neurons increased lick numbers (C) and bout duration (D) but not bout number (E) in laser-ON blocks (KS-test and paired t-test, n = 10 mice). (F) Experimental design for photoinhibition of VTA^DA^ neurons during day 4 to day 6 with the highest dose of semaglutide. (G-H) Body weight (G) and home cage chow food intake (H) during semaglutide treatment. (I-N) Reduced Ensure intake (I, J), and bout duration (K, L) but not bout numbers (M, N) during the laser-ON blocks compared to laser-OFF blocks for Days 4–6 (n = 10 mice). Days 1–3 are analyzed to show Ensure intake, bout duration, and bout number across the same alternating 2-min blocks (B1, B2) in the absence of photoinhibition. Data are represented as mean ± SEM. ns p>0.05, *p<0.05, **p<0.01, ***p<0.001. Statistical details are in Table S1.

Because semaglutide initially reduced bout duration and VTA^DA^ neuron responses to highly palatable food, we first tested the capability of lick-triggered photostimulation to counteract the effect of semaglutide on bout duration. Photostimulation of semaglutide-treated mice on Day 1 was calibrated to increase VTA^DA^ neuron activity during 100% Ensure consumption to the level prior to semaglutide (Fig. 7A). Photometry-calibrated VTA^DA^ neuron activation prolonged bout duration using lick-contingent photostimulation without affecting bout initiation in the semaglutide-treated mice (Fig. 7, B to E).

Next, we examined the role of VTA^DA^ neurons during the recovery of highly palatable food intake following semaglutide dose escalation in *Slc6a3-IRES-Cre* mice expressing JAWS in VTA^DA^ neurons (Fig. 7F). Semaglutide reduced body weight during the course of treatment (Fig. 7G), and homecage chow-food intake was transiently decreased (Fig. 7H). Semaglutide suppressed bout duration for 100% Ensure on treatment Day 1. On Day 2 and Day 3, Ensure intake and bout duration increased compared to day 1 (Fig. 7I). Because there were no laser pulses on these days, analysis of Ensure intake and bout duration showed no significant differences when the session was analyzed in alternating 2-min blocks on Days 1–3 (Fig. 7, I to N). From Day 4 to Day 6, mice with higher dose semaglutide treatment underwent lick contingent photoinhibition (Fig. 7, I to N). Unlike in the laser-OFF period, which showed recovery of mean Ensure intake and bout duration, lick-triggered inhibition of VTA^DA^ neuron calcium responses in the same sessions suppressed consumption of more palatable food as well as bout duration (Fig. 7, I to N). For mice expressing the control virus, the feeding bout duration in the laser-ON and laser-OFF periods was not significantly different (fig. S19).

## Discussion

We found that hedonic eating was controlled by VTA^DA^ neurons, which sustained palatable food consumption. VTA^DA^ neuron activity during consumption was scaled by palatability and corresponded to the duration of food consumption bouts. Correspondingly, increasing VTA^DA^ neuron activity selectively during consumption was sufficient to increase food intake and bout duration. Suppression of VTA^DA^ neurons solely during consumption reduced food intake and bout duration without influencing bout initiation. This role of VTA^DA^ neurons was transiently suppressed by engaging satiety pathways with semaglutide. Conversely, the increase in palatable food consumption after weight loss during chronic semaglutide treatment was reversed by closed-loop inhibition of VTA^DA^ neurons. Thus, dynamic regulation of VTA^DA^ neurons during the consummatory phase of hedonic eating underlies a physiologically relevant role in promoting the consumption of palatable food once ingestion is initiated and opposes the appetite-reducing effects of semaglutide.

**Fig. 8.**
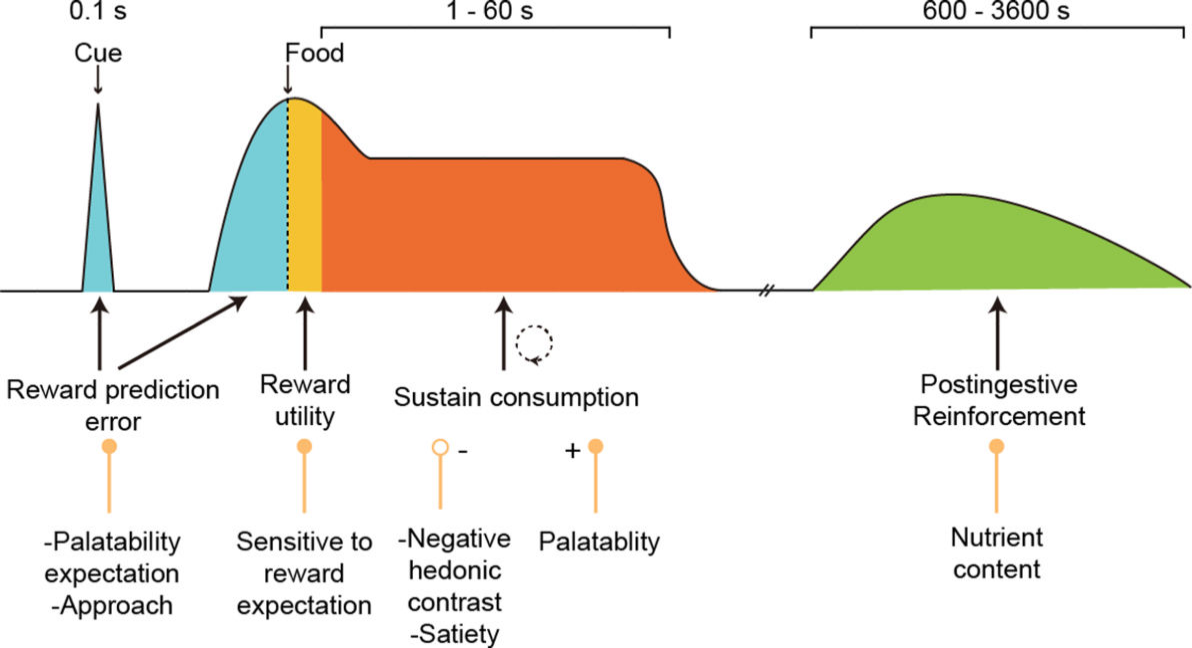
Summary of VTA^DA^ neuron activity during feeding behaviors. VTA^DA^ neuron activity in different phases of food intake.

Our findings complement prior investigations of VTA^DA^ neuron functions, which show temporally distinct responses at different phases of eating behavior (Fig. 8). First, reward prediction occurs in response to cues that signal food availability and lead to a conditioned approach (15, 25–27, 59). Second, the response during reward ingestion has been attributed to reward utility that is not fully predicted by a cue, and this diminishes or disappears in well-learned cued tasks that deliver small reward amounts (15, 60). Last, long-timescale reinforcement occurs by post-ingestive mechanisms (10, 28). Our findings demonstrated that VTA^DA^ neurons signal food palatability at intermediate timescales and sustain hedonic eating. A causal function of VTA^DA^ neurons during consumption has been difficult to isolate previously due to the use of open-loop perturbation methods that were not restricted to natural VTA^DA^ neuron dynamics (19–21, 61).

Palatability affects both the positive experience of eating (hedonics) and promotes further consumption. Prior work, including in humans, has indicated the involvement of several brain areas in the perception of pleasure and value associated with palatable food (62–64). Although hedonic perception is subjective, and thus is difficult to evaluate in animals, motor actions are elicited as a hedonic response (65). Here, we investigated neural circuit mechanisms mediating the objective motor effects of palatability on hedonic eating by using behavioral methods compatible with real-time neural dynamics measurements and manipulations during palatable food consumption. We found that VTA^DA^ neurons are modulated by palatable food intake to increase consumption bout duration. Moreover, the effect of food-restriction to increase the response of VTA^DA^ neurons to less palatable food indicates that this consummatory mechanism is shared in hunger-driven and hedonic food intake (66, 67). Additional studies are needed to investigate the effects of VTA^DA^ neuron perturbation on the subjective effects of palatable food consumption.

Palatability in humans is sensitive to both absolute hedonic differences and hedonic contrast for food sources (52, 53). In mice, we found that the response of VTA^DA^ neurons during ingestion reflects these aspects of palatability (fig. S20). Lower palatability food was consumed with similar bout durations as higher palatability food when provided in separate constant palatability sessions, although VTA^DA^ neurons showed stronger responses to highly palatable food. However, for hedonic contrast, the consumption amount and bout duration were greatly enhanced for the higher palatability food, whereas the lower palatability food showed lower bout duration and negative VTA^DA^ neuron responses. Hedonic contrast was recapitulated with lick-contingent VTA^DA^ neuron photostimulation. Moreover, the negative response of VTA^DA^ neurons during negative hedonic contrast demonstrated that elevated VTA^DA^ neuron activity was not necessary to produce consummatory behavior. Instead, VTA^DA^ neurons are necessary to sustain consumption for longer bouts associated with higher palatability food. Low VTA^DA^ neuron activity resulted in short consumption bouts. We found that these factors prolong consummatory bouts on a timescale greater than 1 to 3-s (Fig. 3S, and fig. S2, H and K). This modulatory effect of VTA^DA^ neurons to sustain consumption is consistent with increasing evidence of a dichotomy between the reward-processing and learning effects of VTA^DA^ neurons and the invigoration of motor actions (16, 55, 68–73).

The effects of VTA^DA^ neurons on motor vigor fit into consumption-control circuits uncovered by previous studies. The nucleus accumbens (NAc), a primary target of VTA^DA^ neurons, controls consumption bout duration by pausing the firing of NAc GABA-releasing projections to LH^GABA^ neurons (74), which are disinhibited (74), eliciting consummatory motor responses even towards non-food objects (75–77). This indicates a connection between palatability coding by VTA^DA^ neurons and the regulation of consummatory motor outputs. This is a mechanism for the objective effects of palatability to sustain bout duration. Although the taste of food can elicit licking in forebrain transected rodents (78, 79), VTA^DA^ neuron responses and their downstream circuit elements were scaled by food palatability and modulated this process to sustain longer consumption bouts. Low or negative VTA^DA^ neuron activity during consumption increases the probability that licking will stop, which terminates the positive feedback cycle between taste on the motor actions of licking that maintains a consumption bout.

PeriLC^VGLUT2^ neurons project to the VTA and their activity suppresses VTA^DA^ neuron activity and NAc dopamine. This is an indirect pathway because periLC^VGLUT2^ neuron axon projections were primarily upstream of inhibitory VTA^VGAT^ neurons. Thus, our prior findings that a subset of periLC^VGLUT2^ neurons are inhibited time-locked to palatable food consumption is consistent with a disinhibitory circuit to activate VTA^DA^ neurons and NAc dopamine during consumption (Fig. 2W).

VTA^DA^ neuron responses that control the duration of palatable food consumption were increased by hunger and reduced by aversive flavors or states as well as by satiety, including the appetite-lowering effects of semaglutide. Reduced bout duration and VTA^DA^ neuron amplitudes for palatable food consumption upon initial exposure to semaglutide were similar to the effects of closed-loop consumption-triggered VTA^DA^ neuron photoinhibition. This is different from a previous report that used a 20-fold higher semaglutide dose and showed slightly increased VTA^DA^ neuron activity during food consumption (80). Chronic GLP-1R agonist therapy in human patients is associated with reduced palatable food cravings (81–84). However, consumption and palatability of sweet foods do not show statistically significant changes in humans treated with GLP-1R agonists (81, 84). Strikingly, rats show elevated consumption of sucrose solution in conjunction with weight loss during chronic semaglutide treatment (85). In addition, as weight loss becomes pronounced during GLP-1R agonist therapy, brain regions associated with palatable food intake show increased activity when presented with palatable food cues (86). This indicates that an opposing homeostatic response to GLP-1R agonist therapy may lead to progressively increased consumption of sweet food, rendering subjects vulnerable to hedonic eating. Our findings indicate that VTA^DA^ neuron responses to palatable food are necessary and sufficient for this process. Taken together, these results highlight the importance of VTA^DA^ neurons to mediate consumption behaviors, their role in semaglutide effects on palatable food intake, and the potential for modulation of these neurons to reverse the elevated consumption of sweet foods after weight loss on semaglutide.

## Materials and Methods

### Animals

All experimental protocols adhered to U.S. National Institutes of Health guidelines for animal research and were approved by the Institutional Animal Care and Use Committee (IACUC) and Institutional Biosafety Committee (IBC) at Janelia Research Campus and the University of California, San Diego. Mice were housed under a 12-hour light/dark cycle (lights on at 06:00 and off at 18:00) with *ad libitum* access to water and rodent chow (PicoLab Rodent Diet 20, 5053 tablets, TestDiet). We used both male and female adult mice (8–48 weeks old) from the following Cre recombinase-expressing lines: *Slc6a3-IRES-Cre* (Jackson Lab stock 006660) and *Vglut2-(Slc17a6)-IRES-Cre* (Jackson Lab stock 028863), *Vglut2-IRES2-FLPo-D* (Jackson Lab stock 030212)*, Slc6a3-IRES-Cre; Vglut2-IRES2-FLPo-D,* and C57BL6/J. Mice used for optogenetic perturbation or photometry experiments were singly housed to prevent interference with cranial implants by other animals.

Experimental *Slc6a3-IRES-Cre* mice that were used in optogenetic neuromodulation for food consumption (expressing AAV9-hSyn-DIO-jGCaMP8s-P2A-ChrimsonR-ST in VTA) were also used for food consumption under semaglutide treatment. The contingent and noncontingent optogenetic perturbations were performed with the same mice. There was no randomization or blinding in this study, and the sample size followed standard practices established in the field. Mice were included only when surgical targeting was confirmed accurate.

### Viral injections and stereotaxic surgery

Viruses with the following tilters and volumes were injected (rAAV serotype format): rAAV2-CAG-FLEX-rev-ArchT-GFP (2e12 GC/mL, UNC, 140 nL in periLC), rAAV2-Ef1a-FLEX-rev-ChR2-EYFP (2.6e12 GC/mL, UNC, 140 nL in periLC), AAV5-hSyn-DIO-eGFP(7.2e12 GC/mL, Addg50457, 300 nL in VTA), AAV9-hSyn-FLEX-GCaMP8m (7.2e12 GC/mL, Addg162378, 300 nL in VTA), AAV9-hSyn-DIO-jGCaMP8s-P2A-ChrimsonR-ST (8e12 GC/mL, Addg174007, 300 nL in VTA), AAV2-Syn-fDIO-mCherry-IRES-WGA-Cre (5.9e12 GC/mL, **JRC240621–2**, 100 nL in periLC), AAV5-hSyn-DIO-eGFP (1.6e13 GC/mL, Addg**50457**, 500 nL in VTA), AAV8-Syn-FLPx-rc-[ChrimsonR-tdTomato] (8e12 GC/mL, 100–300 nL in periLC), AAV5-hSyn-FLEX-rc(ChrimsonR-tdTomato] (8e12 GC/mL, Addg62723, 100–300 nL in periLC, and 500 nL in VTA), AAV9-hSyn-GRAB-DA2m (1e13 GC/mL, Addg140553, 100 nL in NAc), AAV9-hsyn-GRAB-DA-mut (1e13 GC/mL, Addg140555, 100 nL in NAc), AAV5-CAG-FLEX-rc[JAWS-KGC-GFP-ER2] (4e12 GC/mL, Addg84445, 300 nL in VTA) was a gift from Edward Boyden (Addgene viral prep # 84445-AAV5; http://n2t.net/addgene:84445; RRID: Addgene_84445).

For the experiments targeting periLC, the virus injection and optic fiber implantation were carried out as described previously (31). Briefly, the virus was injected at two depths in the periLC: A/P lambda −23% distance between bregma and lambda (−0.87 mm to −1 mm), M/L ± 0.8 mm, dorsal brain surface −2.7 mm/−2.8 mm. For axon projection photoinhibition, optic fibers were implanted bilaterally in the bed nucleus of the stria terminalis (BNST) (bregma 0.25 mm, M/L ±0.70 mm, D/V −3.35 mm), lateral hypothalamic area (LHA) (bregma −1.43 mm, M/L ±1.2 mm, D/V −4.5 mm), VTA (bregma −3.2 mm, M/L ±0.45 mm, D/V −4.3 mm), and Parvocellular Reticular Formation (PCRt) (bregma −6.35 mm, M/L ±1 mm, D/V −4.6 mm). For experiments targeting VTA, the virus was injected: bregma −3.2 mm, M/L ± 0.45 mm, dorsal surface −4.3 mm. In the same surgery, optic fibers were implanted unilaterally or bilaterally above the opsin/GCaMP-expressing neurons, targeting 0.2–0.5 mm above the injection sites. For experiments targeting NAc, the virus was injected: bregma +1.0 mm, M/L 1.20 mm, dorsal surface −4.5 mm. In the same surgery, optic fibers were implanted unilaterally above the GRAB-DA2m or GRAB-DA-mut expressing NAc neurons, targeted 200 μm above the injection sites. For axon projection photostimulation, a 200 μm optical fiber (Doric) was implanted unilaterally above the VTA. For VTA^DA^ neuron photostimulation and calcium activity recording, an optical fiber was implanted 200 μm dorsal to the virus injection sites unilaterally in the VTA. For VTA^DA^ neuron photoinhibition, an optical fiber was implanted 500 μm dorsal to the virus injection sites unilaterally in the VTA. The anatomical definition of the periLC is detailed in our previous work (31).

### Self-paced consumption

Unless noted, all mice in this study had access to mouse chow *ad libitum* in their home cage. They were given access to higher palatability foods in a separate apparatus in 1-h to 2-h sessions. A behavioral chamber (29 cm × 29 cm for Fig. 1, J to V, and 15 cm × 15 cm for all other figures), equipped with a slot for a lickometer spout was used for the food consumption experiments. A capacitive sensor was attached to the lickometer spout, where every 3 licks triggered a syringe pump to deliver 3 μL of food (Ensure). Lick events were continuously recorded by an Arduino board throughout the session. *Ad libitum* chow-fed or food-restricted mice underwent at least two habituation sessions in the chamber to acclimate to lickometer delivery of palatable liquid food (Ensure, Abbott). For behavioral sessions, freely moving mice had free access to Ensure *via* the lickometer spout for either 60 or 120 minutes in each self-paced consumption session. During each self-paced consumption session, mice typically performed multiple consumption bouts.

#### Constant palatability sessions

To measure the microstructure of feeding behaviors with different palatability in separate sessions, mice first performed a consumption session receiving higher palatability 100% Ensure. On a subsequent day, mice performed a session receiving lower palatability Ensure (20% Ensure/80% water).

#### Variable palatability sessions

To test hedonic contrast, we used double-barreled lick spouts with one spout connected to a syringe pump delivering 100% Ensure and the other spout delivering 20% Ensure or adulterating 100% Ensure with 3 mM quinine (Quinine hydrochloride dihydrate, Sigma-Aldrich, Q1125). The session was divided into 60 alternating 2-minute blocks where licking the spout would deliver either 100% Ensure or 20% Ensure or adulterating 100% Ensure with quinine (3μl/3 licks). An Arduino MEGA board was used to control the block structure that switched the pump triggered by lick signals from the lickometer.

#### Feeding microstructure bout analysis

We defined a feeding bout as an interlick interval (ILI) greater than the shortest 10% of times in which the mouse was in the food zone (FZ), which was based on the notion that a feeding bout ended when the mouse leaves the FZ (see fig. S2). We defined the FZ as one body length (mouth to tail-base) from the lick spout. To empirically determine FZ size and food zone time (FZT), mouse feeding behavior was first recorded using grayscale videos captured at 30 frames per second (fps), allowing for precise monitoring of the animal’s posture and position during self-paced consumption of palatable food (100% Ensure). To calculate the body length, we analyzed video tracking data by identifying key anatomical points, including the mouth and tail-base, within the cage using SLEAP (Social LEAP Estimates Animal Poses; www.sleap.ai). For each video frame, the positional coordinates of the tracked key points were mapped relative to the spout to determine whether the animal was inside or outside the FZ. FZT was defined as the interval between entering and exiting the FZ during a feeding episode. To define the radius of the FZ, the distance between the mouse’s mouth and the tail-base was measured across multiple feeding sessions. This distance, corresponding to the 95th percentile of the mouth-to-tail-base distribution, was determined to be 9.4 cm. For the FZ radius defined by body length (9.4 cm), the shortest 10% of the FZT value was 5-s, which was used as the ILI to define the bout threshold.

Licking events, number of bouts (bout initiation events), and bout duration were quantified for each food intake session using a custom MATLAB script. To determine the fundamental lick oscillator interval (56), we calculated the mode of the distribution of ILIs. Energy intake from Ensure was calculated by multiplying the volume consumed by its caloric content, based on the manufacturer’s information (220 kcal per 237 ml).

### Optogenetic conditions

For periLC^VGLUT2^ axon projection photoinhibition by ArchT in awake-behaving animals, 593 nm light output at the fiber tip was maintained between 6 and 10 mW. For periLC^VGLUT2^ axon projection photostimulation by ChR2 (87) in awake-behaving animals, 470 nm light output at the fiber tip was set between 6–10 mW, using a pulse protocol (25 ms pulse width, 20 pulses/s for 1 s). For periLC^VGLUT2^ axon projection photostimulation by Chrimson in awake-behaving animals, 635 nm light output at the fiber tip was set between 1–10 mW, using a pulse protocol (25 ms pulse width, 20 pulses/s for 1 s). For optogenetic activation of VTA^DA^ neurons by Chrimson-2A-GCaMP8s, we used 635 nm (power at fiber tip: 1–10 mW) with a pulse protocol (25 ms pulse width, 20 pulses/s for 1 second). For optogenetic activation of VTA^DA^ neurons by Chrimson we used 635 nm (power at fiber tip: 0.2–10 mW) with a pulse protocol (25 ms pulse width, 20 pulses/s for 1 second or 5 ms pulse width, 20 pulses/s for 150 ms). For optogenetic activation of periLC^VGLUT2^ neurons or periLC^VGLUT2^ axons in VTA by Chrimson we used 635 nm (power at fiber tip: 1–5 mW) with a pulse protocol (25 ms pulse width, 20 pulses/s for 1 second or 10 seconds).

For photoinhibition with 635 nm light (10 mW at tip) was triggered for 1 s following each lick and following cessation of licking for >1 s, this ramped off over 1 s, which is a procedure reported to reduce rebound firing (57). Control mice expressing only GFP/GCaMP underwent the same procedures and received identical laser stimulation intensities and procedures.

### Optogenetic modulation of food intake

For *in vivo* optogenetic activation of VTA^DA^ neurons or optogenetic inhibition of periLC^VGLUT2^ neuronal projections in awake-behaving animals, 20% Ensure was delivered through tubing connected to the lickometer spout for 60 or 120 minutes. Conversely, for *in vivo* optogenetic inhibition of VTA^DA^ neurons or optogenetic activation of periLC^VGLUT2^ neurons or periLC^VGLUT2^ neuronal projections in awake-behaving animals, 100% Ensure was delivered through the same setup for 60 or 120 minutes.

### Closed-loop optogenetic neuromodulation during food intake experiments

The closed-loop food intake experiment for *in vivo* photoactivation or photoinhibition of periLC^VGLUT2^ axonal projections was done similarly as described previously (31). Briefly, the behavior chamber and liquid-food delivery setup were as described above for the closed-loop food intake experiment (fig. S2A). 593 and 470 nm laser (SLOC) were aligned to a shutter (Uniblitz), which controlled laser light delivery to the mouse brain via a fiber launch (PAF-X-Z-A, Thorlabs). A relay box controlled the shutter opening, which had two dependencies: information about the experimental block (laser-ON or laser-OFF) from Arduino Board I and lick events (for contingent perturbation) or pseudo-events information (for noncontingent perturbation) from Arduino Board II. Arduino Board I sent out a signal representing the block identity to the relay box. Arduino Board II recorded mouse licks detected by the capacitive sensor. All lick, block, and optogenetic pulse events were recorded synchronously.

#### Lick-contingent optogenetics

In the lick-contingent sessions, licks recorded by Arduino Board II triggered an event signal delivered to the relay box that opened the shutter in laser-ON blocks, delivering light pulses to the brain. During the laser-OFF blocks, signals from the lick-sensor were recorded and led to food delivery but did not open the shutter, thus no light was delivered to the brain in OFF blocks.

#### Noncontingent optogenetics

In the noncontingent perturbation sessions, Arduino Board II generated pseudo-event signals to the relay box to open the shutter in the laser-ON blocks based on a pre-programmed sequence derived from the previous lick-contingent perturbation session for each mouse. During the laser-OFF blocks, signals from the lick-sensor were recorded and led to food delivery but did not open the shutter, thus no light was delivered to the brain in OFF blocks. In this way, mice experienced the same exposure to optogenetic perturbation as the lick-contingent sessions but independently of lick events. This is equivalent to open-loop perturbation with the same inhibition time as the lick-contingent closed-loop sessions.

#### periLC^VGLUT2^ axon photoinhibition and photostimulation

Circadian rhythm reversed (light 21:00 / dark 9:00) periLC^VGLUT2^ ArchT mice that had *ad libitum* food went through at least five days to acclimate to Ensure consumption before closed-loop perturbation. Over the first two days, mice had free access to 100% Ensure consumption in the same chamber used for closed-loop perturbation (1 h/day). For mice with 100% Ensure consumption less than that criterion, one more Ensure consumption session was given the next day. Mice whose Ensure consumption reached 900 licks/h were then acclimated to diluted Ensure (20%) consumption over the next three days (1 h/day). Mice with total licks above 600 licks/h in 20% Ensure consumption were selected for subsequent experiments. Sessions of lick-contingent and noncontingent optogenetic paradigms were performed on consecutive days. For each mouse, the noncontingent conditions delivered the same number of shutter openings as were determined in the contingent experiments by preprogramming these as pseudo-events with random inter-event intervals within the laser-ON blocks. Mice had free access to diluted Ensure (20%) in the chamber during the consumption sessions. The photoinhibition sessions were divided into alternating 2-min laser-OFF and laser-ON blocks. The sessions for photostimulation of periLC^VGLUT2^ neuronal projections in the VTA used 10-minute blocks.

#### VTA^DA^ neuron optogenetic perturbations

The procedure for closed-loop perturbation of VTA^DA^ neuron activity during food intake was similar to the previously described perturbations, including the use of a liquid-food delivery setup, and 2-minute ON/OFF blockwise contingent and noncontingent photostimulation settings. However, for this experiment, a smaller behavior chamber (the size was changed to 15 cm x15 cm) and a customized Bonsai-assisted Neurophotometrics fiber photometry system were utilized for closed-loop perturbation of VTA^DA^ neuron activity during food intake. In these experiments, optogenetic neuromodulation of consummatory behavior was performed in *Slc6a3-IRES-Cre* mice. These mice were injected with Cre-dependent AAVs expressing red-shifted opsins (Chrimson or JAWS) and/or AAVs expressing fluorogenic transgenes such as GCaMP8 or GFP into the VTA^DA^ neurons, followed by optical fiber implantation over the VTA. Lick and food delivery events were recorded using an Arduino Mega Board, and this data was synchronized with the Bonsai-assisted Neurophotometrics system.

The Bonsai-assisted Neurophotometrics system simultaneously measured calcium dynamics triggered 635 nm laser signals and controlled the experimental block information (laser-ON or laser-OFF) for the 2-minute intervals. Optogenetic stimulation or inhibition was carried out using the FP3002 system (Neurophotometrics). A fiber optic cable connected to a 635 nm laser was attached to the fiber optic implants via a ceramic sleeve. 635 nm excitation (1 s, 20 Hz, 25 ms pulse width or 150 ms, 20 Hz, 5 ms pulse width for dopamine release-calibrated photostimulation experiment) or 635 nm inhibition (1 s, 20 Hz, 50 ms pulse width and ramped down over another second) was triggered by TTL pulses sent to the rig upon onset of each lick. The microstructure of feeding behavior was then compared between laser-ON and laser-OFF blocks during contingent and noncontingent photostimulation or photoinhibition of VTA^DA^ neurons.

### Analysis of closed-loop perturbation on food consumption

All behavioral analysis was performed using custom MATLAB scripts. Lick events from the two different experimental blocks were separately grouped for further analysis. Only blocks in which mice initiated licking were included in the calculation of average licks per block. To compute the licking rate across bouts (e.g., laser ON vs. OFF or high vs. low palatability, as in Figures 2K, 4S, 4Y, 5F, 5J, and figs. S2H and S2K), we first identified each feeding bout and divided it into 1-second bins. These bouts were aligned by the bout start time and the mean of lick rates in each bin across each bouts yielded the mean bout licking rate profile. We focus on the comparison of the first 20 seconds, where divergence typically appears (before 10 seconds). Rates were compared between laser-ON and laser-OFF periods for each second. Bout durations and bout numbers from all laser-ON and laser-OFF blocks within the same session were collected and analyzed.

### Conditioned place preference

A two-chambered apparatus was used as described previously (31). ***Ad libitum*-fed** mice were acclimatized to the apparatus on the first day. On the second day, the **pre-test preference score** was determined after the mice were allowed to freely explore the apparatus. Over the next two days, a **593 nm laser was activated** when periLC^VGLUT2^ ArchT→VTA mice entered the initially less preferred chamber. Each session lasted 15 minutes. **Mice were tethered to optical fibers** even on test days without optical perturbation. A camera installed above the apparatus (30 Hz, Basler) tracked the mouse position throughout the session.

### Fiber photometry recordings

The commercial fiber photometry FP3002 system (Neurophotometrics, CA) was used to measure fluorescent signals using 470 nm (active green signal for GCaMP or GRAB-DA2m) and 415 nm (isosbestic reference) excitation light, delivered through a patch cord in interleaved LED pulses. Both the fluorescence excitation and emission light were delivered and collected through a 200 μm diameter optical fiber (0.39 NA, Doric Lenses, Canada). The light was reflected through a dichroic mirror and onto a 20× Olympus objective, and the emitted fluorescent light was captured by a high quantum efficiency sCMOS camera integrated into the Neurophotometrics system. Signals were acquired and synchronized with behavioral events using Bonsai.

Before fiber photometry recording, a fluorogenic transgene, GCaMP8s or GCaMP8m or GFP, was expressed in the VTA^DA^ neurons of male and female *Slc6a3-IRES-Cre* mice, or GRAB-DA2m_,_ or GRAB-rDA-mut in the NAc of male and female *Slc6a3-IRES-Cre, Vglut2-IRES-Cre* or C57BL6/J mice, and a short optical fiber (200 μm diameter, 0.39 NA, Doric Lenses, Canada) was placed in a ceramic ferrule and inserted toward the target region. To enable AAV expression and recovery, mice were housed individually for at least 2 weeks following virus injection. During behavioral experiments, mice were connected to a fiber optic patch cable linked to the photometry system, which delivered excitation light and collected fluorescence emission during behaviors. Two LEDs emitting light at 470 nm (for Ca^2+^-dependent GCaMP fluorescence) and the isosbestic wavelength (415 nm) (for bleaching and minor motion artifact correction) were combined via dichroic mirrors and transmitted through a patch cord connected to the implanted optical fiber. To minimize GCaMP or GRAB-DA2m bleaching, the laser power for 470 nm and 415 nm at the fiber tip was low (0.05–0.07 mW).

Using this setup, fluorescent signals (20 Hz for each channel) and behavioral events were collected using a custom Bonsai workflow. Mice were allowed to explore the apparatus and underwent repeated photometry sessions in conjunction with the behavioral sessions associated with palatable food intake.

To test whether palatability and consumption bout duration were associated with different levels of VTA^DA^ neuron activity during constant palatability sessions, *Slc6a3-IRES-Cre* mice were transduced with the genetically encoded calcium indicator GCaMP8s and the red-shifted opsin, Chrimson (AAV-hSyn-DIO-jGCaMP8s-P2A-Chrimson). Fiber photometry was then used to measure VTA^DA^ neuron calcium dynamics during separate experimental sessions where mice were given either higher palatability (100% Ensure on day 1) or lower palatability Ensure (20% Ensure on day 2). VTA^DA^ neuron activities and feeding behaviors during these sessions were compared. 10 of the total 23 *Slc6a3-IRES-Cre* mice with incorrectly targeted injections or fiber implantations were excluded. In another set of experiments, *Slc6a3-IRES-Cre* mice were transduced with only GCaMP8m (AAV9-hSyn-FLEX-GCaMP8m). Using fiber photometry, VTA^DA^ neuron calcium dynamics were measured during sessions delivering higher palatability (100% Ensure on day 1) and lower palatability Ensure (20% Ensure on day 2). VTA^DA^ neuron activities for these different palatability conditions were compared.

To measure VTA^DA^ neuron activity with different hunger states during eating, *Slc6a3-IRES-Cre* mice were transduced with GCaMP8m (AAV9-hSyn-FLEX-GCaMP8m). Mice were food-restricted to 85% of baseline body weight. Fiber photometry was used to measure VTA^DA^ neuron calcium dynamics in mice receiving 20% Ensure during this food-restricted hunger state. Mice were allowed to regain normal body weight with *ad libitum* access to chow food in the home cage. In a separate session, these mice were tested receiving 20% Ensure.

To measure VTA^DA^ neuron activity with LiCl injection during Ensure consumption, *Slc6a3-IRES-Cre* mice were transduced with GCaMP8m (AAV9-hSyn-FLEX-GCaMP8m). Fiber photometry was used to measure VTA^DA^ neuron calcium dynamics in mice consumed 100% Ensure 1-h before and 2-h after PBS injection (to include enough lick events). On a separate day, mice first consumed 100% Ensure 1-h before and 2-h after Lithium Chloride (LiCl, 50 mg/kg) injection.

A separate group of *Slc6a3-IRES-Cre* mice was transduced with only GFP. Fiber photometry was used to measure VTA^DA^ neuron calcium dynamics during a session delivering 20% Ensure. The photometry dynamics during food intake were quantified and compared with the bout durations.

To test whether palatability and consumption bout duration were associated with different levels of VTA^DA^ neuron activity during variable palatability sessions, *Slc6a3-IRES-Cre* mice were transduced with GCaMP8s and Chrimson (AAV-hSyn-DIO-jGCaMP8s-P2A-Chrimson). Fiber photometry was used to measure VTA^DA^ neuron calcium dynamics in mice that have access to both a palatable liquid food source and the same food diluted with water to reduce palatability (100% vs 20% Ensure). VTA^DA^ neuron activities and feeding behaviors were compared between the higher and lower palatability conditions. To test whether palatability and consumption bout duration were associated with different levels of VTA^DA^ neuron activity during additional variable palatability sessions, *Slc6a3-IRES-Cre* mice were transduced with GCaMP8m (AAV9-hSyn-FLEX-GCaMP8m). Fiber photometry was used to measure VTA^DA^ neuron calcium dynamics in which mice have access to 100% Ensure or 100% Ensure adulterated with 3 mM quinine. VTA^DA^ neuron activities and feeding behaviors were compared between the higher and lower palatability conditions.

To test whether palatability and consumption bout duration were associated with different levels of dopamine in NAc during constant palatability sessions, mice were transduced with the genetically encoded dopamine indicator GRAB-DA2m. Fiber photometry was then used to measure dopamine in NAc neurons during separate experimental sessions where mice were given either higher palatability (100% Ensure on day 1) or lower palatability Ensure (20% Ensure on day 2). NAc GRAB-DA2m response and feeding behaviors during these sessions were compared. To test whether palatability and consumption bout duration were associated with different levels of dopamine in the NAc during variable palatability sessions, the mice form constant palatability sessions were used. Mice had access to both a palatable liquid food source and the same food diluted with water to reduce palatability (100% vs 20% Ensure). NAc GRAB-DA2m response and feeding behaviors were compared between the higher and lower palatability conditions.

A separate group of mice was transduced with the genetically encoded dopamine indicator mutant GRAB-rDA-mut in NAc. Fiber photometry was used to measure the response during a session delivering higher palatability Ensure (100% Ensure).

### Analysis of fiber photometry data

Fiber-photometry recordings and associated behavioral data were analyzed using MATLAB. To minimize the influence of motion and artifacts, the calcium-dependent fluorescence signal (470 nm) from GCaMP or the dopamine-dependent fluorescence signal (470 nm) from GRAB-DA2m was normalized to the calcium-independent isosbestic signal (415 nm) for analysis (for GRAB-DA2m, the isosbestic signal is not exactly 415 nm, but this approach is still valid for removing motion artifacts). To do this, the 415 nm signal was linearly scaled to fit the 470 nm GCaMP signal (using the MATLAB function: robustfit). For GRAB-DA2m analysis, we observed slow bleaching rates (minutes) that were different for the 415 nm and 470 nm excitation channels. To address this, the 415 nm excitation fluorescence signal was split into 5 min chunks that were linearly scaled to fit the 470 nm GRAB-DA signal (using the MATLAB function: robustfit) and concatenated. The change in fluorescence (ΔF) at each time point was then calculated by dividing the fitted 415 nm signal from the 470 nm signal (470 nm/fitted 415 nm). This approach minimized differential photobleaching and motion artifacts.

The resulting (ΔF) signal was z-scored by subtracting the baseline fluorescence (mean signal during non-consummatory periods) and dividing by the standard deviation of the baseline fluorescence. The z-scored photometry signal, reflecting VTA^DA^ neuron activity, was aligned to the onset of licking during individual food consumption bouts. For comparisons in sessions divided into alternating 2-minute blocks, we included bouts that did not span across two separate blocks. Data were visualized as heatmaps representing the z-scored signals across all feeding bouts for each animal.

#### Mean GCaMP or GRAB-DA response plots for all bouts

We generated two types of GCaMP or GRAB-DA response plots as peri-stimulus time histograms (PSTHs). The first (blue) shows mean PSTH responses across all bouts from −5 to 20 seconds, without accounting for variable bout durations. Since bouts end at different times, this can lower mean responses for the later consumption phase because the responses during longer bouts are penalized by low responses in bouts that have ended. The second plot (magenta) is the variable-length time mean response across all bouts. This accounts for variable bout duration by padding ended bouts with NaNs and calculating the ‘nanmean’ response within each bout and thus avoids penalizing the mean GCaMP8s of GRAB-DA responses for longer bouts, more accurately reflecting neural activity throughout consumption.

#### Photometry AUC

For each individual bout, the area under the curve (AUC) of the aligned z-scored photometry signal was computed using a trapezoidal integration function in MATLAB. The AUC values were then analyzed against bout duration with linear regression to assess the relationship between neuronal activity and consumption duration. This was performed by using all the bouts from each subject. These metrics were also reported as the mean across bouts for each animal and compared between animals for different perturbation or palatability conditions.

#### Regression of GCaMP or GRAB-DA response across a session

To assess the relationship between neuronal activity and consumption timing across all animals, bout duration and AUC of VTA^DA^ neuron GCaMP responses or GRAB-DA responses in NAc were examined for all bouts in a session and then analyzed against bout indices with linear regression.

In the photoactivation experiments, bout duration and AUC values of VTA^DA^ neuron GCaMP or GRAB-DA responses in NAc responses were compared for ON or OFF blocks of a 120-minute session within each animal.

### VTA^DA^ neuron transduction-normalized calibrated photostimulation and supraphysiological photostimulation

To investigate a causal relationship between VTA^DA^ neuron activity and consumption bout duration, we tested whether boosting VTA^DA^ neuron activity with an approach for photometry-calibrated photostimulation in response to consuming lower palatability food (20% Ensure) would extend bout duration. *Slc6a3-IRES-Cre* mice were transduced with the genetically encoded calcium indicator GCaMP8s and the red-shifted opsin Chrimson (AAV-hSyn-DIO-jGCaMP8s-P2A-Chrimson), and fiber cannulas (0.39 NA, 200 μm) were implanted unilaterally above the VTA. Co-expression of GCaMP8s and Chrimson in VTA^DA^ neurons allowed simultaneous red light (635 nm) activation and monitoring of calcium dynamics.

After acclimating mice to palatable food consumption using our lickometer-driven liquid food delivery system, we used fiber photometry to measure VTA^DA^ neuron activity in response to higher palatability (100% Ensure) and lower palatability (20% Ensure) food. We then determined the level of optogenetic activation needed to increase VTA^DA^ neuron activity during 20% Ensure consumption to levels observed during 100% Ensure consumption, using a customized Neurophotometrics system. Because our optogenetic perturbation would only affect the fraction of VTA^DA^ neurons transduced with Chrimson, we pursued a strategy to scale the activation of this subset to approximate the level of activity that would be attained if 100% of VTA^DA^ neurons were transduced. Taking into account a partial transduction efficiency of Chrimson (30–50% from pilot studies), we calibrated the photostimulation of the VTA^DA^ neuron response during 20% Ensure consumption to approximately double the response level of 100% Ensure consumption in the absence of photostimulation. For each lick, photostimulation was delivered with a pulse frequency of 20 Hz, a pulse width of 25 ms, and a stimulation time of 1 second at a power of 1–2 mW at the fiber tip. For bouts greater than 1-s bout duration, the 1-s laser stimulation was reinitiated with each successive lick until an ILI > 1 s.

Mice then underwent a 120-minute session of self-initiated 20% Ensure consumption with calibrated photostimulation. The session was divided into alternating 2-minute blocks: in one condition, the laser was triggered contingent on food consumption, and in another, food was delivered without laser activation. In the lick-triggered noncontingent photostimulation condition, laser activation was randomized to match the lick statistics of the contingent session. Fiber photometry measured neural dynamics during 20% Ensure consumption and laser stimulation in both conditions. *Post hoc* immunohistochemistry analysis of the mouse brains measured tyrosine hydroxylase (TH)-positive neurons co-expressing GCaMP8s.

To evaluate the behavioral effects of laser stimulation in control groups, *Slc6a3-IRES-Cre* mice were transduced with GCaMP8m or GFP (without opsin) and implanted with fiber cannulas unilaterally above the VTA. These mice received the same intensity and pattern of laser light pulses as the photometry-calibrated group during food consumption. Consumption duration and bout number were compared between laser-ON and laser-OFF blocks.

We also assessed the effects of supraphysiological photostimulation of VTA^DA^ neurons on food consumption, while simultaneously measuring calcium dynamics. High-intensity optogenetic stimulation (10 mW, 20 Hz, 25 ms pulse width for 1 second) was applied using an alternating block-wise (laser-ON/laser-OFF) contingent photostimulation protocol during 20% Ensure consumption. Consumption duration, bout number, and the mean rate of food intake during laser-ON and laser-OFF blocks were compared across sessions for both photometry-calibrated and supraphysiological photostimulation.

### NAc dopamine-calibrated photostimulation and supraphysiological photostimulation

To investigate a causal relationship between VTA^DA^ neuron activity and consumption bout duration, we also tested whether boosting VTA^DA^ neuron activity using dopamine in NAc to calibrate photostimulation. *Slc6a3-IRES-Cre* mice were transduced with Chrimson (AAV5-hSyn-FLEX-rc-ChrimsonR-tdTomato unilaterally into VTA, and the genetically encoded dopamine indicator GRAB-DA2m unilaterally in the NAc. Optical fibers (0.39 NA, 200 μm) were implanted unilaterally above the VTA and NAc viral injection sites.

After acclimating mice to palatable food consumption using our lickometer-driven liquid food delivery system, we used fiber photometry to measure GRAB-DA2m in NAc in response to higher palatability (100% Ensure) and lower palatability (20% Ensure) food. We then determined the level of optogenetic activation needed to increase VTA^DA^ neuron activity during 20% Ensure consumption to levels observed during 100% Ensure consumption. For each lick, photostimulation was delivered with a pulse frequency of 20 Hz, a pulse width of 5 ms, and a stimulation time of 150 ms at a power of 0.2–0.4 mW at the fiber tip.

Mice then underwent a 120-minute session of self-initiated 20% Ensure consumption with NAc GRAB-DA2m calibrated photostimulation. The session was divided into alternating 2-minute blocks: in one condition, the laser was triggered contingent on food consumption, and in another, food was delivered without laser activation. Fiber photometry measured dopamine dynamics in NAc during 20% Ensure consumption and laser stimulation in both conditions.

We also assessed the effects of supraphysiological photostimulation of VTA^DA^ neurons on food consumption, while simultaneously measuring dopamine dynamics in NAc. High-intensity optogenetic stimulation (10 mW, 20 Hz, 25 ms pulse width for 1 second) was applied using an alternating block-wise (laser-ON/laser-OFF) contingent photostimulation protocol during 20% Ensure consumption. Consumption duration, bout number, and the mean rate of food intake during laser-ON and laser-OFF blocks were compared across sessions for both dopamine**-**calibrated and supraphysiological photostimulation.

### periLC^VGLUT2^ axon photoinhibition and photostimulation

Circadian rhythm reversed (light 21:00 / dark 9:00) periLC^VGLUT2^ ArchT mice that had *ad libitum* food went through at least five days to acclimate to Ensure consumption before closed-loop perturbation. Over the first two days, mice had free access to 100% Ensure consumption in the same chamber used for closed-loop perturbation (1 h/day). For mice with 100% Ensure consumption less than that criterion, one more Ensure consumption session was given the next day. Mice whose Ensure consumption reached 900 licks/h were then acclimated to diluted Ensure (20%) consumption over the next three days (1 h/day). Mice with total licks above 600 licks/h in 20% Ensure consumption were selected for subsequent experiments. Sessions of lick-contingent and noncontingent optogenetic paradigms were performed on consecutive days. For each mouse, the noncontingent conditions delivered the same number of shutter openings as were determined in the contingent experiments by preprogramming these as pseudo-events with random inter-event intervals within the laser-ON blocks. Mice had free access to diluted Ensure (20%) in the chamber during the consumption sessions. The photoinhibition sessions were divided into alternating 2-min laser-OFF and laser-ON blocks. The sessions for photostimulation of periLC^VGLUT2^ neuronal projections in the VTA used 10-minute blocks.

### VTA^DA^ neuron dynamics and feeding behaviors during periLC^VGLUT2^ axon photostimulation

To investigate a causal relationship between VTA^DA^ neuron activity and consumption bout duration with periLC^VGLUT2^ axon photostimulation, we tested whether periLC^VGLUT2^ axon photostimulation in VTA affects the feeding behaviors and VTA^DA^ neuron activity. *Vglut2-(Slc17a6)-IRES-Cre* mice were transduced with the red-shifted opsin Chrimson (AAV5-hSyn-FLEX-rc(ChrimsonR-tdTomato) unilaterally into periLC, VTA were transduced with the genetically encoded GCaMP8m (AAV9-hSyn-FLEX-GCaMP8m), fiber cannulas (0.39 NA, 200 μm) were implanted unilaterally above the viral injection site of VTA.

After acclimating mice to palatable food consumption using our lickometer-driven liquid food delivery system, mice then underwent a 120-minute session of self-initiated 100% Ensure consumption with lick-contingent photostimulation of the periLC^VGLUT2^ axon in VTA during 100% Ensure consumption. For each lick, photostimulation was delivered with a pulse frequency of 20 Hz, a pulse width of 25 ms, and a stimulation time of 1 second at a power of 2–5 mW at the fiber tip. We assessed the effects of photostimulation of periLC^VGLUT2^ neurons on VTA^DA^ neuron activity when there is no food provided in the chamber. For each stimulation, photostimulation was delivered with a pulse frequency of 20 Hz, a pulse width of 25 ms, and a stimulation time of 10 s at a power of 2–5 mW at the fiber tip. VTA^DA^ neuron activity was compared before and during photostimulation. The session was also divided into alternating 2-minute blocks: in one condition, the laser was triggered contingent on food consumption, and in another, food was delivered without laser activation. Fiber photometry measured neural dynamics during 100% Ensure consumption and laser stimulation in both conditions were compared.

### NAc dopamine during periLC^VGLUT2^ neuron photostimulation

To investigate a causal relationship between dopamine in NAc and consumption bout duration with periLC^VGLUT2^ neuron photostimulation, we tested whether boosting periLC^VGLUT2^ neuron activity affects dopamine in NAc and feeding behaviors. *Vglut2-(Slc17a6)-IRES-Cre* mice were transduced with the red-shifted opsin Chrimson (AAV5-hSyn-FLEX-rc(ChrimsonR-tdTomato) unilaterally into periLC, NAc were transduced with the genetically encoded dopamine indicator GRAB-DA2m, fiber cannulas (0.39 NA, 200 μm) were implanted unilaterally above the periLC and NAc viral injection site.

After acclimating mice to palatable food consumption using our lickometer-driven liquid food delivery system, we used fiber photometry to measure dopamine in NAc in response to higher palatability (100% Ensure) and lower palatability (20% Ensure) food. We assessed the effects of photostimulation of periLC^VGLUT2^ neurons on dopamine in NAc when there is no food provided in the chamber. For each stimulation, photostimulation was delivered with a pulse frequency of 20 Hz, a pulse width of 25 ms, and a stimulation time of 10s at a power of 1–2 mW at the fiber tip. GRAB-DA2m response in NAc was compared before and during photostimulation.

Mice then underwent a 120-minute session of self-initiated 100% Ensure consumption with lick-contingent photostimulation of the periLC^VGLUT2^ neuron during 100% Ensure consumption. For each lick, photostimulation was delivered with a pulse frequency of 20 Hz, a pulse width of 25 ms, and a stimulation time of 1s at a power of 1–2 mW at the fiber tip. The session was divided into alternating 2-minute blocks: in one condition, the laser was triggered contingent on food consumption, and in another, food was delivered without laser activation. Fiber photometry measured neural dynamics during 100% Ensure consumption and laser stimulation in both conditions.

### Weight loss drug treatment on appetite and VTA^DA^ activity.

To investigate the effect of weight loss drug treatment on palatable food consumption, a crossover design was used to compare vehicle (PBS) and semaglutide (Cayman Chemical, No. 29969) treatments. Mice with ad libitum food access received either PBS or semaglutide injections. In humans, semaglutide therapy typically includes a lead-in period to minimize nausea, and a similar approach was applied to the mice. *Ad libitum* chow-fed C57BL/6J mice received a PBS injection on day 0. Subsequently, mice were dosed with semaglutide at 0.05 mg/kg on day 1, 0.1 mg/kg on day 2, and 0.15 mg/kg daily on days 3–5. Body weight and homecage chow intake were monitored daily. To minimize the immediate aversive effects of semaglutide on food intake, the mice were given 2-hour access to palatable liquid food (100% undiluted Ensure) daily, beginning 120 minutes after vehicle or semaglutide treatment. Mice were allowed to regain weight with PBS injections on days 6–8. From days 9–16, the mice that received semaglutide were retested using the same procedures but with daily PBS injections. Mice previously receiving PBS were retested with semaglutide injections over five days, followed by three additional recovery days.

To investigate the effect of weight loss drug treatment on VTA^DA^ neuron activity and its influence on appetite, we followed a similar procedure using PBS or semaglutide treatment while monitoring VTA^DA^ neuron activity in *Slc6a3-IRES-Cre* mice. Specifically, these mice were transduced with the genetically encoded calcium indicator GCaMP8s and the red-shifted opsin Chrimson (AAV-hSyn-DIO-jGCaMP8s-P2A-Chrimson) and implanted with optical fibers (0.39 NA, 200 μm) unilaterally above the VTA. After recovery from virus injection surgery and habituation to Ensure intake, mice were dosed with semaglutide following the above procedure for days 0–16. During the treatment phases, mice were given 1-hour access to palatable liquid food (undiluted Ensure) after 2 hours of vehicle or semaglutide treatment each day. Calcium signals from VTA^DA^ neurons were recorded during Ensure consumption, and the AUC of GCaMP activity were compared across treatment days for both vehicle and semaglutide groups.

Since semaglutide initially reduced bout duration and VTA^DA^ neuron responses to palatable food, we then tested whether lick-triggered photostimulation of VTA^DA^ neurons could extend bout duration during semaglutide treatment. After a 3-day recovery following the initial semaglutide treatment, mice received a second round of semaglutide using the same procedure, including lick-contingent optogenetic neuromodulation on semaglutide day 1 (0.05 mg/kg). Photostimulation on day 1 was calibrated to restore VTA^DA^ neuron activity during 100% Ensure consumption to levels observed before semaglutide treatment. Stimulation was delivered at 20 Hz, with 25 ms pulses for 1 second, at 1–2 mW power at the fiber tip, with the laser in continuous mode. Ensure consumption was assessed over 1 hour, comparing Laser ON and Laser OFF blocks in semaglutide-treated mice.

### Photoinhibition of VTA^DA^ activity during appetite recovery with weight loss drug treatment

To examine the role of VTA^DA^ neurons in the recovery of palatable food intake during semaglutide dose escalation, the red-shifted opsin JAWS was expressed in VTA^DA^ neurons of male and female *Slc6a3-IRES-Cre* mice. Mice were dosed with semaglutide at 0.05 mg/kg on day 1, 0.1 mg/kg on day 2, and 0.15 mg/kg on days 3–6, with one dose administered daily. From day 4 to day 6, mice receiving semaglutide treatment underwent lick-contingent photoinhibition of VTA^DA^ neurons. Body weight, homecage chow intake, and 1-hour food intake of 100% Ensure were monitored. Daily 1-hour Ensure consumption during lick-contingent optogenetic inhibition was compared between Laser ON and Laser OFF blocks in the semaglutide-treated mice. Mean Ensure intake during Laser ON and Laser OFF blocks was analyzed for each animal, comparing the first 3 days to the last 3 days of the treatment.

For comparison, GFP or GCaMP was expressed in VTA^DA^ neurons of male and female *Slc6a3-IRES-Cre* mice. Similar experiments were conducted to assess the effect of optogenetic manipulation of VTA^DA^ neurons on Ensure food intake from day 4 to day 6 during high-dose semaglutide treatment in these control GFP or GCaMP mice.

### Histology and imaging

For histology, mice were transcardially perfused with 4% paraformaldehyde (PFA) in 0.1M phosphate buffer (pH 7.4). Following perfusion, tissues were post-fixed for 3–4 hours and then washed overnight in phosphate-buffered saline (PBS, pH 7.4). Brain slices were sectioned using a vibratome (50 μm, Leica VT1200S). Selected sections were mounted onto microscope slides using a mounting medium containing DAPI or NeuroTrace (1:500, Invitrogen, N21479). Brain sections were automatically imaged using slide scanner microscopes (Panoramic 250 or TissueFaxs 200).

For FISH, brain sections were incubated overnight at 4℃ with VGAT Probe (HCR v3.0, B5, from molecular instruments, Gene accession: NM_009508.2). The next day, sections were incubated at room temperature for 3 hours with a probe amplifier (HCR v3.0 Amplifier B5 from molecular instruments). Selected sections were mounted onto microscope slides using a mounting medium containing NeuroTrace. Brain sections were automatically imaged using a slide scanner microscope (TissueFaxs 200). For immunohistochemistry, brain sections were incubated overnight at 4℃ with a primary antibody against tyrosine hydroxylase (anti-TH, 1:1000, (Millipore Cat# AB152, RRID:AB_390204). The next day, sections were incubated at room temperature for 2 hours with minimally cross-reactive fluorophore-conjugated secondary antibodies (1:500, Jackson ImmunoResearch).

### QUANTIFICATION AND STATISTICAL ANALYSIS

Statistical values were represented as mean ± SEM unless otherwise stated. All the statistics were performed using GraphPad Prism, R, Python, or MATLAB. Pairwise comparisons were calculated by unpaired or paired t-tests. A repeated measures ANOVA with the Geisser-Greenhouse correction was used to account for violations of sphericity in the data. The main effects of treatment, time, and their interaction were examined. Post hoc comparisons were conducted using the Holm-Šídák’s multiple comparisons test. Comparison of distributions from feeding microstructure analysis were analyzed in the nonparametric two-sample Kolmogorov-Smirnov test. In the comparison of distributions of lick rate during variable palatability session, photostimulation, and photoinhibition, we used the glmmTMB package in R (https://cran.r-project.org/web/packages/glmmTMB/index.html) to fit negative binomial generalized linear mixed models (NB GLMM) to evaluate the effect of treatment condition (e.g., laser on vs. off) on lick number across time bins. The model included treatment condition, time bin, and their interaction (treatment condition × time bin) as fixed effects, with random intercepts for animals and bouts nested within animals to account for repeated measures within animals and bouts. Zero-inflated NB GLMM were used if they provided better goodness of fit than standard NB GLMM, as assessed using the likelihood ratio test (LRT), and if they had an overdispersion ratio of ~1. The zero-inflation formula used a time bin as a predictor since the observed proportion of zeros generally increased over time. Post-hoc pairwise comparisons between treatment conditions were conducted within each time bin using estimated marginal means (EMMs) derived from the full model via the emmeans package in R (https://cran.r-project.org/web/packages/emmeans/index.html). Differences between treatment conditions were assessed using Wald tests, and p-values were adjusted for multiple comparisons using the Benjamini-Hochberg method. The significance of the main effects of and interaction between the treatment condition and time bin was assessed using the LRT to compare models with and without a treatment condition term, time bin term, or interaction term. All analyses were performed in R (version 4.4.2). The results of statistical tests are summarized in Table S1. ns p > 0.05, *p < 0.05, **p < 0.01, ***p < 0.001.

## Supplementary Material

Supplementary Materials and Table S1

MDAR

## Data Availability

All data are available in the main text or the supplementary materials. The code is available at Github (https://github.com/neugun/periLC-VTA) and archived at Zenodo (https://doi.org/10.5281/zenodo.14680708). AAV-Ef1a-FLEX-rev-ChR2-EYFP and AAV-Syn-fDIO-mCherry-IRES-WGA-Cre are available from Karl Deisseroth under a material agreement with Stanford, AAV-hSyn-DIO-eGFP is available from Ian Wickersham under a material agreement with MIT, AAV9-hSyn-FLEX-GCaMP8m is available from The GENIE Project under a material agreement with Janelia Research Campus, AAV-hSyn-FLEX-rc-[ChrimsonR-tdTomato], AAV-Syn-FLPx-rc-[ChrimsonR-tdTomato], AAV-CAG-FLEX-ArchT-GFP, and AAV5-CAG-FLEX-rc[JAWS-KGC-GFP-ER2] are available from Edward Boyden under a material agreement with MIT, AAV-hSyn-GRAB-DA2m and AAV-hSyn-GRAB-DA-mut are available from Yulong Li under a material agreement with Peking University, AAV-hSyn-DIO-jGCaMP8s-P2A-ChrimsonR-ST is available from Mark Histed under a material agreement with NIMH.

## References

[1] Yeomans MR, Taste, palatability and the control of appetite. Proc. Nutr. Soc. 57, 609–615 (1998). 10096124 10.1079/pns19980089

[2] Johnson F, Wardle J, Variety, palatability, and obesity. Advances in nutrition (Bethesda, Md.) 5, 851–859 (2014). 25398751 10.3945/an.114.007120 PMC4224225

[3] Hebb DO, The Organization of Behavior. (Lawrence Erlbaum Associates, Mahwah, NJ, 1949/2002), pp. 335.

[4] Craig W, Appetites and aversions as constituents of instincts. Biol. Bull. 34, 91–107 (1918). 10.1073/pnas.3.12.685 PMC1091358 16586767

[5] Sternson SM, Eiselt AK, Three Pillars for the Neural Control of Appetite. Annu. Rev. Physiol, (2016). 10.1146/annurev-physiol-021115-104948 27912679

[6] Cowley MA , The distribution and mechanism of action of ghrelin in the CNS demonstrates a novel hypothalamic circuit regulating energy homeostasis. Neuron 37, 649–661 (2003). 12597862 10.1016/s0896-6273(03)00063-1

[7] Aponte Y, Atasoy D, Sternson SM, AGRP neurons are sufficient to orchestrate feeding behavior rapidly and without training. Nat. Neurosci. 14, 351–355 (2011). 21209617 10.1038/nn.2739 PMC3049940

[8] Krashes MJ , Rapid, reversible activation of AgRP neurons drives feeding behavior in mice. J Clin Invest 121, 1424–1428 (2011). 21364278 10.1172/JCI46229 PMC3069789

[9] Betley JN , Neurons for hunger and thirst transmit a negative-valence teaching signal. Nature 521, 180–185 (2015). 25915020 10.1038/nature14416 PMC4567040

[10] Han W. , A Neural Circuit for Gut-Induced Reward. Cell 175, 665–678.e623 (2018). 30245012 10.1016/j.cell.2018.08.049 PMC6195474

[11] Ly T. , Sequential appetite suppression by oral and visceral feedback to the brainstem. Nature 624, 130–137 (2023). 37993711 10.1038/s41586-023-06758-2 PMC10700140

[12] Campos CA, Bowen AJ, Schwartz MW, Palmiter RD, Parabrachial CGRP Neurons Control Meal Termination. Cell Metab. 23, 811–820 (2016). 27166945 10.1016/j.cmet.2016.04.006 PMC4867080

[13] Campos CA, Bowen AJ, Roman CW, Palmiter RD, Encoding of danger by parabrachial CGRP neurons. Nature 555, 617–622 (2018). 29562230 10.1038/nature25511 PMC6129987

[14] Small DM, Jones-Gotman M, Dagher A, Feeding-induced dopamine release in dorsal striatum correlates with meal pleasantness ratings in healthy human volunteers. Neuroimage 19, 1709–1715 (2003). 12948725 10.1016/s1053-8119(03)00253-2

[15] Cohen JY, Haesler S, Vong L, Lowell BB, Uchida N, Neuron-type-specific signals for reward and punishment in the ventral tegmental area. Nature 482, 85–88 (2012). 22258508 10.1038/nature10754 PMC3271183

[16] Engelhard B. , Specialized coding of sensory, motor and cognitive variables in VTA dopamine neurons. Nature 570, 509–513 (2019). 31142844 10.1038/s41586-019-1261-9 PMC7147811

[17] Tsai HC , Phasic firing in dopaminergic neurons is sufficient for behavioral conditioning. Science 324, 1080–1084 (2009). 19389999 10.1126/science.1168878 PMC5262197

[18] Domingos AI , Leptin regulates the reward value of nutrient. Nat. Neurosci. 14, 1562–1568 (2011). 22081158 10.1038/nn.2977 PMC4238286

[19] Mikhailova MA , Optogenetically-induced tonic dopamine release from VTA-nucleus accumbens projections inhibits reward consummatory behaviors. Neuroscience 333, 54–64 (2016). 27421228 10.1016/j.neuroscience.2016.07.006 PMC4992643

[20] Eleanor CS , Phasic Stimulation of Midbrain Dopamine Neuron Activity Reduces Salt Consumption. eneuro 5, ENEURO.0064–0018.2018 (2018). 10.1523/ENEURO.0064-18.2018 PMC5952649 29766048

[21] Boekhoudt L. , Does activation of midbrain dopamine neurons promote or reduce feeding? Int. J. Obes. 41, 1131–1140 (2017). 10.1038/ijo.2017.74 28321131

[22] van der Hoek GA, Cooper SJ, The selective dopamine uptake inhibitor GBR 12909: Its effects on the microstructure of feeding in rats. Pharmacol. Biochem. Behav. 48, 135–140 (1994). 8029284 10.1016/0091-3057(94)90509-6

[23] West KS, Lawson V, Swanson AM, Dunigan AI, Roseberry AG, Amphetamine Dose-Dependently Decreases and Increases Binge Intake of Fat and Sucrose Independent of Sex. Obesity (Silver Spring) 27, 1874–1882 (2019). 31562706 10.1002/oby.22636 PMC6832849

[24] Tan B. , Drugs of abuse hijack a mesolimbic pathway that processes homeostatic need. Science 384, eadk6742 (2024). 10.1126/science.adk6742 PMC11077477 38669575

[25] Steinberg EE , A causal link between prediction errors, dopamine neurons and learning. Nat. Neurosci. 16, 966–973 (2013). 23708143 10.1038/nn.3413 PMC3705924

[26] Schultz W, Dayan P, Montague PR, A neural substrate of prediction and reward. Science 275, 1593–1599 (1997). 9054347 10.1126/science.275.5306.1593

[27] Mitchell FR, Garret DS, Paul EMP, Wightman RM, Regina MC, Dopamine Operates as a Subsecond Modulator of Food Seeking. The Journal of Neuroscience 24, 1265 (2004). 14960596 10.1523/JNEUROSCI.3823-03.2004 PMC6730321

[28] Grove JCR , Dopamine subsystems that track internal states. Nature 608, 374–380 (2022). 35831501 10.1038/s41586-022-04954-0 PMC9365689

[29] Berridge KC, Food reward: brain substrates of wanting and liking. Neurosci. Biobehav. Rev. 20, 1–25 (1996). 8622814 10.1016/0149-7634(95)00033-b

[30] Berridge KC, From prediction error to incentive salience: mesolimbic computation of reward motivation. Eur. J. Neurosci. 35, 1124–1143 (2012). 22487042 10.1111/j.1460-9568.2012.07990.x PMC3325516

[31] Gong R, Xu S, Hermundstad A, Yu Y, Sternson SM, Hindbrain Double-Negative Feedback Mediates Palatability-Guided Food and Water Consumption. Cell 182, 1589–1605 e1522 (2020). 32841600 10.1016/j.cell.2020.07.031

[32] Li MM , The Paraventricular Hypothalamus Regulates Satiety and Prevents Obesity via Two Genetically Distinct Circuits. Neuron, (2019). 10.1016/j.neuron.2019.02.028 PMC6508999 30879785

[33] Marino RAM , Control of food approach and eating by a GABAergic projection from lateral hypothalamus to dorsal pons. Proc. Natl. Acad. Sci. U. S. A. 117, 8611–8615 (2020). 32229573 10.1073/pnas.1909340117 PMC7165479

[34] Shin JW, Geerling JC, Stein MK, Miller RL, Loewy AD, FoxP2 brainstem neurons project to sodium appetite regulatory sites. J. Chem. Neuroanat. 42, 1–23 (2011). 21605659 10.1016/j.jchemneu.2011.05.003 PMC3148274

[35] Taha SA, Katsuura Y, Noorvash D, Seroussi A, Fields HL, Convergent, not serial, striatal and pallidal circuits regulate opioid-induced food intake. Neuroscience 161, 718–733 (2009). 19336249 10.1016/j.neuroscience.2009.03.057 PMC2699890

[36] Wassum KM, Ostlund SB, Maidment NT, Balleine BW, Distinct opioid circuits determine the palatability and the desirability of rewarding events. Proc. Natl. Acad. Sci. U. S. A. 106, 12512–12517 (2009). 19597155 10.1073/pnas.0905874106 PMC2718390

[37] Davis JD, The effectiveness of some sugars in stimulating licking behavior in the rat. Physiol. Behav. 11, 39–45 (1973). 4732426 10.1016/0031-9384(73)90120-0

[38] Davis JD, Smith GP, Analysis of the microstructure of the rhythmic tongue movements of rats ingesting maltose and sucrose solutions. Behav. Neurosci. 106, 217–228 (1992). 1554433

[39] Davis JD, Perez MC, Food deprivation- and palatability-induced microstructural changes in ingestive behavior. Am. J. Physiol. 264, R97–103 (1993). 8430892 10.1152/ajpregu.1993.264.1.R97

[40] Naneix F, Peters KZ, McCutcheon JE, Investigating the Effect of Physiological Need States on Palatability and Motivation Using Microstructural Analysis of Licking. Neuroscience 447, 155–166 (2020). 31682949 10.1016/j.neuroscience.2019.10.036

[41] Chow BY , High-performance genetically targetable optical neural silencing by light-driven proton pumps. Nature 463, 98–102 (2010). 20054397 10.1038/nature08652 PMC2939492

[42] Han X. , A high-light sensitivity optical neural silencer: development and application to optogenetic control of non-human primate cortex. Frontiers in systems neuroscience 5, 18 (2011). 21811444 10.3389/fnsys.2011.00018 PMC3082132

[43] Leib DE , The Forebrain Thirst Circuit Drives Drinking through Negative Reinforcement. Neuron 96, 1272–1281.e1274 (2017). 29268095 10.1016/j.neuron.2017.11.041 PMC5940335

[44] Libbrecht S, Van den Haute C, Malinouskaya L, Gijsbers R, Baekelandt V, Evaluation of WGA-Cre-dependent topological transgene expression in the rodent brain. Brain structure & function 222, 717–733 (2017). 27259586 10.1007/s00429-016-1241-x

[45] Gradinaru V. , Molecular and cellular approaches for diversifying and extending optogenetics. Cell 141, 154–165 (2010). 20303157 10.1016/j.cell.2010.02.037 PMC4160532

[46] Tan KR , GABA neurons of the VTA drive conditioned place aversion. Neuron 73, 1173–1183 (2012). 22445344 10.1016/j.neuron.2012.02.015 PMC6690362

[47] van Zessen R, Phillips JL, Budygin EA, Stuber GD, Activation of VTA GABA neurons disrupts reward consumption. Neuron 73, 1184–1194 (2012). 22445345 10.1016/j.neuron.2012.02.016 PMC3314244

[48] Klapoetke NC , Independent optical excitation of distinct neural populations. Nat Methods 11, 338–346 (2014). 24509633 10.1038/nmeth.2836 PMC3943671

[49] Zhang Y. , Fast and sensitive GCaMP calcium indicators for imaging neural populations. 2021.2011.2008.467793 (2021). 10.1038/s41586-023-05828-9 PMC10060165 36922596

[50] Sun F. , Next-generation GRAB sensors for monitoring dopaminergic activity in vivo. Nat. Meth. 17, 1156–1166 (2020). 10.1038/s41592-020-00981-9 PMC7648260 33087905

[51] LaFosse PK , Bicistronic Expression of a High-Performance Calcium Indicator and Opsin for All-Optical Stimulation and Imaging at Cellular Resolution. eNeuro 10, (2023). 10.1523/ENEURO.0378-22.2023 PMC10062490 36858826

[52] Yeomans MR, Morris J, Armitage RM, Hedonic contrast and the short-term stimulation of appetite. Appetite 155, 104849 (2020). 10.1016/j.appet.2020.104849 32828909

[53] Dove WF, The relative nature of human preference: with an example in the palatability of different varieties of sweet corn. J. Comp. Psychol. 35, 219–226 (1943).

[54] Lee SJ , Cell-type-specific asynchronous modulation of PKA by dopamine in learning. Nature 590, 451–456 (2021). 33361810 10.1038/s41586-020-03050-5 PMC7889726

[55] Coddington LT, Lindo SE, Dudman JT, Mesolimbic dopamine adapts the rate of learning from action. Nature 614, 294–302 (2023). 36653450 10.1038/s41586-022-05614-z PMC9908546

[56] Wiesenfeld Z, Halpern BP, Tapper DN, Licking behavior: evidence of hypoglossal oscillator. Science 196, 1122–1124 (1977). 558653 10.1126/science.558653

[57] Chuong AS , Noninvasive optical inhibition with a red-shifted microbial rhodopsin. Nat Neurosci 17, 1123–1129 (2014). 24997763 10.1038/nn.3752 PMC4184214

[58] Gabery S. , Semaglutide lowers body weight in rodents via distributed neural pathways. JCI insight 5, (2020). 10.1172/jci.insight.133429 PMC7213778 32213703

[59] Lee K. , Temporally restricted dopaminergic control of reward-conditioned movements. Nat Neurosci 23, 209–216 (2020). 31932769 10.1038/s41593-019-0567-0 PMC7007363

[60] Fiorillo CD, Newsome WT, Schultz W, The temporal precision of reward prediction in dopamine neurons. Nat. Neurosci. 11, 966–973 (2008). 18660807 10.1038/nn.2159

[61] Low AYT , Reverse-translational identification of a cerebellar satiation network. Nature 600, 269–273 (2021). 34789878 10.1038/s41586-021-04143-5 PMC8665128

[62] Rolls ET, Sienkiewicz ZJ, Yaxley S, Hunger Modulates the Responses to Gustatory Stimuli of Single Neurons in the Caudolateral Orbitofrontal Cortex of the Macaque Monkey. Eur. J. Neurosci. 1, 53–60 (1989). 12106174 10.1111/j.1460-9568.1989.tb00774.x

[63] Berridge KC, ‘Liking’ and ‘wanting’ food rewards: Brain substrates and roles in eating disorders. Physiol. Behav. 97, 537–550 (2009). 19336238 10.1016/j.physbeh.2009.02.044 PMC2717031

[64] Rolls ET, The orbitofrontal cortex, food reward, body weight and obesity. Social cognitive and affective neuroscience 18, (2021). 10.1093/scan/nsab044 PMC9997078 33830272

[65] Grill HJ, Norgren R, The taste reactivity test. I. Mimetic responses to gustatory stimuli in neurologically normal rats. Brain Res. 143, 263–279 (1978). 630409 10.1016/0006-8993(78)90568-1

[66] Mazzone CM , High-fat food biases hypothalamic and mesolimbic expression of consummatory drives. Nat. Neurosci. 23, 1253–1266 (2020). 32747789 10.1038/s41593-020-0684-9 PMC7529959

[67] Rossi MA, Stuber GD, Overlapping Brain Circuits for Homeostatic and Hedonic Feeding. Cell Metab. 27, 42–56 (2018). 29107504 10.1016/j.cmet.2017.09.021 PMC5762260

[68] Salamone John D., Correa M, The Mysterious Motivational Functions of Mesolimbic Dopamine. Neuron 76, 470–485 (2012). 23141060 10.1016/j.neuron.2012.10.021 PMC4450094

[69] Coddington LT, Dudman JT, The timing of action determines reward prediction signals in identified midbrain dopamine neurons. Nat. Neurosci. 21, 1563–1573 (2018). 30323275 10.1038/s41593-018-0245-7 PMC6226028

[70] Mendonça MD , Dopamine neuron activity encodes the length of upcoming contralateral movement sequences. Curr. Biol. 34, 1034–1047.e1034 (2024). 38377999 10.1016/j.cub.2024.01.067 PMC10931818

[71] da Silva JA, Tecuapetla F, Paixão V, Costa RM, Dopamine neuron activity before action initiation gates and invigorates future movements. Nature 554, 244–248 (2018). 29420469 10.1038/nature25457

[72] Coddington LT, Dudman JT, Learning from Action: Reconsidering Movement Signaling in Midbrain Dopamine Neuron Activity. Neuron 104, 63–77 (2019). 31600516 10.1016/j.neuron.2019.08.036

[73] Howe MW, Dombeck DA, Rapid signalling in distinct dopaminergic axons during locomotion and reward. Nature 535, 505–510 (2016). 27398617 10.1038/nature18942 PMC4970879

[74] O’Connor EC , Accumbal D1R Neurons Projecting to Lateral Hypothalamus Authorize Feeding. Neuron 88, 553–564 (2015). 26593092 10.1016/j.neuron.2015.09.038

[75] Jennings JH , Visualizing hypothalamic network dynamics for appetitive and consummatory behaviors. Cell 160, 516–527 (2015). 25635459 10.1016/j.cell.2014.12.026 PMC4312416

[76] Nieh EH , Decoding neural circuits that control compulsive sucrose seeking. Cell 160, 528–541 (2015). 25635460 10.1016/j.cell.2015.01.003 PMC4312417

[77] Navarro M. , Lateral Hypothalamus GABAergic Neurons Modulate Consummatory Behaviors Regardless of the Caloric Content or Biological Relevance of the Consumed Stimuli. Neuropsychopharmacology 41, 1505–1512 (2016). 26442599 10.1038/npp.2015.304 PMC4832010

[78] Woods JW, BEHAVIOR OF CHRONIC DECEREBRATE RATS. J Neurophysiol 27, 635–644 (1964). 14194963 10.1152/jn.1964.27.4.635

[79] Grill HJ, Norgren R, The taste reactivity test. II. Mimetic responses to gustatory stimuli in chronic thalamic and chronic decerebrate rats. Brain Res. 143, 281–297 (1978). 630410 10.1016/0006-8993(78)90569-3

[80] Kooij KL , GLP-1 receptor agonist semaglutide reduces appetite while increasing dopamine reward signaling. Neuroscience Applied 3, 103925 (2024). 10.1016/j.nsa.2023.103925 PMC12244221 40656110

[81] Coppin G. , A randomized controlled trial investigating the effect of liraglutide on self-reported liking and neural responses to food stimuli in participants with obesity. Int J Obes (Lond) 47, 1224–1231 (2023). 37626125 10.1038/s41366-023-01370-w PMC10663148

[82] Kadouh H. , GLP-1 Analog Modulates Appetite, Taste Preference, Gut Hormones, and Regional Body Fat Stores in Adults with Obesity. The Journal of Clinical Endocrinology & Metabolism 105, 1552–1563 (2019). 10.1210/clinem/dgz140 PMC7105351 31665455

[83] Wharton S. , Two-year effect of semaglutide 2.4 mg on control of eating in adults with overweight/obesity: STEP 5. Obesity (Silver Spring) 31, 703–715 (2023). 36655300 10.1002/oby.23673

[84] Blundell J. , Effects of once-weekly semaglutide on appetite, energy intake, control of eating, food preference and body weight in subjects with obesity. Diabetes, obesity & metabolism 19, 1242–1251 (2017). 10.1111/dom.12932 PMC5573908 28266779

[85] Cawthon CR , Chronic Semaglutide Treatment in Rats Leads to Daily Excessive Concentration-Dependent Sucrose Intake. Journal of the Endocrine Society 7, bvad074 (2023). 10.1210/jendso/bvad074 PMC10306276 37388574

[86] Farr OM , Longer-term liraglutide administration at the highest dose approved for obesity increases reward-related orbitofrontal cortex activation in response to food cues: Implications for plateauing weight loss in response to anti-obesity therapies. Diabetes, obesity & metabolism 21, 2459–2464 (2019). 10.1111/dom.13827 PMC6800581 31282006

[87] Boyden ES, Zhang F, Bamberg E, Nagel G, Deisseroth K, Millisecond-timescale, genetically targeted optical control of neural activity. Nat. Neurosci. 8, 1263–1268 (2005). 16116447 10.1038/nn1525

