# Supplementary material for: Hedonic eating is controlled by dopamine neurons that oppose GLP-1R satiety
Source: Science. Author manuscript; Available in PMC 2025 Apr 19. (PMC12009138; doi:10.1126/science.adt0773)
Supplement: Supplementary Materials and Table S1 [file NIHMS2070302-supplement-Supplementary_Materials_and_Table_S1.pdf]

Science
AAAS

# Supplementary Materials for

**Hedonic eating is controlled by dopamine neurons that oppose GLP-1R satiety**

Zhenggang Zhu, Rong Gong, Vicente Rodriguez, Kathleen T. Quach, Xinyu Chen, Scott M. Sternson*

Corresponding author: ssternson@health.ucsd.edu

**The PDF file includes:**

Figs. S1 to S20
Table S1

**Other Supplementary Materials for this Manuscript includes the following:**

MDAR Reproducibility Checklist

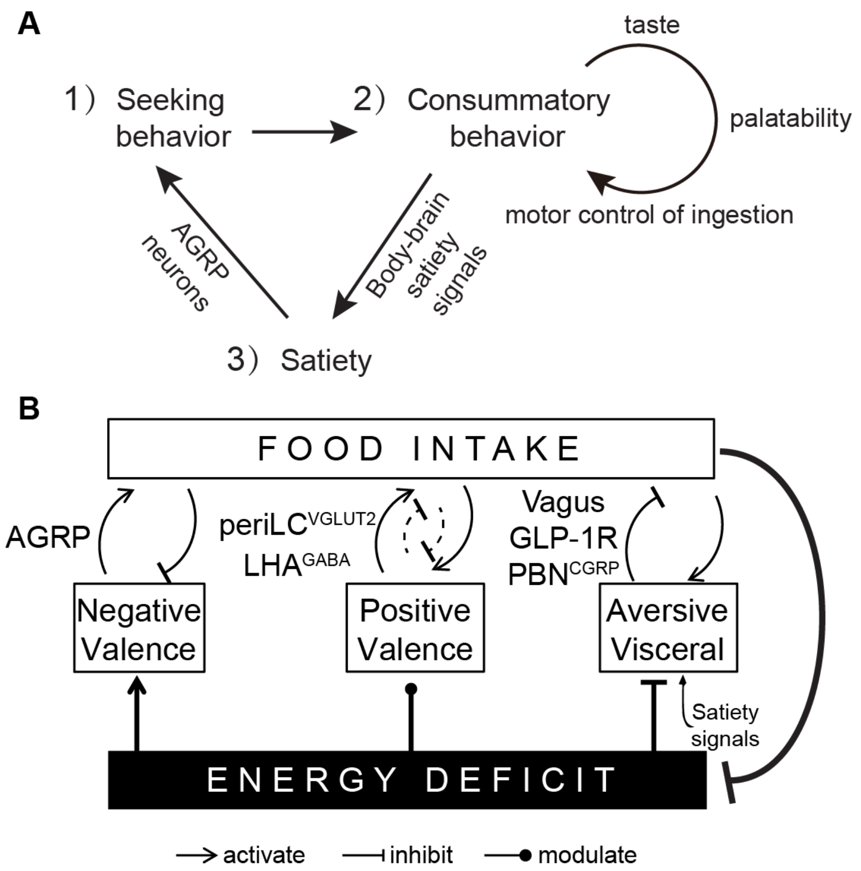


**Fig. S1. Three phases of feeding behavior**. (A) Feeding behavior starts with food-seeking. Next, food consumption is initiated and is sustained by feedback from palatable taste. Satiety pathways from the body respond to ingested nutrients, signal to the brain, and terminate consumption. (B) The phases of feeding behavior are mediated by different pathways with distinct neural dynamics and motivational characteristics. First, food-seeking systems have been identified that are activated by energy deficit and promote food-seeking but are inhibited by cues that predict food and have low activity during food ingestion, e.g. Agouti-related protein (AGRP) neurons. Second, neurons engaged during consummatory behavior can also promote consumption, have positive valence, and are modulated by energy deficit. However, energy deficit is not required for these consummatory pathways because they are also engaged by palatable food intake. Examples of consummatory control neurons include lateral hypothalamic area (LHA$^{GABA}$) neurons that show positive feedback between food ingestion and their activity or periLC$^{VGLUT2}$ neurons that show a double negative feedback relationship where food ingestion inhibits these neurons, which correspondingly promotes further food intake and is rewarding. Third, neurons responsive to bodily signals mediated by the vagus nerve, circulating hormones, GLP-1R agonists, and parabrachial nucleus Calcitonin gene-related peptide (PBN$^{CGRP}$) neurons induce satiety to terminate a meal. These pathways often show a progressive rise in activity during food intake, and some have negative valence when activated. Interactions between these systems exist but are not shown here.

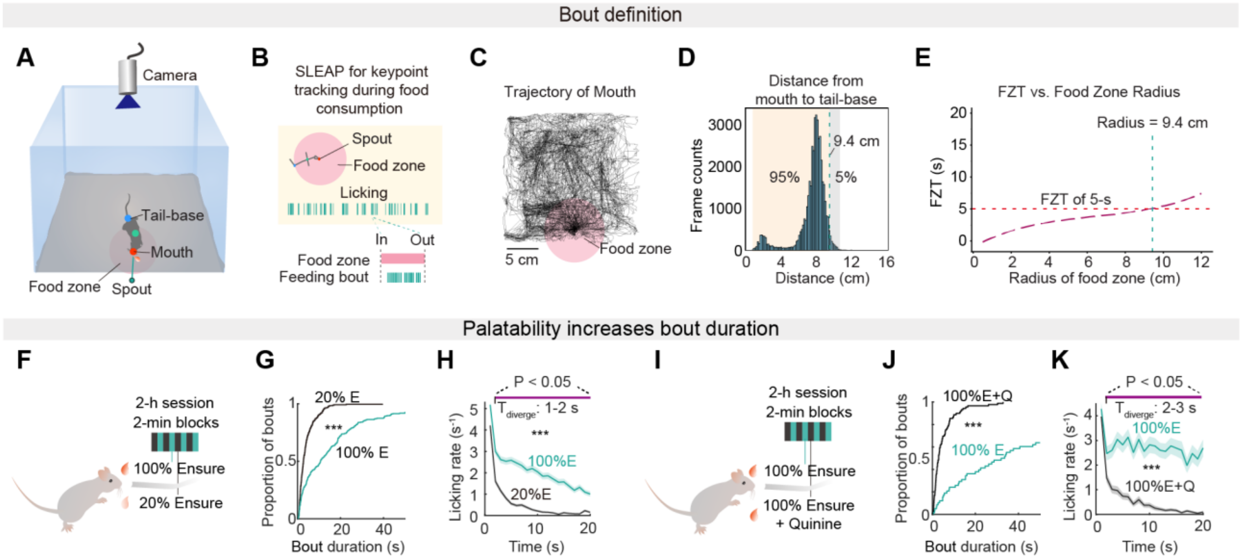


**Fig. S2. Bout duration is based on sequential licking events in the food zone and is increased by higher palatability food.** (A, B) A feeding bout is a sequence of licks that is terminated by an interlick interval (ILI) greater than a threshold value. We defined a bout ILI greater than the shortest 10% of times in which the mouse was in the food zone (FZ), which was based on the notion that a feeding bout ended when the mouse leaves the FZ. We defined the FZ as one body length (mouth to tail-base) from the lick spout. To empirically determine the FZ size and the shortest 10% of FZ time (FZT), we used an experimental setup with a camera capturing video for tracking key points, including the mouth, tail-base, and body position relative to the lick spout at the center of the FZ (A). (B) Raster of lick events when a mouse enters the FZ. Key points indicate mouse position and the pink area represents the FZ boundary. (C) Representative data showing the position of the mouth, which was tracked during one session of palatable food consumption. Scale bar: 5 cm. (D) The mean body length was calculated from video tracking as the 95% probability distance between the mouth and tail-base during a feeding session, which was 9.4 cm (n = 4 mice). (E) From the tracking data, we calculated the shortest 10% of FZT values based on different radii defining the FZ. For the FZ radius defined by body length (9.4 cm), the shortest 10% of FZT values was 5-s, which was used as the inter-lick interval to define the bout threshold. (F, I) Feeding bout analysis comparing the consumption of higher and lower palatability food (100% Ensure *vs* 20% Ensure (F-H, n = 11 mice) or 100% Ensure with Quinine (I-K, n = 7 mice) available in alternating 2-minute blocks (teal and black blocks, respectively). (G-K) Higher palatability food leads to a greater proportion of long bouts (G, J), with a higher licking rate compared to lower palatability food after 1-s (H) or after 2-s of licking onset (K). ***p<0.001. Statistical details are in Table S1.

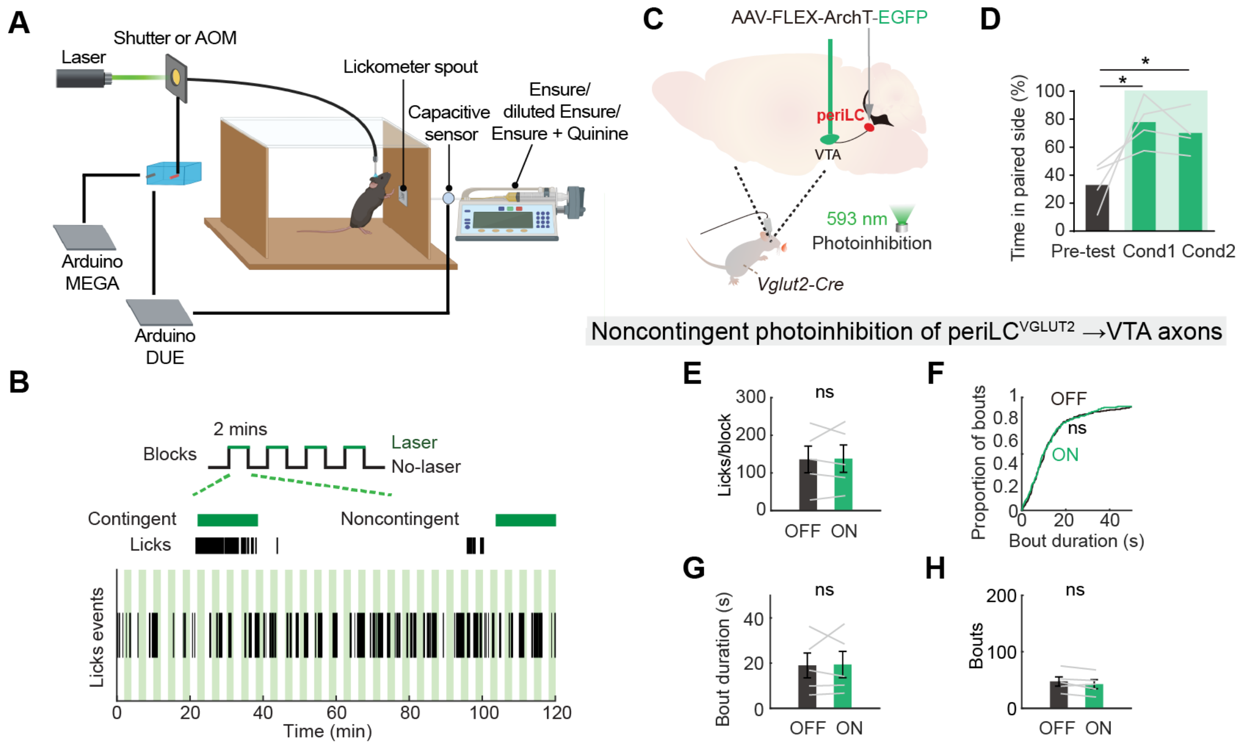


**Fig. S3. Photoinhibition of periLC$^{VGLUT2}$ axon projections to the VTA conditions place preference but noncontingent photoinhibition does not affect food intake.** (A) Schematic of lick-triggered optogenetics apparatus. A capacitive lick detector on the liquid food spout registers licks that activate a syringe pump to deliver food as well as trigger a pulse generator program to deliver laser light in optogenetic experiments by controlling a shutter or an acoustic-optic modulator (AOM). (B) Interleaved block structure for closed-loop lick-contingent and open-loop lick-noncontingent perturbation and example lick events (black ticks) in one session. For lick-contingent sessions, licking the food spout delivers food and concurrently triggers the laser in laser-ON blocks (green, 2-min), and for alternating laser-OFF blocks (white, 2-min) licking delivers food but no laser. The same blockwise session structure is used for lick-noncontingent sessions but the optogenetic stimulation pattern from the prior lick-contingent session is used independently of the mouse licking behavior. (C) Schematic for photoinhibition of periLC$^{VGLUT2}$ axon projections to downstream VTA during place preference test and feeding in *Vglut2-IRES-Cre* mice. (D) Photoinhibition of periLC$^{VGLUT2}$ →VTA axon projections conditioned place preference (rmANOVA, n = 4 mice). (E-H) Noncontingent photoinhibition of periLC$^{VGLUT2}$ →VTA axon projections did not significantly affect food consumption (E), bout duration (F-G), or bout number (H) (KS-test and paired t-test, n = 5 mice). Data are represented as mean ± SEM. ns p>0.05, *p<0.05. Statistical details are in Table S1.

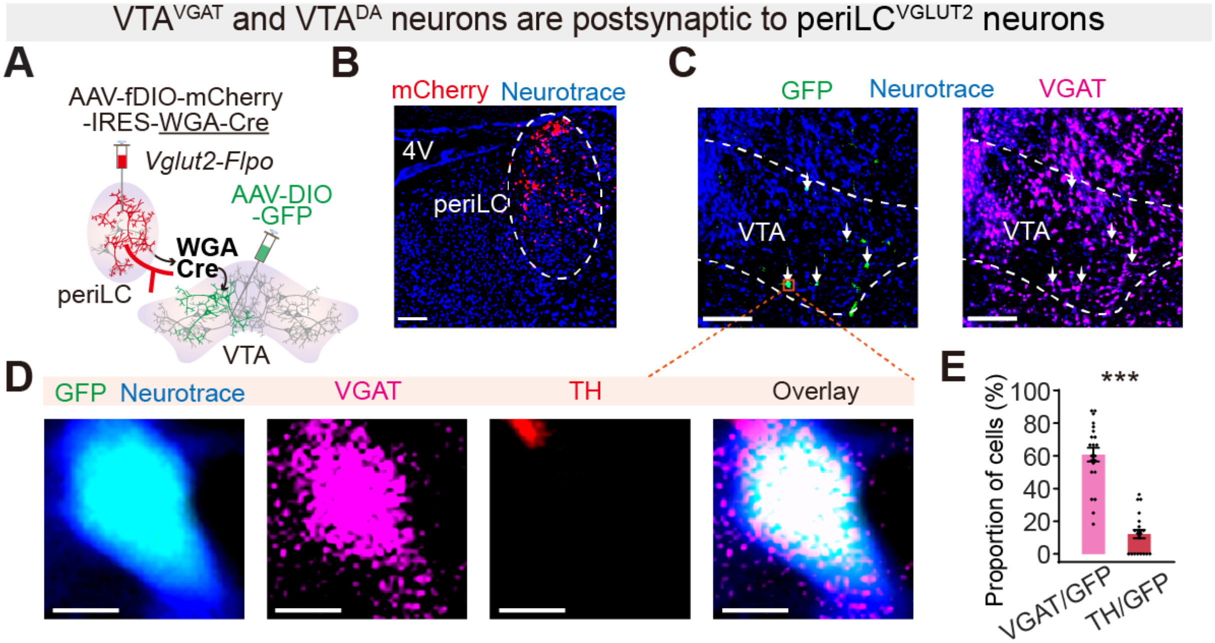


**Fig. S4. Intersectional anterograde tracing of periLC$^{VGLUT2}$ neurons to VTA$^{VGAT}$ and VTA$^{DA}$ neurons.** (A) Schematic of the experimental strategy for intersectional anterograde transsynaptic labeling of VTA neurons downstream of periLC$^{VGLUT2}$ neurons. AAV-fDIO-mCherry-IRES-WGA-Cre was injected into the periLC region of *Vglut2-IRES-Flpo* mice, along with AAV-DIO-GFP injected into the VTA. (B) Representative image showing mCherry expression (co-expressed fluorescent protein marker for transduction with WGA-Cre) in the periLC region. Dashed circle indicates periLC, and 4V marks the fourth ventricle. Scale bar: 100 µm. (C) Coronal sections of the VTA show GFP (green, left), Neurotrace (blue) staining, and VGAT (magenta, right) labeling. Arrows indicate GFP+ VGAT+ neurons, demonstrating periLC$^{VGLUT2}$ input to GABAergic neurons. Scale bar: 200 µm. (D) Insets in (C) show a GFP+/VGAT+ neuron that is TH-negative. Scale bars: 5 µm. (E) Quantification of GFP labeling in VGAT+ (GABAergic) and TH+ (dopaminergic) neurons in the VTA (t-test, n = 22 VTA sections from 2 mice). Data are represented as mean ± SEM. ***$p<0.001$. Statistical details are in Table S1.

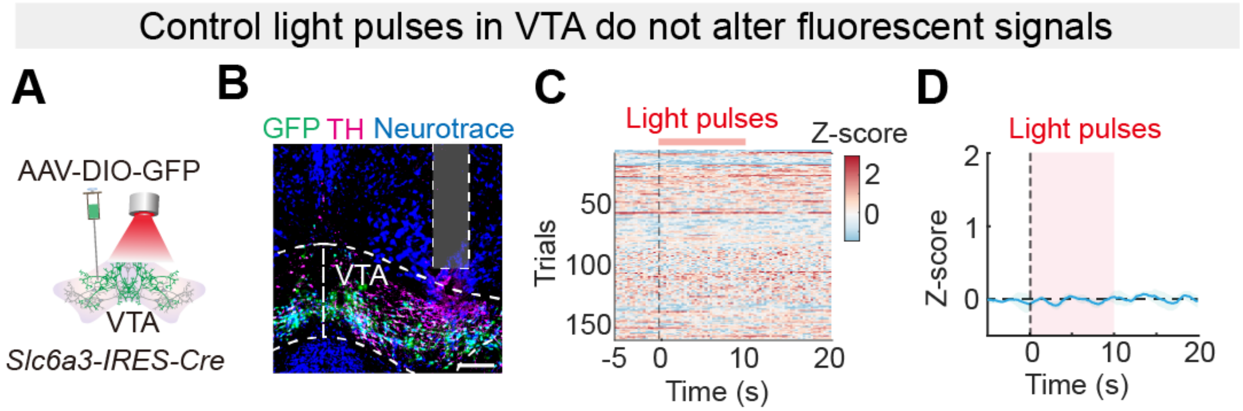


**Fig. S5. Negative control experiments for photostimulation during photometry recordings in the VTA.** (A) Photometry setup for monitoring GFP fluorescence in $VTA^{DA}$ neurons during 635 nm light pulses in the VTA (*Slc6a3-IRES-Cre* mice). (B) Expression of GFP (green) and TH (magenta) within VTA. Scale bar, 200 µm. (C) Photometry recordings show the absence of responses in GFP-expressing neurons during 10-s light pulses. (D) GFP negative control mean responses (blue) during 10-s light pulses (n = 2 mice). Data are represented as mean ± SEM.

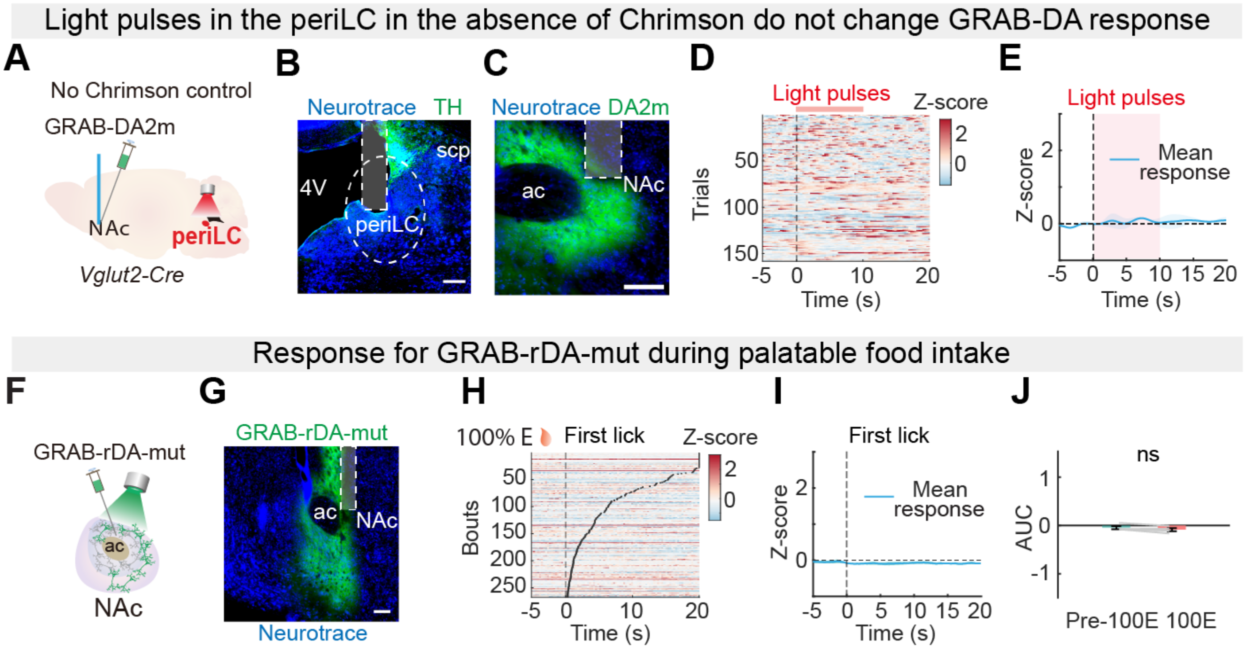


**Fig. S6. Negative controls for GRAB-DA experiments in NAc during palatable food consumption**. (A) Photometry setup for monitoring NAc dopamine during light pulses in the periLC without Chrimson in periLC$^{VGLUT2}$ neurons (n = 2, *Vglut2-IRES-Cre* mice). (B, C) Fiber implantation (grey shading) over periLC and expression of TH (green) in the LC (B) and expression of GRAB-DA2m in NAc (C). Scale bars, 200 µm. (D) NAc GRAB-DA responses during delivery of 10-s light pulses. (E) GRAB-DA response was not significantly different during 10-s light pulses in the periLC. (F) Photometry setup recording the GRAB-rDA-mut control during consumption of 100% Ensure. (G) Expression of GRAB-rDA-mut (green) within NAc. Scale bar, 200 µm. (H) GRAB-rDA-mut control responses during consumption of 100% Ensure. (I, J) GRAB-rDA-mut control mean responses (blue) (I), showed no significant change of AUC during consumption of 100% Ensure (J, paired t-test, n = 4 C57BL6/J mice). Data are represented as mean ± SEM. ns p>0.05. Statistical details are provided in Table S1.

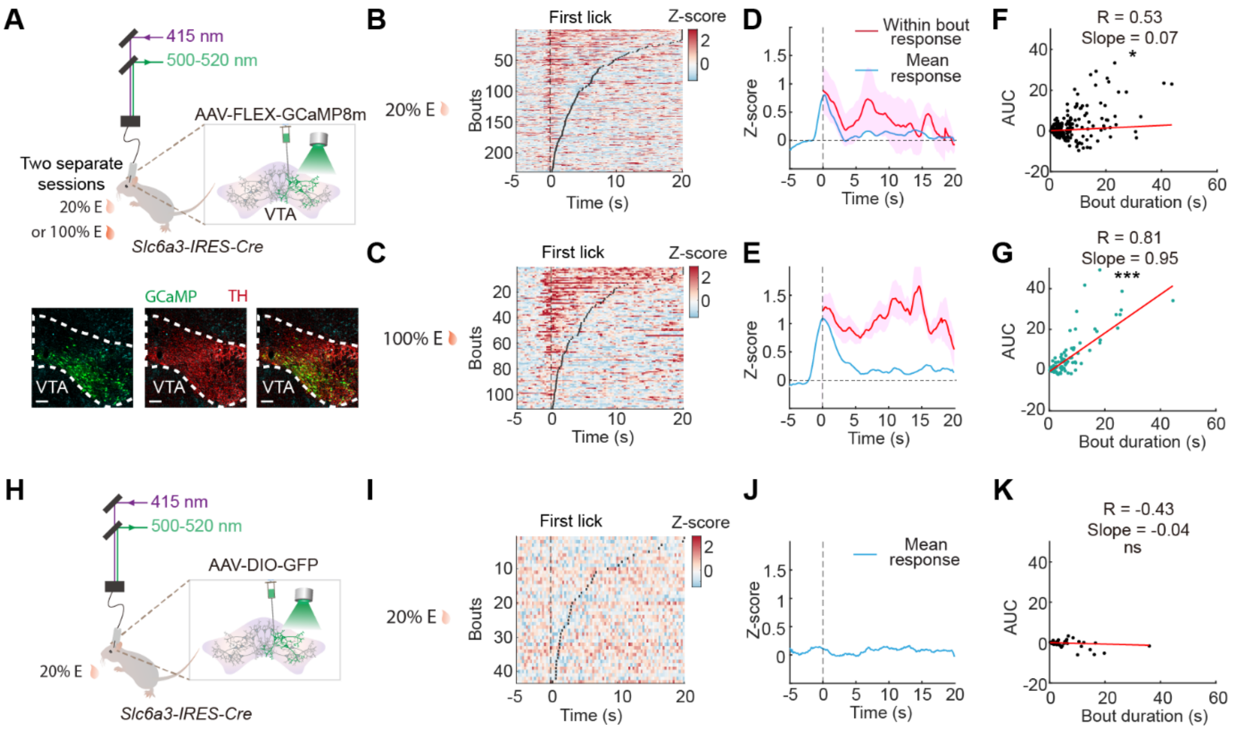


**Fig. S7. Reproducibility of VTA$^{DA}$ neuron calcium dynamics relationship with food consumption duration, effects of palatability, and GFP control experiment**. (A) Upper panel, photometry setup showing VTA$^{DA}$ neuron dynamics during consumption of 20% or 100% Ensure during separate sessions (*Slc6a3-IRES-Cre* mice). Bottom panels, viral expression of GCaMP8m in VTA (green, left), anti-TH (red, center), and overlaid images (right). E: Ensure. Scale bars, 100 µm. (B-E) GCaMP8m responses during consumption of 20% (B, D) and 100% Ensure (C, E). (D, E) GCaMP8m mean responses (blue) and variable-length time mean response across all bouts (magenta) during consumption of 20% Ensure and 100% Ensure. (F-G) Regression of GCaMP8m AUC with bout duration during consumption of 20% Ensure (F) and 100% of Ensure (G) (n = 4 mice). (H) Photometry setup recording the GFP control of VTA$^{DA}$ neuron dynamics during consumption of 20% or 100% Ensure (n = 3 *Slc6a3-IRES-Cre* mice). (I) GFP control responses during consumption of 20% Ensure. (J) GFP control mean responses (blue) during consumption of 20% Ensure. (K) Regression of GFP control AUC with bout duration during consumption of 20% Ensure (n = 3 mice). Data are represented as mean ± SEM. ns $p>0.05$, *$p<0.05$, ***$p<0.001$. Statistical details are provided in Table S1.

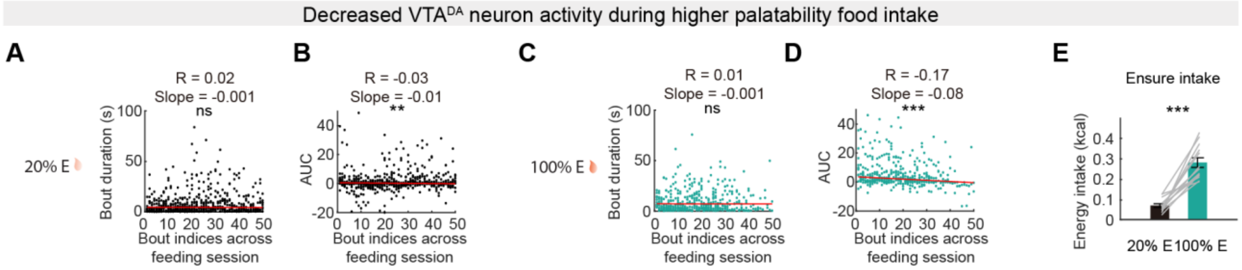


**Fig. S8. Across-session bout duration and VTA$^{DA}$ neuron dynamics**. (A-D) Linear regression of bout duration (A, C) or AUC of GCaMP8s responses (B, D) with the bout indices across each separate feeding session during consumption of 20% Ensure (A, B) or 100% Ensure (C, D) (n = 13 mice). E: Ensure. (E) Energy intake for 20% and 100% Ensure sessions (n = 13 mice). ns p>0.05, **p<0.01, ***p<0.001. Statistical details are provided in Table S1.

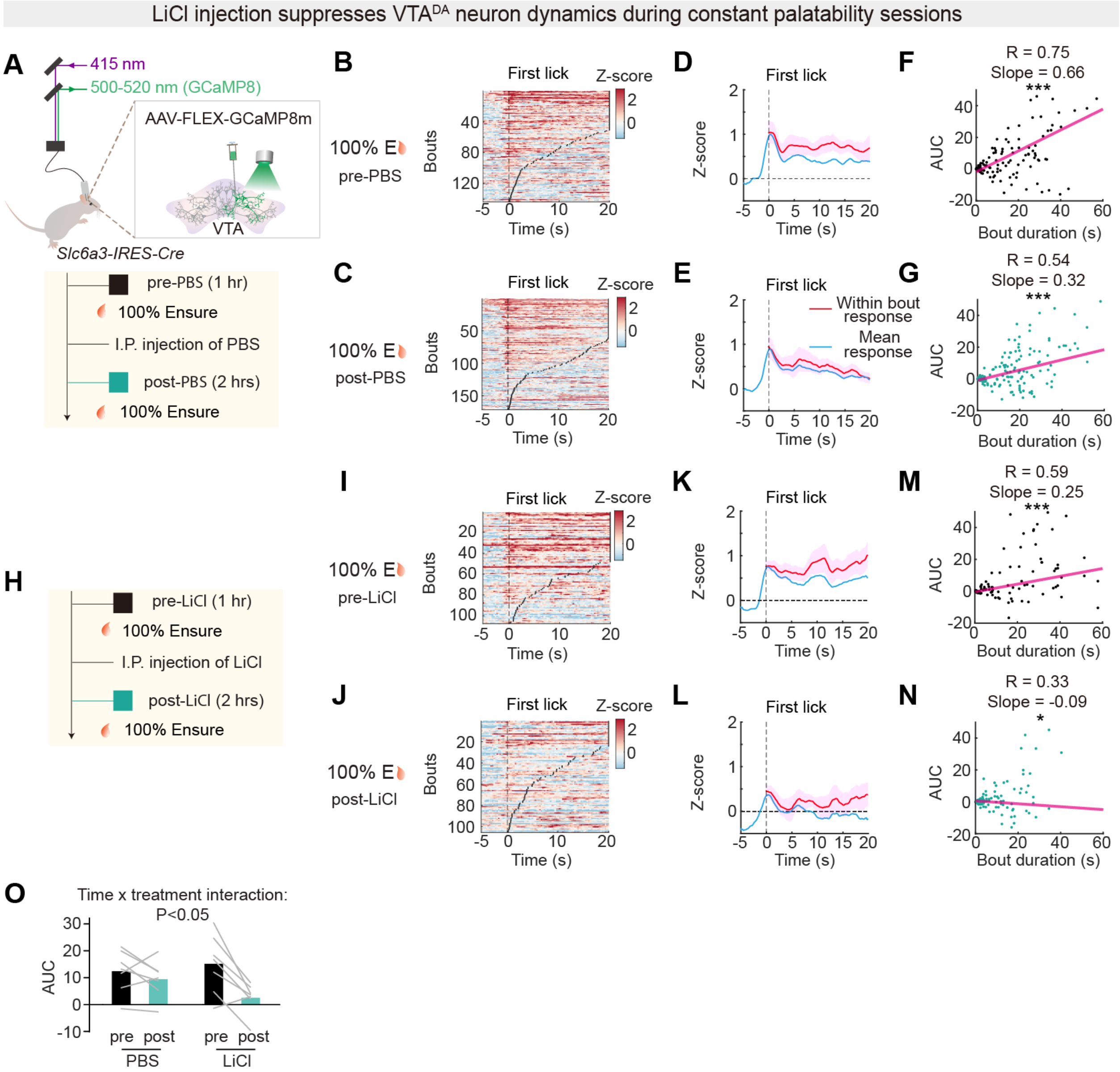


**Fig. S9. LiCl suppresses VTA$^{DA}$ neuron dynamics during food consumption**. (A) Photometry setup for monitoring VTA$^{DA}$ neuron dynamics during consumption of 100% Ensure before or after PBS injection (*Slc6a3-IRES-Cre* mice). (B-E) GCaMP8m responses during consumption of 100% Ensure before (B, D) or after PBS injection (C, E). (D, E) GCaMP8m mean responses (blue) and variable-length time mean response across all bouts (magenta) during consumption of 100% Ensure before or after PBS injection. (F-G) Regression of GCaMP8m AUC with bout duration during consumption of 100% Ensure before (F) or after (G) PBS injection (n = 7 mice). (H) Schematic of recording VTA$^{DA}$ neuron dynamics during consumption of 100% Ensure before or after LiCl injection during separate sessions (n = 7 *Slc6a3-IRES-Cre* mice). (I-L) GCaMP8m responses during consumption of 100% Ensure before (I, K) or after LiCl injection (J, L). (K, L) GCaMP8m mean responses (blue) and variable-length time mean response across all bouts (magenta) during consumption of 100% Ensure before (K) or after (L) LiCl injection. (M, N) Regression of GCaMP8m AUC with bout duration during consumption of 100% Ensure before (M) or after (N) LiCl injection (n = 7 mice). (O) VTA$^{DA}$ neuron calcium dynamics show a lower

AUC for palatable food after LiCl injection (rmANOVA, n = 7 mice). Data are represented as mean ± SEM. ns $p>0.05$, *$p<0.05$, ***$p<0.001$. Statistical details are in Table S1.

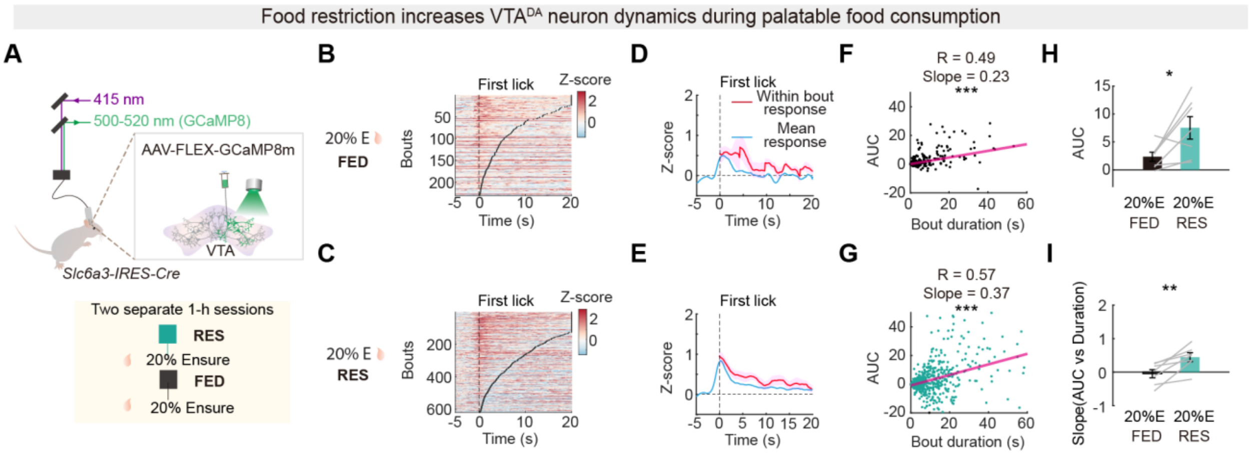


**Fig. S10. Food restriction increases VTA$^{DA}$ neuron response during palatable food consumption**. (A) Photometry setup showing VTA$^{DA}$ neuron dynamics during consumption of 20% Ensure in *ad-libitum* fed (FED) or food-restricted (RES) mice during separate sessions (*Slc6a3-IRES-Cre* mice). (B-E) GCaMP8m responses during consumption of 20% Ensure in FED (B, D) and RES mice (C, E). (D, E) GCaMP8m mean responses (blue) and variable-length time mean response across all bouts (magenta) during consumption of 20% Ensure in FED (D) or RES mice (E). (F-G) Regression of GCaMP8m AUC with bout duration during consumption of 20% Ensure in FED (F) and RES mice (G) (n = 7 mice). (H-I) VTA$^{DA}$ neuron dynamics show a significantly larger AUC (H) and a steeper slope of GCaMP8s AUC/Bout Duration (I) for palatable food in RES mice (paired t-test, n = 7 mice). Data are represented as mean ± SEM. *p<0.05, **p<0.01, ***p<0.001. Statistical details are provided in Table S1.

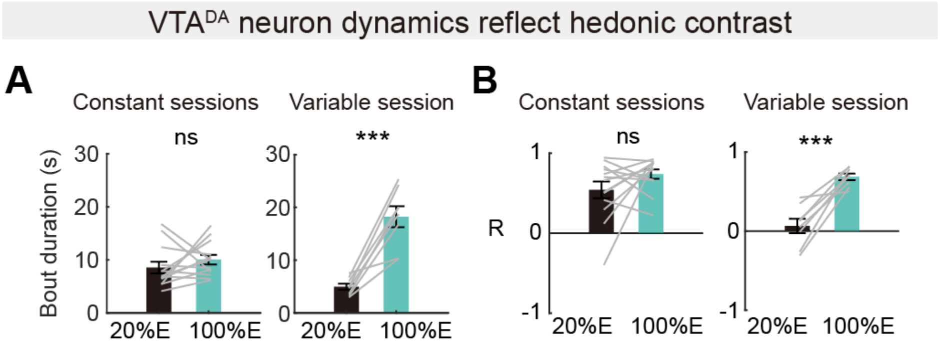


**Fig. S11. Across-session bout duration and VTA$^{DA}$ neuron dynamics**. (A) Bout durations for 20% and 100% Ensure in constant palatability sessions (n = 13 mice) and variable palatability sessions (n = 8 mice). (B) Pearson correlation coefficients (R) for regression of photometry AUC and bout duration in constant palatability sessions (n = 13 mice) and variable palatability sessions (n = 8 mice). Data are represented as mean ± SEM. ns p>0.05, ***p<0.001. Statistical details are in Table S1.

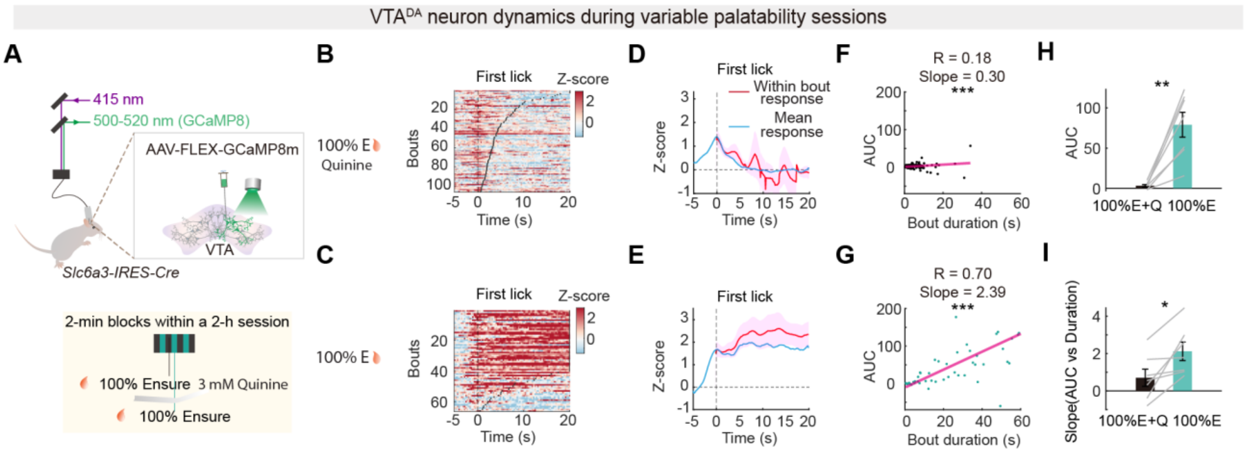


**Fig. S12. Hedonic contrast with quinine adulteration increases VTA$^{DA}$ neuron responses during consumption of higher palatability food with constant nutrient content**. (A) Photometry setup showing VTA$^{DA}$ neuron dynamics during consumption of 100% Ensure or 100% Ensure adulterated with Quinine during the same session (*Slc6a3-IRES-Cre* mice). (B-E) GCaMP8m responses during consumption of 100% Ensure with Quinine (B, D) and 100% Ensure (C, E). (D, E) GCaMP8m mean responses (blue) and variable-length time mean response across all bouts (magenta) during consumption of 100% Ensure with Quinine and 100% Ensure. (F-G) Regression of GCaMP8m AUC with bout duration during consumption of 100% Ensure with Quinine (F) and 100% of Ensure (G) (n = 7 mice). (H-I) VTA$^{DA}$ neuron dynamics show a larger AUC (H) and a steeper slope of GCaMP8m AUC/Bout Duration (I) for the higher palatability food lacking quinine (paired t-test, n = 7 mice). Data are represented as mean ± SEM. *p<0.05, **p<0.01, ***p<0.001. Statistical details are in Table S1.

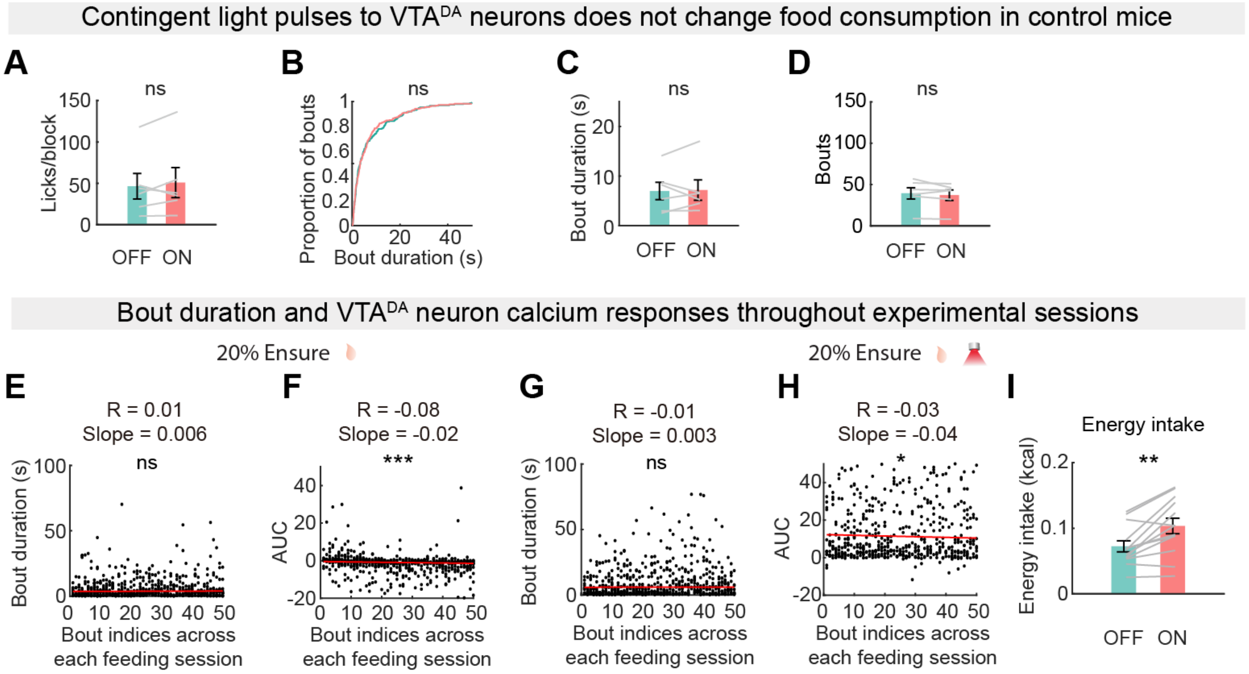


**Fig. S13. VTA$^{DA}$ neuron photostimulation control experiments and comparison of bout duration and neuron activity of each feeding session.** (A-D) Contingent light pulses to VTA$^{DA}$ neurons do not change consumption (A), bout duration, and bout number during laser-on blocks in GCaMP8m or GFP control mice (both lacking Chrimson in VTA$^{DA}$ neurons) (B-D, KS-test and paired t-test, n = 6). (E-H) Linear regression of bout duration (E, G) or GCaMP8s AUC (F, H) with the bout indices across each separate feeding session during consumption of 20% Ensure (E, F) or 20% Ensure with photometry-calibrated VTA$^{DA}$ neuron photostimulation (G, H) (n = 13 mice). (I) Energy intake for 20% and 20% Ensure with photometry-calibrated VTA$^{DA}$ neuron photostimulation sessions (n = 13 mice). Data are represented as mean ± SEM. ns p>0.05, *p<0.05, **p<0.01, ***p<0.001. Statistical details are provided in Table S1.

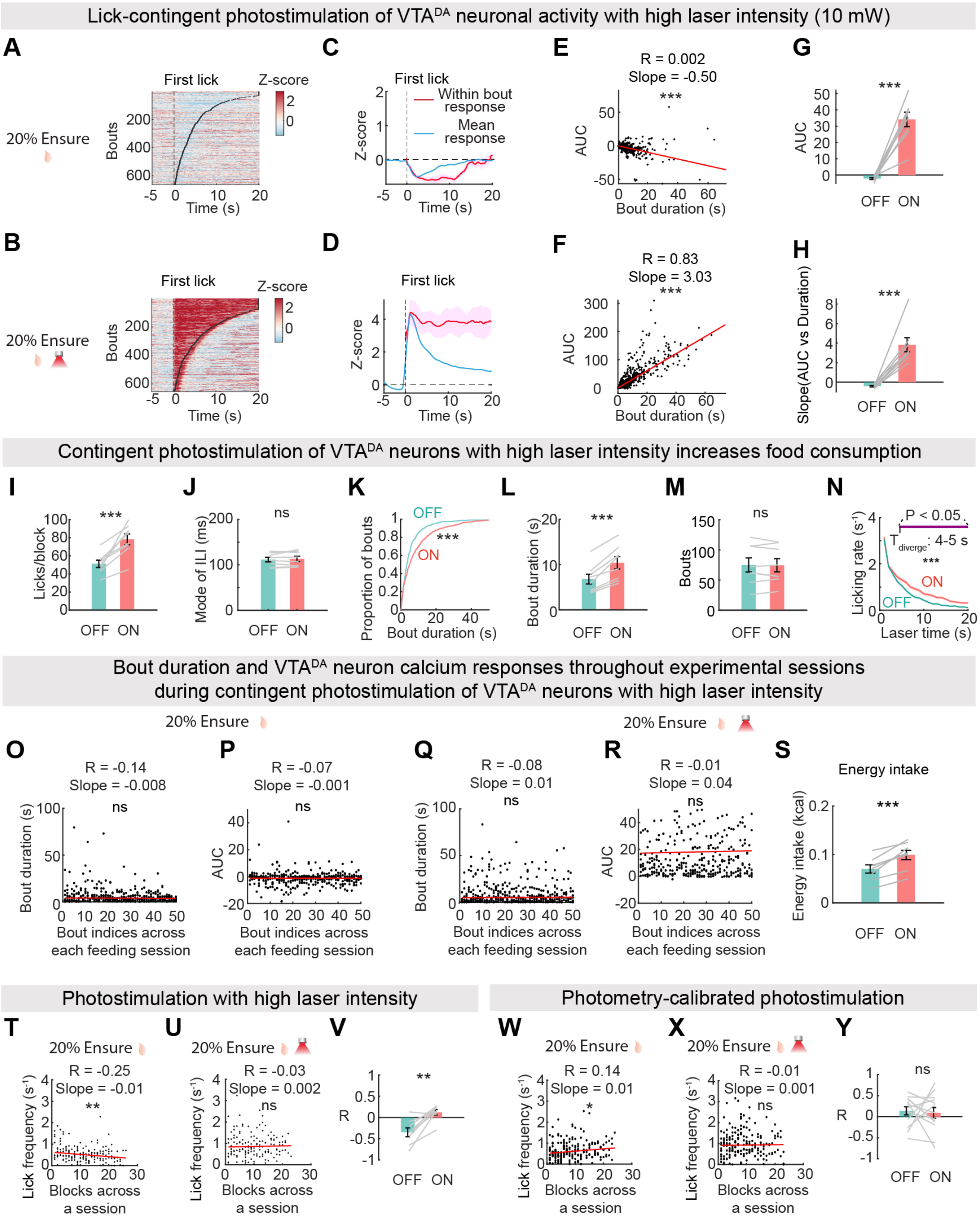


**Fig. S14. Photostimulation of VTA$^{DA}$ neuron dynamics with a higher laser intensity reinforces food consumption**. (A-D) GCaMP8s responses during consumption of 20% (A, C) and 20% Ensure with lick-contingent VTA$^{DA}$ neuron photostimulation with a higher laser intensity (10 mW) (B, D). (C, D) GCaMP8s mean responses (blue) and mean response within a bout (magenta) during consumption of 20% Ensure and 20% Ensure with contingent photostimulation with a higher laser intensity. (E-F) Regression of GCaMP8s AUC with bout duration during

consumption of 20% Ensure (E) and 20% Ensure with contingent photostimulation with a higher laser intensity (F) (n = 8 mice). (G-H) $VTA^{DA}$ neuron dynamics show a larger area under the curve (AUC) (G) and a steeper slope of GCaMP8s AUC/Bout Duration (H) for contingent photostimulation with a higher laser intensity (paired t-test, n = 8 mice). (I-N) Contingent photostimulation of $VTA^{DA}$ neurons with a higher laser intensity increases consumption (I-J), and bout duration but not bout number during laser-ON blocks (K-N) (negative binomial generalized linear mixed model, KS-test and paired t-test, n = 8). (O-R) Linear regression of bout duration (O, Q) or AUC of GCaMP8s responses (P, R) with the bout indices across each separate feeding session during consumption of 20% Ensure (O, P) or 20% Ensure with contingent photostimulation with a higher laser intensity (Q, R) (n = 8 mice). (S) Energy intake for 20% Ensure during ON and OFF blocks of $VTA^{DA}$ contingent photostimulation with a higher laser intensity (n = 8 mice). (T-V) Linear regression of mean lick frequency within the 2-minute blocks index across a session on laser-OFF (T) and laser-ON (U) periods during the photostimulation with high laser power and comparison of correlation coefficient in ON and OFF blocks (V, paired t-test, n = 8 mice). (W-Y) As for T-V with photometry-calibrated photostimulation (n = 13 mice). Data are represented as mean ± SEM. ns p>0.05, *p<0.05, **p<0.01, ***p<0.001. Statistical details are provided in Table S1.

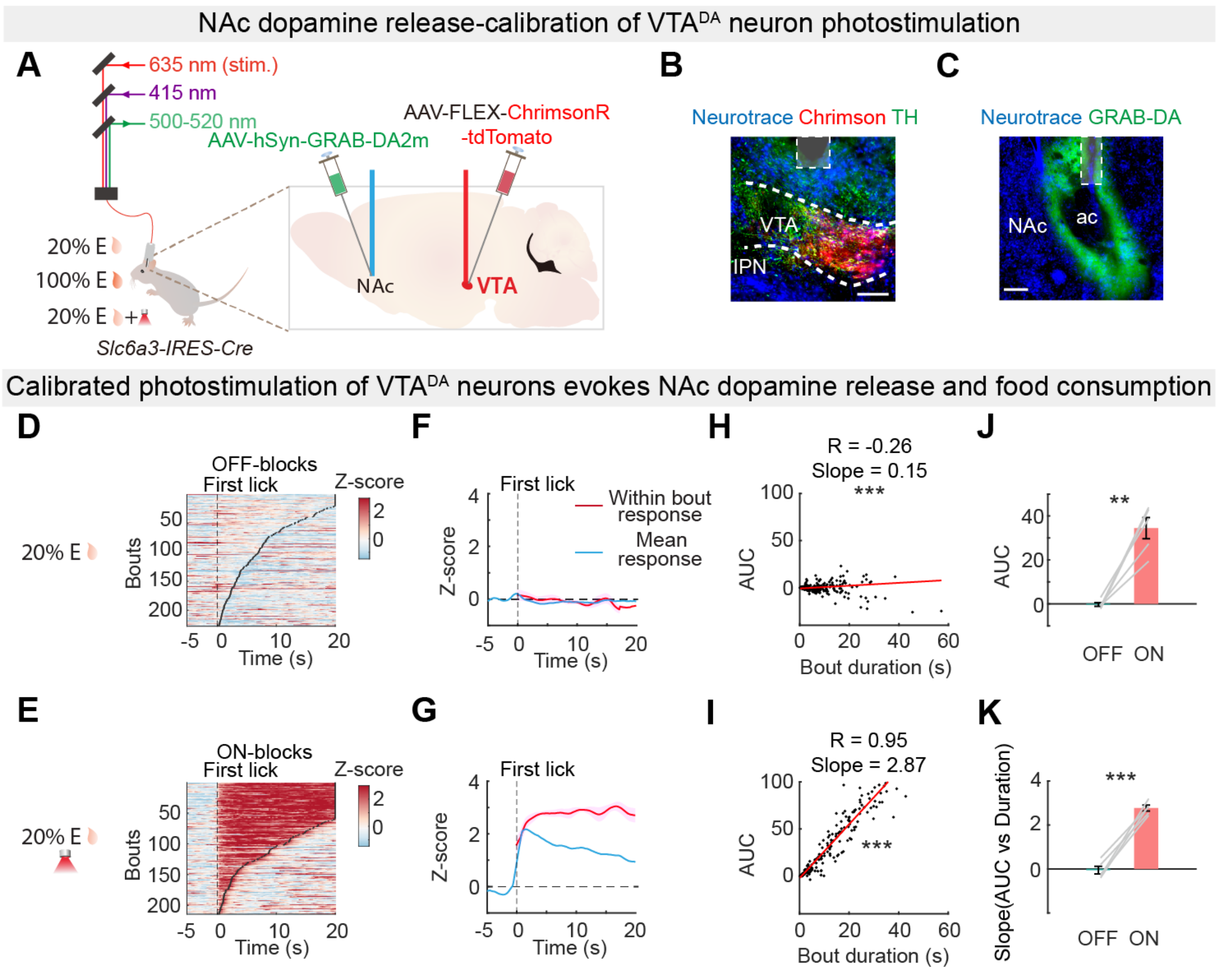


**Fig. S15. Dopamine-calibrated photostimulation of VTA$^{DA}$ neurons promotes food consumption duration and palatability**. (A) Photometry-calibrated photostimulation experimental setup (*Slc6a3-IRES-Cre* mice). (B) Expression of Chrimson (red) and TH (green) within VTA. Scale bar, 200 µm. (C) Expression of GRAB-DA2m (green) within NAc. Scale bar, 200 µm. (D-G) NAc dopamine responses during consumption of 20% Ensure with photometry-calibrated VTA$^{DA}$ neuron photostimulation in 20% Ensure in OFF-blocks (D, F) and ON-blocks (E, G). (F, G) NAc GRAB-DA2m mean responses (blue) and variable-length time mean response across all bouts (magenta) during consumption of 20% Ensure (F) and 20% Ensure with photometry-calibrated VTA$^{DA}$ neuron photostimulation (G). (H-I) Regression of NAc dopamine AUC with bout duration during consumption of 20% Ensure (H) and 20% Ensure with photometry-calibrated VTA$^{DA}$ neuron photostimulation (I) (n = 5 mice). (J-K) NAc dopamine dynamics show a larger area under the curve (AUC) (J) and a steeper slope of NAc dopamine AUC/Bout Duration (K) for photometry-calibrated VTA$^{DA}$ neuron photostimulation (paired t-test, n = 5 mice). Data are represented as mean ± SEM. $**p<0.01$, $***p<0.001$. Statistical details are provided in Table S1.

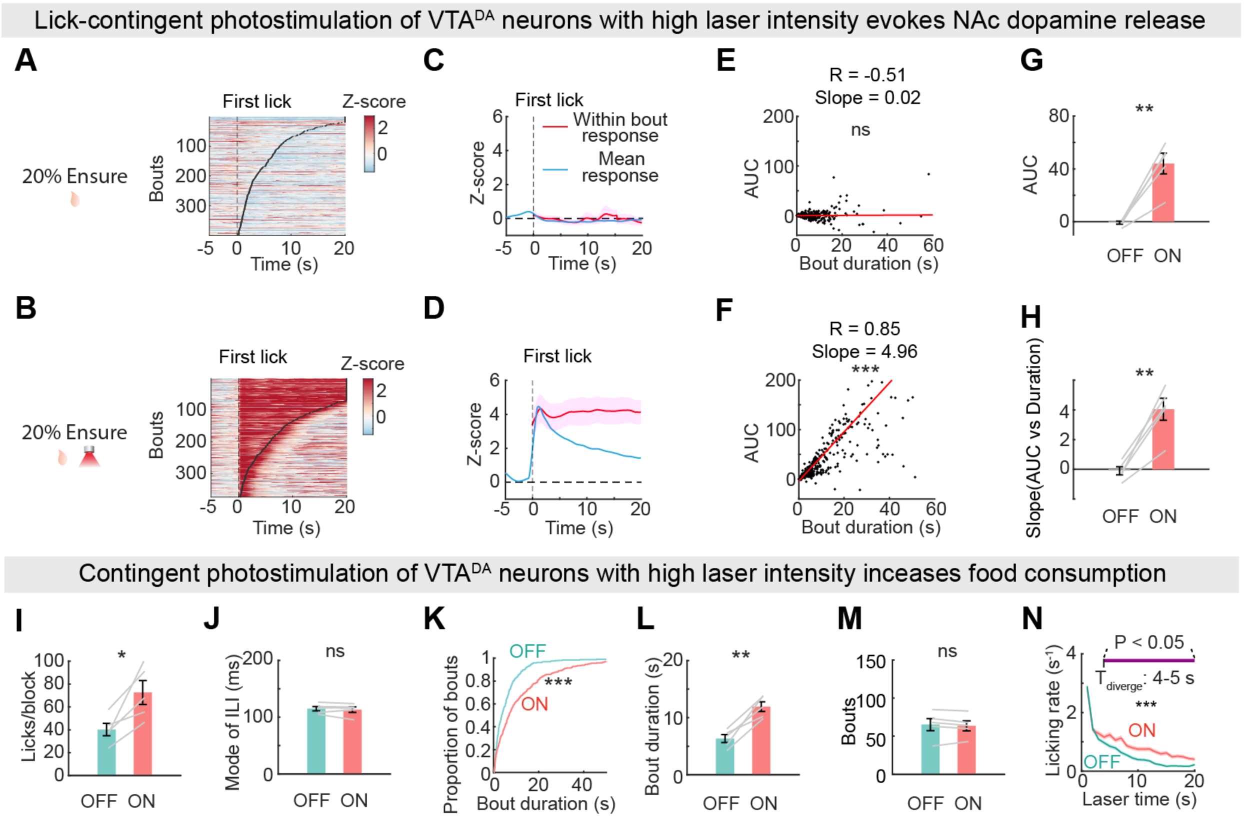


**Fig. S16. Photostimulation of VTA$^{DA}$ neuron dynamics with a higher laser intensity increases food consumption**. (A-D) NAc dopamine responses during consumption of 20% (A, C) and 20% Ensure with lick-contingent VTA$^{DA}$ neuron photostimulation with a higher laser intensity (10 mW) (B, D). (C, D) NAc dopamine mean responses (blue) and variable-length time mean response across all bouts (magenta) during consumption of 20% Ensure and 20% Ensure with contingent photostimulation with higher laser intensity. (E-F) Regression of NAc dopamine AUC with bout duration during consumption of 20% Ensure (E) and 20% Ensure with contingent photostimulation with a higher laser intensity (F) (n = 5 mice). (G-H) NAc dopamine dynamics show a larger AUC (G) and a steeper slope of GCaMP8s AUC/Bout Duration (H) for contingent photostimulation with a higher laser intensity (paired t-test, n = 5 mice). (I-N) Contingent photostimulation of VTA dopamine neurons with a higher laser intensity increases consumption (I-J), and bout duration but not bout number during laser-ON blocks (K-N) (negative binomial generalized linear mixed model, KS-test and paired t-test, n = 5 mice). Data are represented as mean ± SEM. ns $p>0.05$, *$p<0.05$, **$p<0.01$, ***$p<0.001$. Statistical details are provided in Table S1.

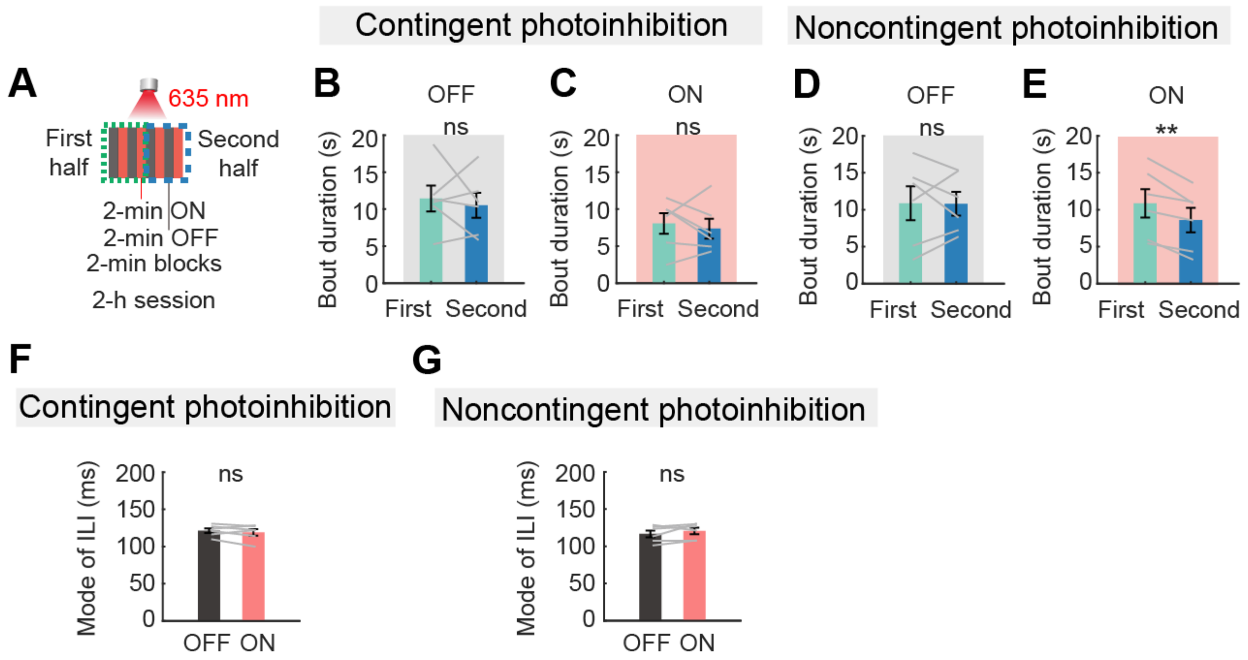


**Fig. S17. Comparison of lick-triggered photoinhibition of VTA$^{DA}$ neurons in the first and second halves of lick-contingent and noncontingent photoinhibition sessions**. (A) Schematic for comparison of bout duration from the first and second half of the session during lick-triggered photoinhibition of VTA$^{DA}$ neurons. (B-C) Bout duration was similar in the first and second halves of laser-OFF blocks (B) and laser-ON blocks (C) with lick-contingent photoinhibition of VTA$^{DA}$ neurons (paired t-test, n = 6 mice). (D-E) The bout duration in the first and second half during laser-OFF blocks was similar (D) but the bout duration was shorter in the second half of laser-ON blocks (E) of noncontingent photoinhibition of VTA$^{DA}$ neurons (paired t-test, n = 6 mice). (F-G) Contingent (F) or noncontingent (G) photoinhibition of VTA$^{DA}$ neurons does not change the fundamental lick oscillator interval (paired t-test, n = 6 mice). Data are represented as mean ± SEM. ns p>0.05, **p<0.01. Statistical details are provided in Table S1.

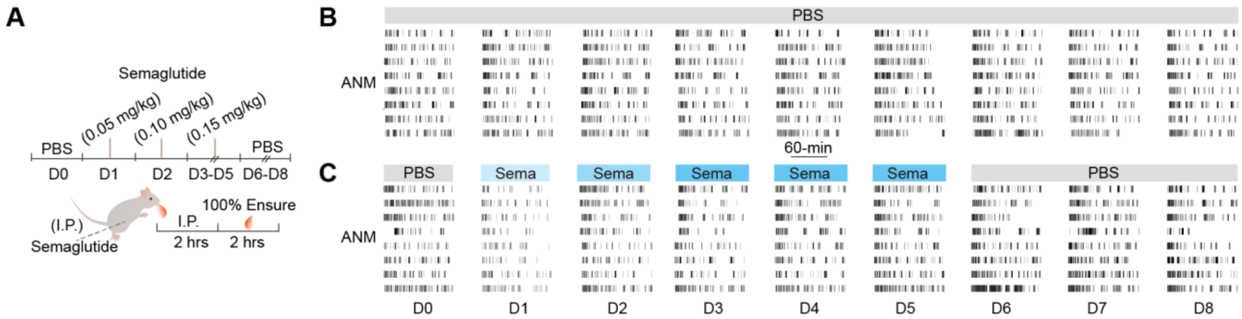


**Fig. S18. Palatable food intake during semaglutide treatment**. (A) Experimental design to test the effects of semaglutide on food consumption. (B-C) For each animal (ANM), lick raster plots of all licks for a 2-h session following injection of PBS (B) or semaglutide (C) (n = 8 mice).

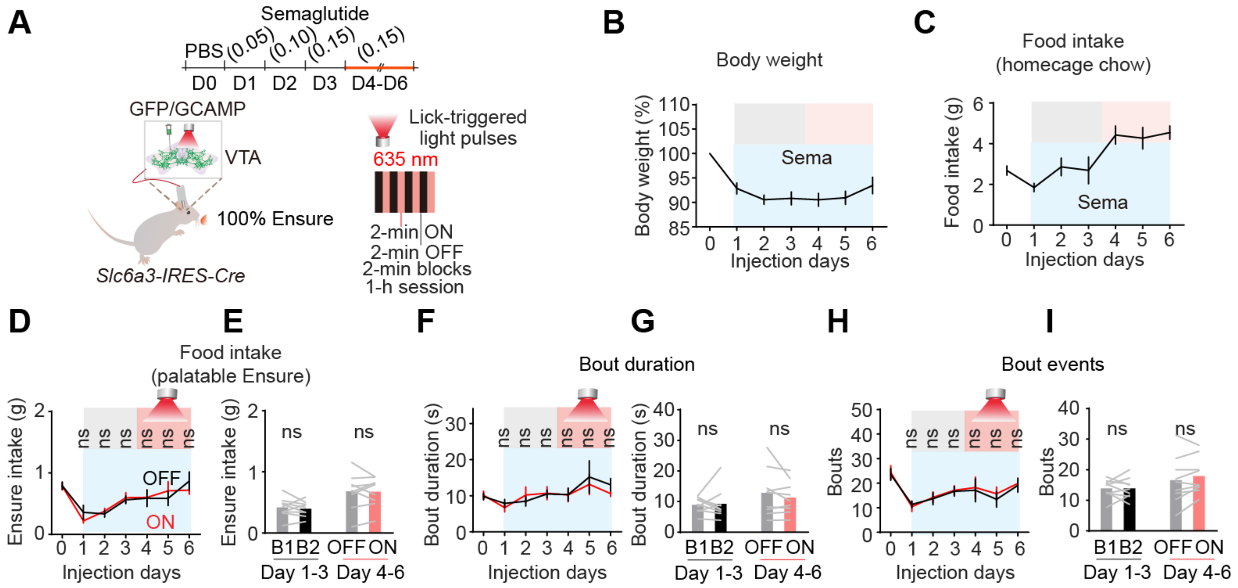


**Fig. S19. Control experiment for photoinhibition of VTA$^{DA}$ neurons during semaglutide treatment**. (A) Design of control experiment with light pulses to VTA$^{DA}$ neurons expressing GFP or GCaMP (no JAWS) during Ensure food intake from day 4 to day 6 with highest dose semaglutide treatment. (B-C) Body weight (B) and home cage chow food intake (C) during semaglutide treatment. (D-I) Similar Ensure intake (D, E), bout duration (F, G), and bout numbers (H, I) during semaglutide treatment (n = 9 mice). Days 1-3 are analyzed to show Ensure intake, bout duration, and bout number across the same alternating 2-min blocks (B1, B2) in the absence of photoinhibition. Data are represented as mean ± SEM. ns p>0.05. Statistical details are provided in Table S1.

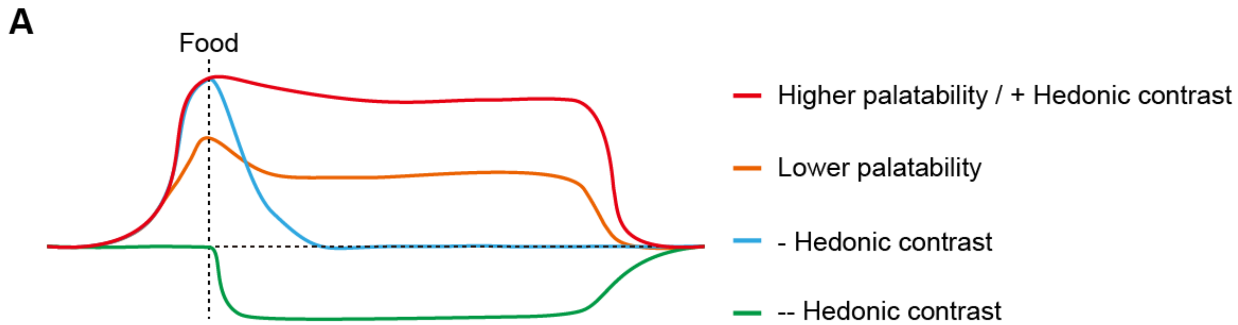


**Fig. S20. Summary of VTA$^{DA}$ neuron activity during hedonic feeding.** VTA$^{DA}$ neuron activity during consumption is scaled by palatability differences or positive and negative hedonic contrast.

**Table S1. Statistical data for all Figures.**

| Figure | Comparison | Analysis | Statistic value | N |
|---|---|---|---|---|
| 1B | Licks/block | Paired t-test | 20%E vs 100%E: $p < 0.001$ | n = 11 mice |
| 1C | Bout duration | Paired t-test | 20%E vs 100%E: $p < 0.001$ | n = 11 mice |
| 1D | Bouts | Paired t-test | 20%E vs 100%E: $p = 0.794$ | n = 11 mice |
| 1E | Licks/block | Paired t-test | 100%E + Q vs 100%E: $p < 0.001$ | n = 7 mice |
| 1F | Bout duration | Paired t-test | 100%E + Q vs 100%E: $p < 0.001$ | n = 7 mice |
| 1G | Bouts | Paired t-test | 100%E + Q vs 100%E: $p = 0.055$ | n = 7 mice |
| 1L | Licks/block | Paired t-test | BNST/LHA/VTA/PCRt (OFF vs ON): $p = 0.706$/0.910/0.012/0.377 | n = 3,6,9,4 mice |
| 1M | Proportions of bouts vs bout duration | KS test | OFF vs ON: $p = 0.410$ | n = 3 mice |
| 1M | Bouts | Paired t-test | OFF vs ON: $p = 0.836$ | n = 3 mice |
| 1N | Proportions of bouts vs bout duration | KS test | OFF vs ON: $p = 0.129$ | n = 6 mice |
| 1N | Bouts | Paired t-test | OFF vs ON: $p = 0.872$ | n = 6 mice |
| 1O | Proportions of bouts vs bout duration | KS test | OFF vs ON: $p < 0.001$ | n = 9 mice |
| 1O | Bouts | Paired t-test | OFF vs ON: $p = 0.693$ | n = 9 mice |
| 1P | Proportions of bouts vs bout duration | KS test | OFF vs ON: $p = 0.830$ | n = 4 mice |
| 1P | Bouts | Paired t-test | OFF vs ON: $p = 0.803$ | n = 4 mice |
| 1S | Licks/block | Paired t-test | OFF vs ON: $p = 0.007$ | n = 4 mice |
| 1T | Proportions of bouts vs bout duration | KS test | OFF vs ON: $p < 0.001$ | n = 4 mice |
| 1U | Bout duration | Paired t-test | OFF vs ON: $p = 0.024$ | n = 4 mice |
| 1V | Bouts | Paired t-test | OFF vs ON: $p = 0.006$ | n = 4 mice |
| 2F | AUC before vs during photostimulation | Paired t-test | PRE vs PS: $p = 0.049$ | n = 4 mice |
| 2I | AUC/Duration of laser-OFF vs laser-ON blocks | Paired t-test | OFF vs ON: $p = 0.021$ | n = 4 mice |
| 2J | Licks/block | Paired t-test | OFF vs ON: $p = 0.025$ | n = 4 mice |
| 2K | Licking rate | Negative binomial | Likelihood ratio test: Factor A (Treatment): $Chi^2(20) = 722.07$, $p < 0.001$ | n = 4 mice |

| | | | | |
|---|---|---|---|---|
| | | generalized linear mixed models | Likelihood ratio test: Factor B (Time): Chi²(38) = 296.63, $p < 0.001$ | |
| | | | Likelihood ratio test: A * B (Interaction): Chi²(19) = 96.16, $p < 0.001$ | |
| | | | Wald tests with Benjamini-Hochberg P-value adjustment:<br>0–1 s: p = 0.009<br>1–2 s: p < 0.001<br>2–3 s: p < 0.001<br>3–4 s: p < 0.001<br>4–5 s: p < 0.001<br>5–6 s: p < 0.001<br>6–7 s: p < 0.001<br>7–8 s: p < 0.001<br>8–9 s: p < 0.001<br>9–10 s: p < 0.001<br>10–11 s: p < 0.001<br>11–12 s: p < 0.001<br>12–13 s: p < 0.001<br>13–14 s: p < 0.001<br>14–15 s: p < 0.001<br>15–16 s: p < 0.001<br>16–17 s: p < 0.001<br>17–18 s: p < 0.001<br>18–19 s: p < 0.001<br>19–20 s: p < 0.001 | |
| 2L | Proportions of bouts vs bout duration | KS test | OFF vs ON: $p < 0.001$ | n = 4 mice |
| 2M | Bout duration | Paired t-test | OFF vs ON: $p = 0.005$ | n = 4 mice |
| 2N | Bouts | Paired t-test | OFF vs ON: $p = 0.391$ | n = 4 mice |
| 2S | AUC before vs during photostimulation | Paired t-test | PRE vs PS: $p = 0.018$ | n = 5 mice |
| 2V | AUC/Duration of laser-OFF vs laser-ON blocks | Paired t-test | OFF vs ON: $p = 0.036$ | n = 5 mice |
| 3F | Regression of GCaMP8s AUC with bout duration of 20% Ensure consumption | Linear regression | R = 0.483; Slope = 0.168; $p < 0.001$ | n = 13 mice |
| 3G | Regression of GCaMP8s AUC | Linear regression | R = 0.779; Slope = 0.549; $p < 0.001$ | n = 13 mice |

| | | | | |
|---|---|---|---|---|
| | with bout duration of 100% Ensure consumption | | | |
| 3H | AUC of 20% vs 100% Ensure consumption | Paired t-test | 20%E vs 100%E: *p = 0.003* | n = 13 mice |
| 3I | Slope of 20% vs 100% Ensure consumption | Paired t-test | 20%E vs 100%E: *p = 0.001* | n = 13 mice |
| 3O | Regression of GCaMP8s AUC with bout duration of 20% Ensure consumption | Linear regression | R = 0.11; Slope = 0.015; *p = 0.659* | n = 8 mice |
| 3P | Regression of GCaMP8s AUC with bout duration of 100% Ensure consumption | Linear regression | R = 0.617; Slope = 0.705; *p < 0.001* | n = 8 mice |
| 3Q | AUC of 20% vs 100% Ensure consumption | Paired t-test | 20%E vs 100%E: *p < 0.001* | n = 8 mice |
| 3R | Slope of 20% vs 100% Ensure consumption | Paired t-test | 20%E vs 100%E: *p = 0.002* | n = 8 mice |
| 3X | Regression of GRAB-DA AUC with bout duration of 20% Ensure consumption | Linear regression | R = 0.713; Slope = 0.364; *p* < 0.001 | n = 9 mice |
| 3Y | Regression of GRAB-DA AUC with bout duration of 100% Ensure consumption | Linear regression | R = 0.781; Slope = 1.169; *p* < 0.001 | n = 9 mice |
| 3Z | AUC of 20% vs 100% Ensure consumption | Paired t-test | 20%E vs 100%E: *p* = 0.015 | n = 9 mice |
| 3AA | Slope of 20% vs 100% Ensure consumption | Paired t-test | 20%E vs 100%E: *p* = 0.003 | n = 9 mice |
| 3AG | Regression of GRAB-DA AUC with bout duration of 20% Ensure consumption | Linear regression | R = 0.614; Slope = 1.215; *p* < 0.001 | n = 13 mice |

| | | | | |
|---|---|---|---|---|
| 3AH | Regression of GRAB-DA AUC with bout duration of 100% Ensure consumption | Linear regression | R = 0.838; Slope = 2.364; *p* < 0.001 | n = 13 mice |
| 3AI | AUC of 20% vs 100% Ensure consumption | Paired t-test | 20%E vs 100%E: *p* < 0.001 | n = 13 mice |
| 3AJ | Slope of 20% vs 100% Ensure consumption | Paired t-test | 20%E vs 100%E: *p* < 0.001 | n = 13 mice |
| 4J | Regression of GCaMP8s AUC with bout duration of 20% Ensure consumption with calibrated photostimulation | Linear regression | R = 0.76; Slope = 2.31; *p < 0.001* | n = 13 mice |
| 4K | Regression of GCaMP8s AUC with bout duration of 20% Ensure consumption | Linear regression | R = 0.21; Slope = -0.48; *p < 0.001* | n = 13 mice |
| 4L | AUC of OFF and ON period | Paired t-test | OFF vs ON: *p < 0.001* | n = 13 mice |
| 4M | Slope of OFF and ON period | Paired t-test | OFF vs ON: *p < 0.001* | n = 13 mice |
| 4N | Licks/block | Paired t-test | OFF vs ON: *p < 0.001* | n = 13 mice |
| 4O | Mode of ILI | Paired t-test | OFF vs ON: *p = 0.186* | n = 13 mice |
| 4P | Proportions of bouts vs bout duration | KS test | OFF vs ON: *p < 0.001* | n = 13 mice |
| 4Q | Bout duration | Paired t-test | OFF vs ON: *p < 0.001* | n = 13 mice |
| 4R | Bouts | Paired t-test | OFF vs ON: *p = 0.386* | n = 13 mice |
| 4S | Licking rate | Negative binomial generalized linear mixed models | Likelihood ratio test: Factor A (Treatment): $Chi^2(20) = 597.05$, *p* < 0.001<br>Likelihood ratio test: Factor B (Time): $Chi^2(38) = 9944.7$, *p* < 0.001<br>Likelihood ratio test: A * B (Interaction): $Chi^2(19) = 207.37$, *p* < 0.001<br>Wald tests with Benjamini-Hochberg P-value adjustment: 0–1 s: p = 0.345 | n = 13 mice |

| | | | | |
|---|---|---|---|---|
| | | | 1–2 s: p = 0.893<br>2–3 s: p = 0.659<br>3–4 s: p = 0.044<br>4–5 s: p < 0.001<br>5–6 s: p < 0.001<br>6–7 s: p < 0.001<br>7–8 s: p < 0.001<br>8–9 s: p < 0.001<br>9–10 s: p < 0.001<br>10–11 s: p < 0.001<br>11–12 s: p < 0.001<br>12–13 s: p < 0.001<br>13–14 s: p < 0.001<br>14–15 s: p < 0.001<br>15–16 s: p < 0.001<br>16–17 s: p < 0.001<br>17–18 s: p < 0.001<br>18–19 s: p < 0.001<br>19–20 s: p < 0.001 | |
| 4T | Licks/block | Paired t-test | OFF vs ON: *p = 0.986* | n = 8 mice |
| 4U | Mode of ILI | Paired t-test | OFF vs ON: *p = 0.050* | n = 8 mice |
| 4V | Proportions of bouts vs bout duration | KS test | OFF vs ON: *p = 0.77*2 | n = 8 mice |
| 4W | Bout duration | Paired t-test | OFF vs ON: *p = 0.19*4 | n = 8 mice |
| 4X | Bouts | Paired t-test | OFF vs ON: *p = 0.062* | n = 8 mice |
| 4Y | Licking rate | Negative binomial generalized linear mixed models | Likelihood ratio test: Factor A (Treatment): Chi²(20) = 26.301, *p* = 0.156 | n = 8 mice |
| | | | Likelihood ratio test: Factor B (Time): Chi²(38) = 6395.9, *p* < 0.001 | |
| | | | Likelihood ratio test: A * B (Interaction): Chi²(19) = 19.371, *p* = 0.433 | |
| | | | Wald tests with Benjamini-Hochberg P-value adjustment:<br>0–1 s: p = 0.855<br>1–2 s: p = 0.855<br>2–3 s: p = 0.971<br>3–4 s: p = 0.971<br>4–5 s: p = 0.855<br>5–6 s: p = 0.617<br>6–7 s: p = 0.971<br>7–8 s: p = 0.896 | |

| | | | | |
|---|---|---|---|---|
| | | | 8–9 s: p = 0.971<br>9–10 s: p = 0.896<br>10–11 s: p = 0.715<br>11–12 s: p = 0.324<br>12–13 s: p = 0.855<br>13–14 s: p = 0.117<br>14–15 s: p = 0.715<br>15–16 s: p = 0.896<br>16–17 s: p = 0.971<br>17–18 s: p = 0.263<br>18–19 s: p = 0.263<br>19–20 s: p = 0.263 | |
| 4AC | Bout duration | Paired t-test | OFF vs ON: *p = 0.008* | n = 5 mice |
| 4AD | Bouts | Paired t-test | OFF vs ON: *p = 0.495* | n = 5 mice |
| 5C | Licks/block | Paired t-test | OFF vs ON: *p = 0.038* | n = 6 mice |
| 5D | Bout duration | Paired t-test | OFF vs ON: *p = 0.023* | n = 6 mice |
| 5E | Bouts | Paired t-test | OFF vs ON: *p = 0.926* | n = 6 mice |
| 5F | Licking rate | Negative binomial generalized linear mixed models | Likelihood ratio test: Factor A (Treatment): Chi²(20) = 102.04, *p* < 0.001 | n = 6 mice |
| | | | Likelihood ratio test: Factor B (Time): Chi²(38) = 2187.1, *p* < 0.001 | |
| | | | Likelihood ratio test: A * B (Interaction): Chi²(19) = 46.584, *p* < 0.001 | |
| | | | Wald tests with Benjamini-Hochberg P-value adjustment:<br>0–1 s: p = 0.772<br>1–2 s: p = 0.748<br>2–3 s: p = 0.650<br>3–4 s: p = 0.858<br>4–5 s: p = 0.240<br>5–6 s: p = 0.007<br>6–7 s: p = 0.007<br>7–8 s: p = 0.050<br>8–9 s: p = 0.013<br>9–10 s: p = 0.007<br>10–11 s: p = 0.002<br>11–12 s: p = 0.002<br>12–13 s: p = 0.003<br>13–14 s: p = 0.061<br>14–15 s: p = 0.033<br>15–16 s: p = 0.015 | |

| | | | | |
|---|---|---|---|---|
| | | | 16–17 s: p = 0.007<br>17–18 s: p = 0.013<br>18–19 s: p = 0.013<br>19–20 s: p = 0.007 | |
| 5G | Licks/block | Paired t-test | OFF vs ON: $p = 0.985$ | n = 6 mice |
| 5H | Bout duration | Paired t-test | OFF vs ON: $p = 0.153$ | n = 6 mice |
| 5I | Bouts | Paired t-test | OFF vs ON: $p = 0.133$ | n = 6 mice |
| 5J | Licking rate | Negative binomial generalized linear mixed models | Likelihood ratio test: Factor A (Treatment): $Chi^2(20) = 29.036$, $p = 0.087$<br>Likelihood ratio test: Factor B (Time): $Chi^2(38) = 3196.6$, $p < 0.001$<br>Likelihood ratio test: A * B (Interaction): $Chi^2(19) = 26.825$, $p = 0.109$<br>Wald tests with Benjamini-Hochberg P-value adjustment:<br>0–1 s: p = 0.586<br>1–2 s: p = 0.646<br>2–3 s: p = 0.583<br>3–4 s: p = 0.440<br>4–5 s: p = 0.727<br>5–6 s: p = 0.440<br>6–7 s: p = 0.821<br>7–8 s: p = 0.583<br>8–9 s: p = 0.583<br>9–10 s: p = 0.583<br>10–11 s: p = 0.433<br>11–12 s: p = 0.433<br>12–13 s: p = 0.287<br>13–14 s: p = 0.433<br>14–15 s: p = 0.732<br>15–16 s: p = 0.433<br>16–17 s: p = 0.568<br>17–18 s: p = 0.599<br>18–19 s: p = 0.821<br>19–20 s: p = 0.238 | n = 6 mice |
| 6B | Normalized body weight during days 1-5 with PBS treatment versus semaglutide treatment | Repeated measures ANOVA with the Geisser-Greenhouse correction; | Factor A (Treatment): $F(1, 14) = 85.52$, $p < 0.001$;<br>Factor B (Time): $F(1.986, 27.80) = 7.881$, $p = 0.002$;<br>A * B (Interaction): $F(4, 56) = 5.607$, $p < 0.001$; | n = 8 mice |

| | | | | |
|---|---|---|---|---|
| | | Holm-Šídák's multiple comparisons test | Post hoc Holm-Šídák's multiple comparisons test<br>Day 1: t = 5.289, *p < 0.001*;<br>Day 2: t = 7.153, *p < 0.001*;<br>Day 3: t = 8.674, *p < 0.001*;<br>Day 4: t = 8.491, *p < 0.001*;<br>Day 5: t = 8.616, *p < 0.001*; | |
| 6C | Homecage chow intake during days 1-5 with PBS treatment versus semaglutide treatment | Repeated measures ANOVA with the Geisser-Greenhouse correction; Holm-Šídák's multiple comparisons test | Factor A (Treatment): F (1, 14) = 30.50, *p < 0.001*;<br>Factor B (Time): F (2.098, 29.37) = 2.285, *p = 0.117;*<br>A * B (Interaction): F (4, 56) = 3.983, *p = 0.007*;<br>Post hoc Holm-Šídák's multiple comparisons test<br>Day 1: t = 8.895, *p < 0.001*;<br>Day 2: t = 6.863, *p < 0.001*;<br>Day 3: t = 1.396, *p = 0.253*;<br>Day 4: t = 5.137, *p = 0.001*;<br>Day 5: t = 1.591, *p = 0.253*; | n = 8 mice |
| 6D | Ensure intake during days 1-5 with PBS treatment versus semaglutide treatment | Repeated measures ANOVA with the Geisser-Greenhouse correction; Holm-Šídák's multiple comparisons test | Factor A (Treatment): F (1, 14) = 21.69, *p < 0.001*;<br>Factor B (Time): F (2.665, 37.31) = 18.01, *p < 0.001*;<br>A * B (Interaction): F (4, 56) = 14.93, *p < 0.001*;<br>Post hoc Holm-Šídák's multiple comparisons test<br>Day 1: t = 8.677, *p < 0.001*;<br>Day 2: t = 7.545, *p < 0.001*;<br>Day 3: t = 2.685, *p = 0.049*;<br>Day 4: t = 2.764, *p = 0.049*;<br>Day 5: t = 0.737, *p = 0.475*; | n = 8 mice |
| 6E | Bout duration during days 1-5 with PBS treatment versus semaglutide treatment | Two-way ANOVA; Holm-Šídák's multiple comparisons test | Factor A (Treatment): F (1, 70) = 7.783, *p = 0.007*;<br>Factor B (Time): F (2.883, 40.36) = 2.523, *p = 0.073*;<br>A * B (Interaction): F (4, 70) = 0.366, *p = 0.832*;<br>Post hoc Holm-Šídák's multiple comparisons test<br>Day 1: t = 1.881, *p = 0.282*<br>Day 2: t = 1.881, *p = 0.282*;<br>Day 3: t = 0.913, *p = 0.672*;<br>Day 4: t = 1.021, *p = 0.672*;<br>Day 5: t = 0.541, *p = 0.672*; | n = 8 mice |

| | | | | |
|---|---|---|---|---|
| 6F | Bout numbers during days 1-5 with PBS treatment versus semaglutide treatment | Repeated measures ANOVA with the Geisser-Greenhouse correction; Holm-Šídák's multiple comparisons test | Factor A (Treatment): $F (1, 14) = 0.121$, *$p = 0.734$*<br>Factor B (Time): $F (3.165, 44.31) = 5.514$, *$p = 0.002$*;<br>A * B (Interaction): $F (4, 56) = 3.086$, *$p = 0.023$*;<br>Post hoc Holm-Šídák's multiple comparisons test<br>Day 1: $t = 3.267$, *$p = 0.035$*;<br>Day 2: $t = 0.294$, *$p = 0.987$*;<br>Day 3: $t = 0.251$, *$p = 0.987$*;<br>Day 4: $t = 0.452$, *$p = 0.987$*;<br>Day 5: $t = 0.308$, *$p = 0.987$*; | n = 8 mice |
| 6H | Normalized body weight during days 1-5 with PBS treatment versus semaglutide treatment | Repeated measures ANOVA with the Geisser-Greenhouse correction; Holm-Šídák's multiple comparisons test | Factor A (Treatment): $F (1, 14) = 57.55$, *$p < 0.001$*;<br>Factor B (Time): $F (3.034, 42.48) = 0.867$, *$p = 0.467$*;<br>A * B (Interaction): $F (4, 56) = 2.163$, *$p = 0.085$*;<br>Post hoc Holm-Šídák's multiple comparisons test<br>Day 1: $t = 4.800$, *$p < 0.001$*;<br>Day 2: $t = 6.316$, *$p < 0.001$*;<br>Day 3: $t = 6.855$, *$p < 0.001$*;<br>Day 4: $t = 5.192$, *$p < 0.001$*;<br>Day 5: $t = 6.362$, *$p < 0.001$*; | n = 8 mice |
| 6I | Homecage chow intake during days 1-5 with PBS treatment versus semaglutide treatment | Repeated measures ANOVA with the Geisser-Greenhouse correction; Holm-Šídák's multiple comparisons test | Factor A (Treatment): $F (1, 14) = 6.077$, *$p = 0.027$*;<br>Factor B (Time): $F (2.429, 34.01) = 3.702$, *$p = 0.028$*;<br>A * B (Interaction): $F (4, 56) = 7.763$, *$p < 0.001$*;<br>Post hoc Holm-Šídák's multiple comparisons test<br>Day 1: $t = 6.368$, *$p < 0.001$*;<br>Day 2: $t = 2.474$, *$p = 0.104$*;<br>Day 3: $t = 2.569$, *$p = 0.104$*;<br>Day 4: $t = 0.145$, *$p = 0.887$*;<br>Day 5: $t = 2.119$, *$p = 0.105$*; | n = 8 mice |
| 6J | Ensure intake during days 1-5 with PBS treatment versus semaglutide treatment | Repeated measures ANOVA with the Geisser-Greenhouse correction; | Factor A (Treatment): $F (1, 14) = 9.816$, *$p = 0.007$*;<br>Factor B (Time): $F (2.927, 40.97) = 6.726$, *$p < 0.001$*;<br>A * B (Interaction): $F (4, 56) = 8.298$, *$p < 0.001$*; | n = 8 mice |

| | | | | |
|---|---|---|---|---|
| | | Holm-Šídák's multiple comparisons test | Post hoc Holm-Šídák's multiple comparisons test<br>Day 1: t = 6.904, $p < 0.001$;<br>Day 2: t = 4.670, $p = 0.004$;<br>Day 3: t = 1.035, $p = 0.686$;<br>Day 4: t = 0.887, $p = 0.686$;<br>Day 5: t = 0.028, $p = 0.978$; | |
| 6K | Bout duration during days 1-5 with PBS treatment versus semaglutide treatment | Repeated measures ANOVA with the Geisser-Greenhouse correction; Holm-Šídák's multiple comparisons test | Factor A (Treatment): F (1, 14) = 1.294, $p = 0.274$;<br>Factor B (Time): F (3.063, 42.88) = 1.250, $p = 0.304$;<br>A * B (Interaction): F (4, 56) = 7.053, $p<0.001$;<br>Post hoc Holm-Šídák's multiple comparisons test<br>Day 1: t = 3.922, $p = 0.021$;<br>Day 2: t = 3.417, $p = 0.027$;<br>Day 3: t = 0.078, $p = 0.940$<br>Day 4: t = 1.614, $p = 0.339$;<br>Day 5: t = 1.494, $p = 0.339$; | n = 8 mice |
| 6L | Bout numbers during days 1-5 with PBS treatment versus semaglutide treatment | Repeated measures ANOVA with the Geisser-Greenhouse correction; Holm-Šídák's multiple comparisons test | Factor A (Treatment): F (1, 14) = 4.039, $p = 0.064$;<br>Factor B (Time): F (3.530, 49.42) = 6.384, $p < 0.001$;<br>A * B (Interaction): F (4, 56) = 0.803, $p = 0.529$;<br>Post hoc Holm-Šídák's multiple comparisons test<br>Day 1: t = 3.223, $p = 0.052$;<br>Day 2: t = 0.889, $p = 0.549$;<br>Day 3: t = 1.023, $p = 0.549$;<br>Day 4: t = 1.649, $p = 0.405$;<br>Day 5: t = 1.337, $p = 0.497$; | n = 8 mice |
| 6N | AUC during days 0-5 with PBS treatment versus semaglutide treatment | Repeated measures ANOVA with the Geisser-Greenhouse correction; Holm-Šídák's multiple comparisons test | Factor A (Treatment): F (1, 14) = 0.050, $p = 0.826$;<br>Factor B (Time): F (3.207, 44.89) = 0.359, $p = 0.796$;<br>A * B (Interaction): F (5, 70) = 2.430, $p = 0.043$;<br>Post hoc Holm-Šídák's multiple comparisons test<br>Day 0: t = 0.150, $P =0.883$;<br>Day 1: t = 2.441, $p = 0.033$;<br>Day 2: t = 1.215, $p = 0.244$;<br>Day 3: t = 0.378, $p = 0.715$;<br>Day 4: t = 1.101, $p = 0.291$; | n = 8 mice |

| | | | | |
|---|---|---|---|---|
| | | | Day 5: t = 1.865, *p = 0.091*; | |
| 7C | Proportions of lick vs licking rate | KS test | OFF vs ON: *p = 0.003* | n = 10 mice |
| 7D | Bout duration | Paired t-test | OFF vs ON: *p = 0.020* | n = 10 mice |
| 7E | Bouts | Paired t-test | OFF vs ON: *p = 0.654* | n = 10 mice |
| 7I | Daily Ensure intake of day 1-3 semaglutide treatment during Block 1 compared to Block 2 | Repeated measures ANOVA with the Geisser-Greenhouse correction; Holm-Šídák's multiple comparisons test | Factor A (Treatment): F (1.000, 9.000) = 0.9492, *p = 0.355*; | n = 10 mice |
| | | | Factor B (Time): F (1.702, 15.32) = 45.53, *p < 0.001*; | |
| | | | A * B (Interaction): F (1.287, 11.58) = 0.7985, *p = 0.421;* | |
| | | | Post hoc Holm-Šídák's multiple comparisons test<br>Day 1: t = 0.580, *p = 0.741*;<br>Day 2: t = 1.333, *p = 0.517*;<br>Day 3: t = 0.718, *p = 0.741*; | |
| 7I | Daily Ensure intake of day 4-6 semaglutide treatment during the laser-OFF blocks compared to laser-ON blocks | Repeated measures ANOVA with the Geisser-Greenhouse correction; Holm-Šídák's multiple comparisons test | Factor A (Treatment): F (1.000, 9.000) = 18.89, *p = 0.002*; | n = 10 mice |
| | | | Factor B (Time): F (1.747, 15.72) = 3.100, *p = 0.079*; | |
| | | | A * B (Interaction): F (1.989, 17.90) = 2.570, *p = 0.105;* | |
| | | | Post hoc Holm-Šídák's multiple comparisons test<br>Day 4: t = 3.263, *p = 0.024*;<br>Day 5: t = 1.039, *p = 0.326*;<br>Day 6: t = 3.381, *p = 0.024*; | |
| 7J | Averaged Ensure intake of day 1-3 or day 4-6 semaglutide treatment during different blocks | Paired t-test | B1 vs B2 (day 1-3): *p = 0.355*;<br>OFF vs ON (day 4-6): *p = 0.002* | n = 10 mice |
| 7K | Daily bout duration of day 1-3 semaglutide treatment during Block 1 compared to Block 2 | Repeated measures ANOVA with the Geisser-Greenhouse correction; Holm-Šídák's multiple comparisons test | Factor A (Treatment): F (1.000, 9.000) = 0.029, *p = 0.869*; | n = 10 mice |
| | | | Factor B (Time): F (1.715, 15.44) = 8.059, *p = 0.005*; | |
| | | | A * B (Interaction): F (1.719, 15.47) = 0.297, *p = 0.715*; | |
| | | | Post hoc Holm-Šídák's multiple comparisons test<br>Day 1: t = 0.486, *p = 0.925*;<br>Day 2: t = 0.576, *p = 0.925*;<br>Day 3: t = 0.351, *p = 0.925*; | |

| | | | | |
|---|---|---|---|---|
| 7K | Daily bout duration of day 4-6 semaglutide treatment during the laser-OFF blocks compared to laser-ON blocks | Repeated measures ANOVA with the Geisser-Greenhouse correction; Holm-Šídák's multiple comparisons test | Factor A (Treatment): F (1.000, 9.000) = 18.31, *p = 0.002*;<br>Factor B (Time): F (1.644, 14.80) = 3.370, *p = 0.070*;<br>A * B (Interaction): F (1.311, 11.80) = 1.881, *p = 0.198*;<br>Post hoc Holm-Šídák's multiple comparisons test<br>Day 4: t = 3.794, *p = 0.013*;<br>Day 5: t = 3.632, *p = 0.013*;<br>Day 6: t = 2.775, *p = 0.021*; | n = 10 mice |
| 7L | Averaged bout duration of day 1-3 or day 4-6 semaglutide treatment during different blocks | Paired t-test | B1 vs B2 (day 1-3): *p = 0.869;*<br>OFF vs ON (day 4-6): *p = 0.002* | n = 10 mice |
| 7M | Daily bout numbers of day 1-3 semaglutide treatment during Block 1 compared to Block 2 | Repeated measures ANOVA with the Geisser-Greenhouse correction; Holm-Šídák's multiple comparisons test | Factor A (Treatment): F (1.000, 9.000) = 0.787, *p = 0.398*;<br>Factor B (Time): F (1.415, 12.73) = 2.714, *p = 0.116*;<br>A * B (Interaction): F (1.781, 16.03) = 1.858, *p = 0.190*;<br>Post hoc Holm-Šídák's multiple comparisons test<br>Day 1: t = 1.076, *p = 0.524*;<br>Day 2: t = 1.964, *p = 0.224*;<br>Day 3: t = 0.179, *p = 0.862*; | n = 10 mice |
| 7M | Daily bout numbers of day 4-6 semaglutide treatment during the laser-OFF blocks compared to laser-ON blocks | Repeated measures ANOVA with the Geisser-Greenhouse correction; Holm-Šídák's multiple comparisons test | Factor A (Treatment): F (1.000, 9.000) = 4.520, *p = 0.062*;<br>Factor B (Time): F (1.334, 12.01) = 1.451, *p = 0.263*;<br>A * B (Interaction): F (1.898, 17.09) = 0.655, *p = 0.524*;<br>Post hoc Holm-Šídák's multiple comparisons test<br>Day 4: t = 2.187, *p = 0.160*;<br>Day 5: t = 0.825, *p = 0.676*;<br>Day 6: t = 0.220, *p = 0.831*; | n = 10 mice |
| 7N | Averaged bout numbers of day 1-3 or day 4-6 semaglutide treatment during different blocks | Paired t-test | B1 vs B2 (day 1-3): *p = 0.398;*<br>OFF vs ON (day 4-6): *p = 0.969* | n = 10 mice |

| | | | | |
|---|---|---|---|---|
| S2G | Proportions of bouts vs bout duration | KS test | 20%E vs 100%E: $p < 0.001$ | n = 11 mice |
| S2H | Licking rate | Negative binomial generalized linear mixed models | Likelihood ratio test: Factor A (Treatment): $Chi^2(20) = 1919.9$, $p < 0.001$ | n = 11 mice |
| | | | Likelihood ratio test: Factor B (Time): $Chi^2(38) = 4018.7$, $p < 0.001$ | |
| | | | Likelihood ratio test: A * B (Interaction): $Chi^2(19) = 520.89$, $p < 0.001$ | |
| | | | Wald tests with Benjamini-Hochberg P-value adjustment:<br>0–1 s: p = 0.101<br>1–2 s: p < 0.001<br>2–3 s: p < 0.001<br>3–4 s: p < 0.001<br>4–5 s: p < 0.001<br>5–6 s: p < 0.001<br>6–7 s: p < 0.001<br>7–8 s: p < 0.001<br>8–9 s: p < 0.001<br>9–10 s: p < 0.001<br>10–11 s: p < 0.001<br>11–12 s: p < 0.001<br>12–13 s: p < 0.001<br>13–14 s: p < 0.001<br>14–15 s: p < 0.001<br>15–16 s: p < 0.001<br>16–17 s: p < 0.001<br>17–18 s: p < 0.001<br>18–19 s: p < 0.001<br>19–20 s: p < 0.001 | |
| S2J | Proportions of bouts vs bout duration | KS test | 100%E+Q vs 100%E: $p < 0.001$ | n = 7 mice |
| S2K | Licking rate | Negative binomial generalized linear mixed models | Likelihood ratio test: Factor A (Treatment): $Chi^2(20) = 1042.3$, $p < 0.001$ | n = 7 mice |
| | | | Likelihood ratio test: Factor B (Time): $Chi^2(38) = 1305.5$, $p < 0.001$ | |
| | | | Likelihood ratio test: A * B (Interaction): $Chi^2(19) = 350.15$, $p < 0.001$ | |
| | | | Wald tests with Benjamini-Hochberg P-value adjustment: | |

| | | | | |
|---|---|---|---|---|
| | | | 0–1 s: p = 0.172<br>1–2 s: p = 0.051<br>2–3 s: p < 0.001<br>3–4 s: p < 0.001<br>4–5 s: p < 0.001<br>5–6 s: p < 0.001<br>6–7 s: p < 0.001<br>7–8 s: p < 0.001<br>8–9 s: p < 0.001<br>9–10 s: p < 0.001<br>10–11 s: p < 0.001<br>11–12 s: p < 0.001<br>12–13 s: p < 0.001<br>13–14 s: p < 0.001<br>14–15 s: p < 0.001<br>15–16 s: p < 0.001<br>16–17 s: p < 0.001<br>17–18 s: p < 0.001<br>18–19 s: p < 0.001<br>19–20 s: p < 0.001 | |
| S3D | Place preference | Repeated measures ANOVA; Holm-Šídák's multiple comparisons test | Factor A (Photostimulation): F (2, 6) = 7.032, *p* = 0.027 | n = 4 mice |
| | | | Pre-test vs Cond1: t = 3.508, *p = 0.025*;<br>Pre-test vs Cond2: t = 2.902, *p = 0.027* | |
| S3E | Licks/block | Paired t-test | OFF vs ON: *p = 0.904* | n = 5 mice |
| S3F | Proportions of bouts vs bout duration | KS test | OFF vs ON: *p = 0.893* | n = 5 mice |
| S3G | Bout duration | Paired t-test | OFF vs ON: *p = 0.904* | n = 5 mice |
| S3H | Bouts | Paired t-test | OFF vs ON: *p = 0.143* | n = 5 mice |
| S4E | Proportions of cells | Unpaired t-test | VGAT/GFP vs TH/GFP: *p < 0.001* | n = 22 VTA sections from 2 mice |
| S6J | AUC of before vs during food consumption | Paired t-test | Pre-100E vs 100E: *p = 0.128* | n = 4 mice |
| S7F | Regression of GCaMP8m AUC with bout duration of 20% Ensure consumption | Linear regression | R = 0.531; Slope = 0.065; *p = 0.022* | n = 4 mice |
| S7G | Regression of GCaMP8m AUC with bout duration of | Linear regression | R = 0.812; Slope = 0.948; *p < 0.001* | n = 4 mice |

| | | | | |
|---|---|---|---|---|
| | 100% Ensure consumption | | | |
| S7K | Regression of GFP AUC with bout duration of 20% Ensure consumption | Linear regression | R = -0.434; Slope = -0.037; *p = 0.131* | n = 3 mice |
| S8A | Regression of bout duration of 20% Ensure consumption with bout indices across feeding session | Linear regression | R = 0.02; Slope = -0.001; *p = 0.904* | n = 13 mice |
| S8B | Regression of GCaMP8m AUC with bout indices across feeding session | Linear regression | R = -0.031; Slope = -0.013; *p = 0.003* | n = 13 mice |
| S8C | Regression of bout duration of 100% Ensure consumption with bout indices across feeding session | Linear regression | R = 0.014; Slope = -0.001; *p = 0.987* | n = 13 mice |
| S8D | Regression of GCaMP8m AUC with bout indices across feeding session | Linear regression | R = -0.167; Slope = -0.082; *p < 0.001* | n = 13 mice |
| S8E | Calories of 20% vs 100% Ensure consumption | Paired t-test | *p < 0.001* | n = 13 mice |
| S9F | Regression of GCaMP8m AUC with bout duration of 100% Ensure consumption before PBS injection | Linear regression | R = 0.754; Slope = 0.659; *p < 0.001* | n = 7 mice |
| S9G | Regression of GCaMP8m AUC with bout duration of 100% Ensure consumption after PBS injection | Linear regression | R = 0.537; Slope = 0.321; *p < 0.001* | n = 7 mice |
| S9M | Regression of GCaMP8m AUC with bout duration of | Linear regression | R = 0.588; Slope = 0.245; *p < 0.001* | n = 7 mice |

| | | | | |
|---|---|---|---|---|
| | 100% Ensure consumption before LiCl injection | | | |
| S9N | Regression of GCaMP8m AUC with bout duration of 100% Ensure consumption after LiCl injection | Linear regression | R = 0.328; Slope = -0.092; *p = 0.038* | n = 7 mice |
| S9O | AUC of 100% Ensure consumption before and after PBS treatment versus before and after LiCl treatment | 1-Factor (Time) Repeated measures ANOVA | Factor A (Treatment): F (1, 12) = 0.295, *p = 0.597*;<br>Factor B (Time): F (1, 12) = 12.66, *p = 0.004*;<br>A * B (Interaction): F (1, 12) = 4.822, *p = 0.049*; | n = 7 mice |
| S10F | Regression of GCaMP8m AUC with bout duration of 20% Ensure consumption for FED mice | Linear regression | R = 0.489; Slope = 0.231; $p < 0.001$ | n = 7 mice |
| S10G | Regression of GCaMP8m AUC with bout duration of 20% Ensure consumption for RES mice | Linear regression | R = 0.573; Slope = 0.367; $p < 0.001$ | n = 7 mice |
| S10H | AUC of 20% Ensure consumption FED vs RES | Paired t-test | FED *vs* RES: *p = 0.035* | n = 7 mice |
| S10I | Slope of 20% Ensure consumption FED vs RES | Paired t-test | FED *vs* RES: *p = 0.006* | n = 7 mice |
| S11A | Bout duration of 20% vs 100% Ensure consumption in separate sessions | Paired t-test | *p = 0.335* | n = 13 mice |
| S11A | Bout duration of 20% vs 100% Ensure consumption within a session | Paired t-test | $p < 0.001$ | n = 8 mice |
| S11B | Pearson correlation coefficient of 20% vs 100% Ensure consumption in separate sessions | Paired t-test | *p = 0.153* | n = 13 mice |

| | | | | |
|---|---|---|---|---|
| S11B | Pearson correlation coefficient of 20% vs 100% Ensure consumption within a session | Paired t-test | *$p < 0.001$* | n = 8 mice |
| S12F | Regression of GCaMP8m AUC with bout duration of 100% Ensure with quinine consumption | Linear regression | R = 0.180; Slope = 0.297; *$p < 0.001$* | n = 7 mice |
| S12G | Regression of GCaMP8m AUC with bout duration of 100% Ensure consumption | Linear regression | R = 0.704; Slope = 2.390; *$p < 0.001$* | n = 7 mice |
| S12H | AUC of 100% Ensure with quinine vs 100% Ensure consumption | Paired t-test | 100% Ensure with quinine vs 100% Ensure: *$p = 0.002$* | n = 7 mice |
| S12I | Slope of 100% Ensure with quinine vs 100% Ensure consumption | Paired t-test | 100% Ensure with quinine vs 100% Ensure: *$p = 0.021$* | n = 7 mice |
| S13A | Licks/block | Paired t-test | OFF vs ON: *$p = 0.433$* | n = 6 mice |
| S13B | Proportions of bouts vs bout duration | KS test | OFF vs ON: *$p = 0.912$* | n = 6 mice |
| S13C | Bout duration | Paired t-test | OFF vs ON: *$p = 0.863$* | n = 6 mice |
| S13D | Bouts | Paired t-test | OFF vs ON: *$p = 0.357$* | n = 6 mice |
| S13E | Regression of bout duration of 20% Ensure consumption with bout indices across feeding session | Linear regression | R = 0.011; Slope = 0.006; *$p = 0.188$* | n = 13 mice |
| S13F | Regression of GCaMP8s AUC with bout indices across feeding session | Linear regression | R = -0.08; Slope = -0.02; *$p < 0.001$* | n = 13 mice |
| S13G | Regression of bout duration of 20% Ensure consumption and calibrated photostimulation with bout indices | Linear regression | R = -0.01; Slope = 0.003; *$p = 0.680$* | n = 13 mice |

| | | | | |
|---|---|---|---|---|
| | across feeding session | | | |
| S13H | Regression of GCaMP8s AUC with bout indices across feeding session | Linear regression | R = -0.03; Slope = -0.04; *p = 0.029* | n = 13 mice |
| S13I | Calories of OFF and ON periods | Paired t-test | *p = 0.002* | n = 13 mice |
| S14E | Regression of GCaMP8s AUC with bout duration of 20% Ensure consumption | Linear regression | R = 0.002; Slope = -0.501; *p < 0.001* | n = 8 mice |
| S14F | Regression of GCaMP8s AUC with bout duration of 20% Ensure consumption with contingent photostimulation of high laser intensity | Linear regression | R = 0.833; Slope = 3.029; *p < 0.001* | n = 8 mice |
| S14G | AUC of OFF and ON period | Paired t-test | OFF vs ON: *p < 0.001* | n = 8 mice |
| S14H | Slope of OFF and ON period | Paired t-test | OFF vs ON: *p < 0.001* | n = 8 mice |
| S14I | Licks/block | Paired t-test | OFF vs ON: *p < 0.001* | n = 8 mice |
| S14J | ILI | Paired t-test | OFF vs ON: *p = 0.478* | n = 8 mice |
| S14K | Proportions of bouts vs bout duration | KS test | OFF vs ON: *p < 0.001* | n = 8 mice |
| S14L | Bout duration | Paired t-test | OFF vs ON: *p < 0.001* | n = 8 mice |
| S14M | Bouts | Paired t-test | OFF vs ON: *p = 0.782* | n = 8 mice |
| S14N | Licking rate | Negative binomial generalized linear mixed models | Likelihood ratio test: Factor A (Treatment): $Chi^2(20) = 491.1$, *p* < 0.001 | n = 8 mice |
| | | | Likelihood ratio test: Factor B (Time): $Chi^2(38) = 7933.2$, *p* < 0.001 | |
| | | | Likelihood ratio test: A * B (Interaction): $Chi^2(19) = 219.05$, *p* < 0.001 | |
| | | | Wald tests with Benjamini-Hochberg P-value adjustment: 0–1 s: p = 0.835 | |

| | | | | |
|---|---|---|---|---|
| | | | 1–2 s: p = 0.340<br>2–3 s: p = 0.688<br>3–4 s: p = 0.112<br>4–5 s: p = 0.005<br>5–6 s: p < 0.001<br>6–7 s: p < 0.001<br>7–8 s: p < 0.001<br>8–9 s: p < 0.001<br>9–10 s: p < 0.001<br>10–11 s: p < 0.001<br>11–12 s: p < 0.001<br>12–13 s: p < 0.001<br>13–14 s: p < 0.001<br>14–15 s: p < 0.001<br>15–16 s: p < 0.001<br>16–17 s: p < 0.001<br>17–18 s: p < 0.001<br>18–19 s: p < 0.001<br>19–20 s: p < 0.001 | |
| S14O | Regression of bout duration of 20% Ensure consumption with bout indices across each feeding session | Linear regression | R = -0.14; Slope = -0.008; *p = 0.065* | n = 8 mice |
| S14P | Regression of GCaMP8s AUC with bout indices across each feeding session | Linear regression | R = -0.07; Slope = -0.001; *p = 0.701* | n = 8 mice |
| S14Q | Regression of bout duration of 20% Ensure consumption and calibrated photostimulation with bout indices across each feeding session | Linear regression | R = -0.08; Slope = 0.01; *p = 0.180* | n = 8 mice |
| S14R | Regression of GCaMP8s AUC with bout indices across each feeding session | Linear regression | R = -0.01; Slope = 0.04; *p = 0.240* | n = 8 mice |
| S14S | Calories of Laser-OFF and Laser-ON periods | Paired t-test | *p < 0.001* | n = 8 mice |

| | | | | |
|---|---|---|---|---|
| S14T | Regression of lick frequency with blocks across feeding sessions (20% Ensure) | Linear regression | R = -0.25; Slope = - 0.01; *p = 0.005* | n = 1 mouse |
| S14U | Regression of lick frequency with blocks across of feeding session (20% Ensure and high laser photostimulation) | Linear regression | R = -0.03; Slope = 0.002; *p = 0.763* | n = 1 mouse |
| S14V | Pearson correlation coefficient of 20% Ensure consumption vs 20% Ensure consumption and high laser photostimulation | Paired t-test | *p = 0.003* | n = 8 mice |
| S14W | Regression of lick frequency with blocks across feeding sessions (20% Ensure) | Linear regression | R = 0.14; Slope = 0.01; *p = 0.020* | n = 1 mouse |
| S14X | Regression of lick frequency with blocks across of feeding session (20% Ensure and calibrated photostimulation) | Linear regression | R = -0.01; Slope = 0.001; *p = 0.853* | n = 1 mouse |
| S14Y | Pearson correlation coefficient of 20% Ensure consumption vs 20% Ensure consumption and calibrated photostimulation | Paired t-test | *p = 0.776* | n = 13 mice |
| S15H | Regression of GRAB-DA2m AUC with bout duration of 20% Ensure consumption | Linear regression | R = -0.262; Slope = 0.149; *p < 0.001* | n = 5 mice |
| S15I | Regression of GRAB-DA2m AUC with bout duration of | Linear regression | R = 0.947; Slope = 2.865; *p < 0.001* | n = 5 mice |

| | | | | |
|---|---|---|---|---|
| | 20% Ensure consumption with calibrated photostimulation | | | |
| S15J | AUC of OFF and ON period | Paired t-test | OFF vs ON: $p = 0.002$ | n = 5 mice |
| S15K | Slope of OFF and ON period | Paired t-test | OFF vs ON: $p < 0.001$ | n = 5 mice |
| S16E | Regression of GRAB-DA2m AUC with bout duration of 20% Ensure consumption | Linear regression | R = -0.514; Slope = 0.022; $p = 0.187$ | n = 5 mice |
| S16F | Regression of GRAB-DA2m AUC with bout duration of 20% Ensure consumption with high-intensity laser photostimulation | Linear regression | R = 0.849; Slope = 4.957; $p < 0.001$ | n = 5 mice |
| S16G | AUC of OFF and ON period | Paired t-test | OFF vs ON: $p = 0.003$ | n = 5 mice |
| S16H | Slope of OFF and ON period | Paired t-test | OFF vs ON: $p = 0.002$ | n = 5 mice |
| S16I | Licks/block | Paired t-test | OFF vs ON: $p = 0.022$ | n = 5 mice |
| S16J | Mode of ILI | Paired t-test | OFF vs ON: $p = 0.457$ | n = 5 mice |
| S16K | Proportions of bouts vs bout duration | KS test | OFF vs ON: $p < 0.001$ | n = 5 mice |
| S16L | Bout duration | Paired t-test | OFF vs ON: $p = 0.004$ | n = 5 mice |
| S16M | Bouts | Paired t-test | OFF vs ON: $p = 0.461$ | n = 5 mice |
| S16N | Licking rate | Negative binomial generalized linear mixed models | Likelihood ratio test: Factor A (Treatment): $Chi^2(20) = 437.6$, $p < 0.001$<br>Likelihood ratio test: Factor B (Time): $Chi^2(38) = 3677.2$, $p < 0.001$<br>Likelihood ratio test: A * B (Interaction): $Chi^2(19) = 186.36$, $p < 0.001$<br>Wald tests with Benjamini-Hochberg P-value adjustment:<br>0–1 s: p = 0.363<br>1–2 s: p = 0.168<br>2–3 s: p = 0.147<br>3–4 s: p = 0.136 | n = 5 mice |

| | | | | |
|---|---|---|---|---|
| | | | 4–5 s: p < 0.001<br>5–6 s: p = 0.017<br>6–7 s: p < 0.001<br>7–8 s: p < 0.001<br>8–9 s: p < 0.001<br>9–10 s: p < 0.001<br>10–11 s: p < 0.001<br>11–12 s: p < 0.001<br>12–13 s: p < 0.001<br>13–14 s: p < 0.001<br>14–15 s: p < 0.001<br>15–16 s: p < 0.001<br>16–17 s: p < 0.001<br>17–18 s: p < 0.001<br>18–19 s: p < 0.001<br>19–20 s: p < 0.001 | |
| S17B | Bout duration at first and second half sessions during the laser-OFF period | Paired t-test | First vs Second: *p = 0.653* | n = 6 mice |
| S17C | Bout duration of first and second half sessions during the laser-ON period | Paired t-test | First vs Second: *p = 0.609* | n = 6 mice |
| S17D | Bout duration at first and second half sessions during the laser-OFF period | Paired t-test | First vs Second: *p = 0.970* | n = 6 mice |
| S17E | Bout duration of first and second half sessions during the laser-ON period | Paired t-test | First vs Second: *p = 0.007* | n = 6 mice |
| S17F | Mode of ILI | Paired t-test | OFF vs ON: *p = 0.384* | n = 6 mice |
| S17G | Mode of ILI | Paired t-test | OFF vs ON: *p = 0.289* | n = 6 mice |
| S19D | Daily Ensure intake of day 1-3 semaglutide treatment during Block 1 compared to Block 2 | Repeated measures ANOVA with the Geisser-Greenhouse correction; Holm-Šídák's multiple comparisons test | Factor A (Treatment): F (1.000, 8.000) = 0.280, *p = 0.735*;<br>Factor B (Time): F (1.804, 14.43) = 7.288, *p = 0.008*;<br>A * B (Interaction): F (1.319, 10.55) = 1.893, *p = 0.200*<br>Post hoc Holm-Šídák's multiple comparisons test<br>Day 1: t = 1.418, *p = 0.418*;<br>Day 2: t = 1.527, *p = 0.418*; | n = 9 mice |

| | | | | |
|---|---|---|---|---|
| | | | Day 3: t = 0.479, *p = 0.645*; | |
| S19D | Daily Ensure intake of day 4-6 semaglutide treatment during the laser-OFF blocks compared to laser-ON blocks | Repeated measures ANOVA with the Geisser-Greenhouse correction; Holm-Šídák's multiple comparisons test | Factor A (Treatment): F (1.000, 8.000) = 0.007, *p = 0.934*;<br>Factor B (Time): F (2, 16) = 1.044, *p = 0.375*;<br>A * B (Interaction): F (2, 16) = 1.948, *p = 0.175*;<br>Post hoc Holm-Šídák's multiple comparisons test<br>Day 4: t = 0.135, *p = 0.895*;<br>Day 5: t = 1.256, *p = 0.403*;<br>Day 6: t = 1.519, *p = 0.382*; | n = 9 mice |
| S19E | Averaged Ensure intake of day 1-3 or day 4-6 semaglutide treatment during different blocks | Paired t-test | B1 vs B2 (day 1-3): *p = 0.611;*<br>OFF vs ON (day 4-6): *p = 0.934* | n = 9 mice |
| S19F | Daily bout duration of day 1-3 semaglutide treatment during Block 1 compared to Block 2 | Repeated measures ANOVA with the Geisser-Greenhouse correction; Holm-Šídák's multiple comparisons test | Factor A (Treatment): F (1, 48) = 0.042, *p = 0.839*;<br>Factor B (Time): F (2, 48) = 2.328, *p = 0.108*;<br>A * B (Interaction): F (2, 48) = 0.444, *p = 0.644*;<br>Post hoc Holm-Šídák's multiple comparisons test<br>Day 1: t = 0.761, *p = 0.718*;<br>Day 2: t = 1.111, *p = 0.655*;<br>Day 3: t = 0.140, *p = 0.892*; | n = 9 mice |
| S19F | Daily bout duration of day 4-6 semaglutide treatment during the laser-OFF blocks compared to laser-ON blocks | Repeated measures ANOVA with the Geisser-Greenhouse correction; Holm-Šídák's multiple comparisons test | Factor A (Treatment): F (1.000, 8.000) = 1.578, *p = 0.244*;<br>Factor B (Time): F (1.123, 8.981) = 1.773, *p = 0.219*;<br>A * B (Interaction): F (1.777, 14.22) = 1.018, *p = 0.377*;<br>Post hoc Holm-Šídák's multiple comparisons test<br>Day 4: t = 0.100, *p = 0.923*;<br>Day 5: t = 0.939, *p = 0.610*;<br>Day 6: t = 1.909, *p = 0.253*; | n = 9 mice |
| S19G | Averaged bout duration of day 1-3 or day 4-6 semaglutide treatment during different blocks | Paired t-test | B1 vs B2 (day 1-3): *p = 0.860;*<br>OFF vs ON (day 4-6): *p = 0.244* | n = 9 mice |

| | | | | |
|---|---|---|---|---|
| S19H | Daily bout numbers of day 1-3 semaglutide treatment during Block 1 compared to Block 2 | Repeated measures ANOVA with the Geisser-Greenhouse correction; Holm-Šídák's multiple comparisons test | Factor A (Treatment): F (1.000, 8.000) = 0.001, *p = 0.972*; | n = 9 mice |
| | | | Factor B (Time): F (1.852, 14.81) = 2.205, *p = 0.148*; | |
| | | | A * B (Interaction): F (1.591, 12.73) = 0.331, *p = 0.676*; | |
| | | | Post hoc Holm-Šídák's multiple comparisons test<br>Day 1: t = 0.517, *p* = 0.945;<br>Day 2: t = 0.370, *p* = 0.945;<br>Day 3: t = 0.326, *p* = 0.945; | |
| S19H | Daily bout numbers of day 4-6 semaglutide treatment during the laser-OFF blocks compared to laser-ON blocks | Repeated measures ANOVA with the Geisser-Greenhouse correction; Holm-Šídák's multiple comparisons test | Factor A (Treatment): F (1.000, 8.000) = 2.459, *p = 0.156*; | n = 9 mice |
| | | | Factor B (Time): F (1.258, 10.07) = 0.649, *p = 0.474*; | |
| | | | A * B (Interaction): F (1.686, 13.49) = 0.285, *p = 0.720*; | |
| | | | Post hoc Holm-Šídák's multiple comparisons test<br>Day 4: t = 0.640, *p = 0.788*;<br>Day 5: t = 1.993, *p = 0.225*;<br>Day 6: t = 0.543, *p = 0.788*; | |
| S19I | Averaged bout numbers of day 1-3 or day 4-6 semaglutide treatment during different blocks | Paired t-test | B1 vs B2 (day 1-3): *p = 0.972;*<br>OFF vs ON (day 4-6): *p = 0.156* | n = 9 mice |
